# The GH19 Engineering Database: Sequence diversity, substrate scope, and evolution in glycoside hydrolase family 19

**DOI:** 10.1371/journal.pone.0256817

**Published:** 2021-10-26

**Authors:** Marco Orlando, Patrick C. F. Buchholz, Marina Lotti, Jürgen Pleiss

**Affiliations:** 1 Department of Biotechnology and Biosciences, University of Milano-Bicocca, Milan, Italy; 2 Institute of Biochemistry and Technical Biochemistry, University of Stuttgart, Stuttgart, Germany; Weizmann Institute of Science, ISRAEL

## Abstract

The glycoside hydrolase 19 (GH19) is a bifunctional family of chitinases and endolysins, which have been studied for the control of plant fungal pests, the recycle of chitin biomass, and the treatment of multi-drug resistant bacteria. The GH19 domain-containing sequences (22,461) were divided into a chitinase and an endolysin subfamily by analyzing sequence networks, guided by taxonomy and the substrate specificity of characterized enzymes. The chitinase subfamily was split into seventeen groups, thus extending the previous classification. The endolysin subfamily is more diverse and consists of thirty-four groups. Despite their sequence diversity, twenty-six residues are conserved in chitinases and endolysins, which can be distinguished by two specific sequence patterns at six and four positions, respectively. Their location outside the catalytic cleft suggests a possible mechanism for substrate specificity that goes beyond the direct interaction with the substrate. The evolution of the GH19 catalytic domain was investigated by large-scale phylogeny. The inferred evolutionary history and putative horizontal gene transfer events differ from previous works. While no clear patterns were detected in endolysins, chitinases varied in sequence length by up to four loop insertions, causing at least eight distinct presence/absence loop combinations. The annotated GH19 sequences and structures are accessible via the GH19 Engineering Database (GH19ED, https://gh19ed.biocatnet.de). The GH19ED has been developed to support the prediction of substrate specificity and the search for novel GH19 enzymes from neglected taxonomic groups or in regions of the sequence space where few sequences have been described yet.

## Introduction

Glycoside hydrolases (GHs) form a very diverse class of enzymes catalyzing the hydrolysis and transglycosylation of glycosidic bonds, and have actually been assigned to 171 families [1]. Chitinases (EC 3.2.1.14) and lysozymes (EC 3.2.1.17) are GHs catalyzing the hydrolysis of chitin and peptidoglycan polymers, respectively [2]. Chitin, the second most abundant polysaccharide in the biosphere, is an insoluble homopolymer of β-(1–4)-linked N-acetylglucosamine (GlcNAc) monomers [3]. Peptidoglycan (or murein) is a complex polymer whose polysaccharidic component is a heteropolymer of β-(1–4)-linked GlcNAc and N-acetylmuramic acid, and is found in the cell wall of bacteria [4]. Both enzymes play fundamental biological roles: chitinases in the protection against chitin-containing organisms, in the degradation of chitinous organic matter into nutrient sources, and in autolytic morphogenetic processes in chitin-coated eukaryotic organisms [5,6], and lysozymes as antimicrobial agents in animals [7] and in autolytic morphogenetic processes in bacteria [5]. Chitinolytic enzymes have been described mainly in seven GH families (GH3, GH18, GH19, GH20, GH23, GH48, GH84) [5], lysozymes in five GH families (GH19, GH22, GH23, GH24, GH46, part of the “lysozyme superfamily”) [8]. Interestingly, families with the same enzymatic activity did not show an obvious sequence similarity, and only a few core regions are structurally conserved in the “lysozyme superfamily” [8]. Moreover, it was reported that within chitinase and lysozyme GHs promiscuity is present and some enzymes show a minor activity toward murein and chitin, respectively [9–12], despite differences in the protein fold, substrate binding residues, and catalytic mechanism among families [13].

The GH19 family contains enzymes that are endo-acting hydrolases, highly specialized either as endo-chitinases [2,6,14] or as lysozymes [15–17], but there are also enzymes that show both activities [10,18]. Thus, this family provides an ideal opportunity for a comprehensive study of sequence-structure-function relationships. Previous structural studies on GH19s have demonstrated that they have a globular α-helical fold and a catalytic core spanning a deep catalytic cleft [6]. The proposed mechanistic model of hydrolysis follows a single displacement mechanism causing inversion of the anomeric carbon (**S1 Fig**), with two glutamic acids acting as acid and as base, which activate a water molecule. The nucleophilic water molecule is coordinated by a third key residue, usually a serine or threonine [19].

In early studies, GH19 enzymes were discovered to be plant pathogenesis-related proteins with chitinase activity, and later grouped into five chitinase classes (I, II, IV, VI, VII) [5,20–23], while the two remaining classes (III-V) are part of the GH18 family, not covered in this study. Classes I and IV are linked to one accessory N-terminal carbohydrate binding module (CBM), whereas class II GH19s are characterized by the absence of a CBM [24]. The sequences of class IV enzymes are shorter than classes I and II, resulting in a smaller number of subsites in the catalytic cleft and a different substrate binding mode [14,25]. GH19s identified in *Actinobacteria* were found to be more similar to class IV and were suggested to originate from horizontal gene transfer (HGT) of class IV plant chitinases [26,27]. However, different CBMs are linked to chitinases of *Actinobacteria* and to plant chitinases of class IV [27,28]. Few studies on chitinases cite explicitly the existence of classes VI and VII [29]. Class VI chitinases were identified by similarity with some bacterial chitinases and the presence of a duplicated CBM with a long proline-rich region in their N-terminal [23], which permits them to work as lectins.

Recently, an alternative classification scheme has been proposed by dividing GH19 chitinases into “loopful” and “loopless” chitinases, based on either the presence or the absence of up to six loop insertions [24], named 1, 2, 3, 4, 5, and C-terminal in this study. Few chitinases were also detected and characterized in *Proteobacteria* [30–33]. In contrast to the classification of GH19-domain containing enzymes in different chitinase groups, some enzymes were found in phages and described as endolysins with lysozyme activity [15,16,34,35].

The main biological activity of GH19 enzymes in plants is associated with improved resistance against *Fungi* [36–38] and against phytopathogenic bacteria [10,39]. Tolerance to pests was demonstrated to increase in transgenic plants in which heterologous GH19 genes were introduced or overexpressed [40–43]. Other members of the GH19 family are involved in stress response of plants caused by wounding, drought, or high temperature [44,45], and in the regulation of lignin accumulation during plant growth [46]. As many characterized GH19s are endo-chitinases, they could be applied for the degradation of chitin to chitooligomers, which are anti-inflammatory drugs [47], and for the conversion of chitin extracted from shellfish biomass waste [5,6]. GH19 enzymes were recently modified by site-directed mutagenesis and engineered into transglycosylases [48–51]. Recently, an amphipathic region of a GH19 endolysin was shown to induce outer-membrane permeabilization in Gram-negative bacteria strains isolated from hospitalized patients [52], proving potential in inspiring new drugs to fight multi-drug resistant bacteria [53]. Thus, GH19s are interesting not only for their dual substrate specificity, but also for their promising biotechnological applications, making sequences from this family an appealing target for the discovery and optimization of novel enzymes.

Bioinformatics tools have been used since decades to identify novel enzymes by searching for genes with sequence similarity to the tiny fraction of yet biochemically characterized enzymes [54]. They include methods for analyzing sequence space and structural properties, and their evolutionary relationships, for disentangling the basis of functional evolvability, for targeting enzymes with novel functions and for the design of optimal engineering routes [55]. The approach currently applied for studying sequences and structures of GH families is based on the CAZy classification system [1]: some families have been manually split into subfamilies based on their substrate specificity, but for most families, including GH19, this information is unknown. In this paper, we apply a bioinformatics workflow to investigate the sequence space of the GH19 family, conserved positions, and evolutionary paths. This workflow is based on the GH19ED database, as part of the BioCatNet database system [56] to handle, store, and analyze sequences and structures of the GH19 family.

The obtained results were integrated with experimental data from literature, known motifs and accessory domains, to support the discovery of novel interesting GH19 enzymes from annotated (meta)genomic sequences.

## Results

### GH19ED database setup: Classification and domain annotations

**Fig 1:**
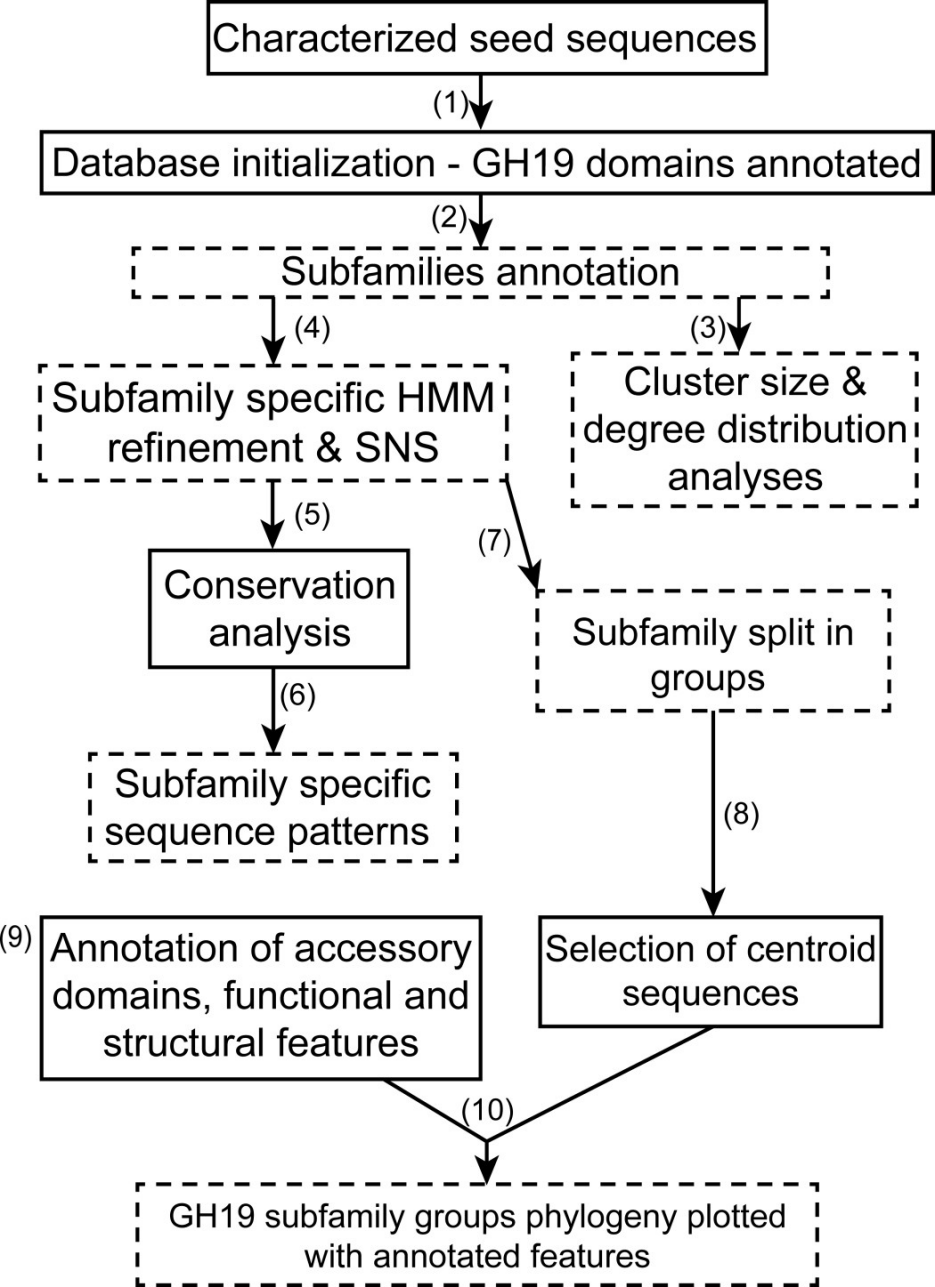
The flowchart of the workflow employed in this work for the analysis of sequence diversity and evolution of the GH19 family. Initially, CAZy and literature screenings were employed to identify the characterized seed sequences. BLAST searches of all seed sequences were conducted and sequences shorter than 120 amino acids were removed. The obtained matches were used to create the GH19ED database, in which the GH19 catalytic domain was annotated with an available profile hidden Markov model (HMM) from the Pfam database (1). Then, catalytic domain sequence networks were obtained by all-vs.-all pairwise aligned sequences and a threshold of 40% identity (2) that permitted to identify subfamilies containing enzymes specialized in one type of activity. Subfamilies were annotated in the database. The properties of the GH19 domain sequence space were also investigated by the analysis of network properties obtained at varying identity thresholds (3). Then, a representative sequence was defined for each subfamily, and an alignment with other characterized members (4) was used for new profile HMMs, to define a standard numbering scheme (SNS) to identify homologous sites within each subfamily. An independent evolutionary conservation analysis with Rate4Site was done for each subfamily (5); by aligning the sequences and structures of the most conserved sites between subfamilies (6), sequence patters specific for each subfamily were identified. Each subfamily was further split into groups from catalytic domain sequence networks, by choosing a 60% identity threshold (7) and these groups were annotated in the database. By functional and structural motifs defined in literature and profile HMMs available for accessory binding modules (8), other annotations were inserted into the database In the final step, GH19 catalytic domain sequences from each group were clustered to select representative centroids (9) to build a large-scale phylogeny, in order to investigate the evolution of structural features, previously annotated and extracted from the database (10). The panels with a dashed outline represent results generated in this study. *Structural information in this study refers to chitinase loops, the endolysin 3-helix peptidoglycan binding bundle and accessory binding modules.

An overview of the workflow applied for this study is shown in **Fig 1**. In total, 23,853 sequences were retrieved by using 80 seed sequences (https://doi.org/10.18419/darus-804) for BLAST searches in the NCBI non-redundant protein database and in the PDB. For all seed proteins, either the structure or the enzymatic activity was reported (**S1 Table**). Sixty-seven seed sequences were obtained from CAZy, 13 were found by screening literature (see *Methods* section). The sequences were annotated and filtered with the GH19 profile hidden Markov model (HMM) from Pfam. Sequences shorter than 120 amino acids were considered as fragments and removed from the database, resulting in 22,461 sequence and 16,120 protein entries in the GH19ED database (https://doi.org/10.18419/darus-1163). The distribution of lengths is trimodal, with maxima at ~200, ~280 and ~580 amino acids, and a long tail with longer sequences (**S2 Fig**). The distributions of pairwise sequence identities for the catalytic domains of chitinases and endolysins, however, are unimodal with peaks at around 30% (**S3 Fig**).

**Fig 2:**
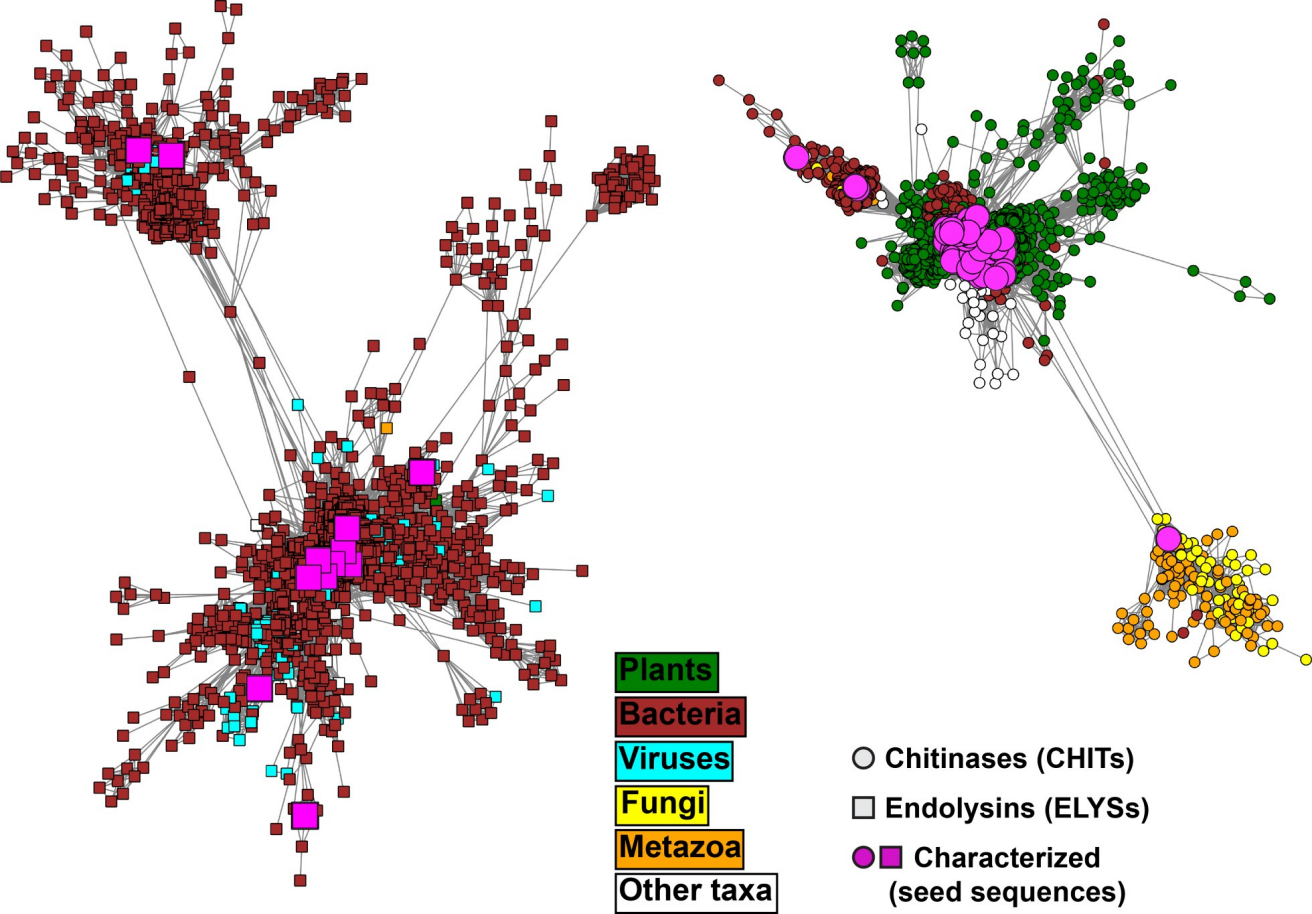
Protein sequence networks of representative domains of the two bigger clusters containing seed sequences (5067 nodes, 2329 nodes on the left for CHITs, chitinases, and 2738 nodes on the right for ELYSs, endolysins) connected by edges with a sequence identity threshold of 40%. The prefuse force-directed OpenCL layout with respect to the edge weights was used for network visualization. The domains were extracted by scanning the sequences collected through BLAST searches (using the seed sequences reported in **S1 Table** as queries) with the GH19 profile HMM PF00182 from Pfam. Nodes are colored according to their annotated taxonomic source. The remaining smaller network clusters are visualized in **S5 Fig**.

The annotated GH19 domains were extracted from the database and clustered. The domain sequences of each cluster have less than 90% identity with respect to a centroid sequence of each cluster. Domain-based sequence networks were built by considering each sequence as a node and the percent identity from global pairwise sequence alignment with any other centroid sequence as the weight of the edges connecting them. Edges were defined only when sequence identity was higher or equal to 40%. At that threshold two large networks represent 19,521 sequence entries (87% of all entries in GH19ED database), including all the biochemically characterized seed sequences (**S4 Fig**). The sequences within these two large networks (**Fig 2**) were assigned to two separate subfamilies, chitinases (CHITs, 8554 sequences) and endolysins (ELYSs, 10,967 sequences), considering that all seed sequences nested in CHITs and ELYSs were previously characterized as specialized chitinases and endolysins, respectively (**S1 Table**). The sequences in small networks (less than a few tens of nodes) without characterized seeds were included in the database, but not further analysed.

**Fig 3:**
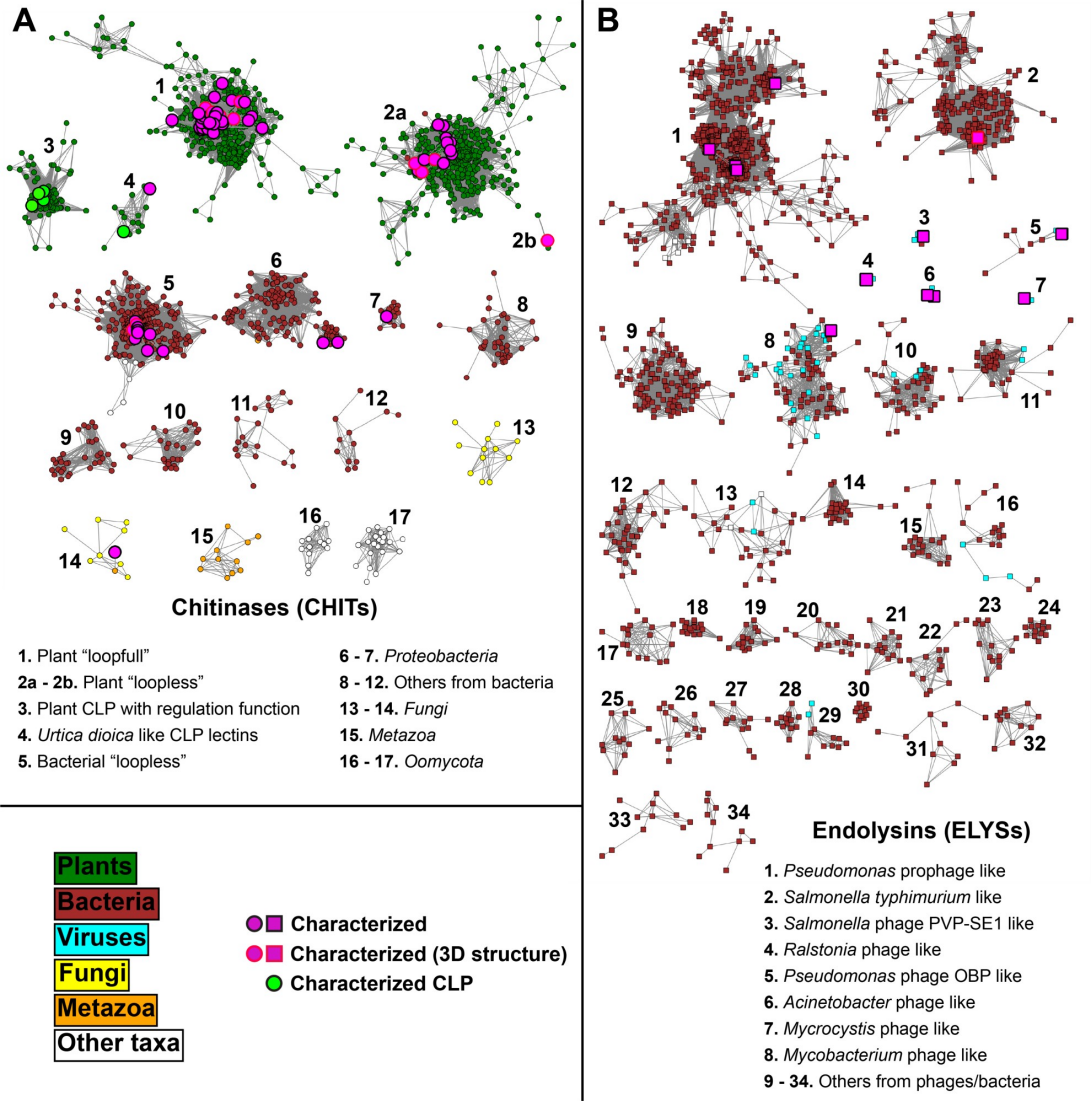
Protein sequence networks of representative domains of CHITs (A) and ELYSs (B) (1860 nodes for CHITs and 1521 nodes for ELYSs, respectively), connected by edges with a sequence identity threshold of 60%. The prefuse force-directed OpenCL layout with respect to the edge weights was used for network visualization. The domains were extracted by using profile HMMs of CHITs and ELYSs (generated in this study) to scan the sequences in the GH19ED database. Nodes are colored according to their annotated taxonomic source. Seed sequences are highlighted, with a different border if a structure is available in the PDB. Nodes representing characterized “chitinase-like” proteins (CLPs) are also highlighted and presented in **S3 Table**.

Two separate standard numbering schemes [57] were generated for the CHIT and the ELYS subfamily. A standard numbering scheme is applied to assign unique standard position numbers to all structurally (and functionally) equivalent positions and is independent of the numbering on sequence level or in the PDB entry. Standard position numbers of this numbering scheme are generated by alignment of all sequences to a sequence profile and by transferring the sequence position numbers of the profile reference protein to other profile-aligned sequences. Thus, the CHIT subfamily profile HMM was obtained by aligning the sequences of 14 chitinases with known X-ray structure and 44 biochemically characterized chitinases (https://doi.org/10.18419/darus-803). The “loopful” chitinase from *Secale cereale* (rye seed, PDB accession 4j0l) was selected as the reference for numbering the HMM sites. The chosen reference enzyme possess a complete experimental mapping of substrate binding subsites [58], and annotated chitinase loops. The numbering covers the catalytic domain from position 24 to 266 of the reference sequence (the first 23 amino acids are the N-terminal signal peptide). The obtained CHIT profile HMM was used to reannotate CHIT catalytic domains, and new networks of the refined CHITs domain centroids were generated, with edges defined at a threshold of 60% sequence identity (**Fig 3A**). The 18 resulting CHIT clusters allowed to split the CHIT subfamily into 17 groups (**S2 Table**). Two groups include “loopful” or classes I—II chitinases (CHIT 1) and “loopless” or class IV chitinases (CHIT 2a-b). Clusters 2a and 2b were merged into one group, because both contain sequences characterized as class IV “loopless” chitinases from plants (CHIT 2a) or from *Bryophyta* (CHIT 2b). The term “plant” is used to indicate *Embryophyta*, with two exceptions from the green algae *Klebsormidium nitens* in CHIT 1. Two smaller groups (CHIT 3 and 4) contain plant proteins characterized as non-enzymatic (or chitinase-like proteins, CLP): these are listed and referenced in **S3 Table**. Eight CHIT groups include bacterial sequences. The main cluster (CHIT 5) contains the most characterized group of bacterial “loopless” chitinases (class IV bacterial chitinases according to previous classification), two clusters contain sequences mainly from *Proteobacteria* species (CHIT 6 and 7), and five clusters form small groups (CHIT 8 to 12) from different bacterial sources. It is interesting to note that not all bacterial “loopless” chitinases (CHIT 5) are from *Actinobacteria* species (> 90%), but also from *Myxococcales* (> 3%), *Firmicutes* (> 1%), *Betaproteobacteria* (> 1%), and *Gammaproteobacteria* (> 1%), enriched in species typically found in soils. Five groups (CHIT 13 to 17) contain only a few tens of sequences from *Fungi*, *Metazoa*, and *Oomycota*, with the only characterized fungal chitinase in CHIT 14.

A standard numbering scheme for the ELYS subfamily was created from a profile HMM obtained by aligning the sequences of twelve biochemically characterized endolysins and other sequences retrieved as indicated in the *Methods* section (https://doi.org/10.18419/darus-803). The endolysin from the bacteriophage SPN1S of *Salmonella typhimurium* [59], the only ELYS protein with a known structure (PDB accession 4ok7), was selected as reference. The standard numbering covers the catalytic domain from position 1 to 209 of the reference sequence and was used to reannotate ELYS domain sequences. Refined catalytic domain centroids were obtained as above (**Fig 3B**). Based on the resulting networks, ELYS sequences were assigned to 34 groups from bacteria or viruses (**S2 Table**). Eight of these groups contain at least one characterized seed sequence, and only two of them contain thousands of sequences. One of these large groups (ELYS 2) contains the reference seed endolysin from *Salmonella typhimurium* phage, and the other group (ELYS 1) contains four seed endolysins from *Pseudomonas* phage/prophages. One group in the range of 100–1000 sequences contains a seed endolysin from *Mycobacterium* phage seed (ELYS 8), while five other groups of the same size contain only uncharacterized putative endolysins. The remaining ELYS groups are small and contain between 1 and 100 sequences. Five of these contain at least one characterized seed sequence.

The length distributions of the CHIT and the ELYS GH19 domains are bimodal (**S5 Fig**), with peaks at 200 and 245 amino acids for CHITs, and at 175 and 200 amino acids for ELYSs. The length distribution of full-length sequences (**S2 Fig**) suggests that peaks between 160 and 250 amino acids contain single domain proteins, whereas the peaks between 540 and 620 amino acids contain proteins with two or more domains: by looking into the GH19ED database, in this case one is always a CHIT and never an ELYS domain. Sequences longer than 620 amino acids are highly modular proteins that include at least one GH19 domain associated to other uncharacterized domains. Most of the CHIT sequences (51%) of the peak between 540 and 620 amino acids contain three domains: a putative, uncharacterized domain followed by a CHIT domain and a CBM5/12 domain. The bigger fraction of these sequences (40%) consists of an uncharacterized domain and a CHIT domain, 6% of a CHIT domain and two CBM5/12 domains, either at the N- or C-terminus. Only five sequences include a CBM18 domain at the N-terminus followed by two CHIT domains, or a CBM18 domain between two CHIT domains. One sequence consists of two CBM18-CHIT tandem domains, and one sequence contains two CHIT domains without additional domains.

CBMs are associated only to CHIT domains, but not to ELYS domains (**S6 and S7 Figs**). CBM5/12 domains are associated to bacterial chitinases, CBM13 to a few members of “loopless” bacterial chitinase (CHIT 5), and some *Cyanobacteria* chitinases (CHIT 11) are associated to LysM. Most of the plant CHIT groups are associated to CBM18, except for a group of plant CLP with regulatory functions (CHIT 3). Other eukaryotic and three distinct bacterial groups (CHIT 8 and 11 to 17) do not contain any known CBM.

Only two accessory binding modules, LysM or PG_binding_1, could be retrieved associated to at least ten ELYS sequences (**S7 Fig**). PG_binding_1 is the most frequent domain, present in the sequences of the two small groups ELYS 15 and 21 and in a few sequences in ELYS 1, 5, 12, 13, 14, 20, and 31. LysM was found in most of the sequences of the two small ELYS groups ELYS 13 and 22, and a few sequences of the largest group ELYS 1.

### Properties derived from sequence networks

Sequence networks of the catalytic domains were obtained also by applying different thresholds of sequence identity to calculate the degree and the cluster size distributions, in order to identify groups of highly connected domain sequences (hub regions), and to estimate the overall connectedness of the domain sequences. Both distributions depend on the applied threshold of sequence identity. The distribution *N(n)* of the degree *n* (i.e. number of neighbouring sequences) was approximated by a power-law function *N(n) ~ n*^*-γ*^ with *γ* = 1.1 for *n ≤* 50 at a sequence identity threshold of 95% (**S8 Fig**). Thus, two hub regions were identified (CHIT 6 and ELYS 1), where the domain sequences are densely connected to their neighbours (**S4 Table**). The histograms for the distributions of the number *N(s)* of clusters with cluster size *s* at thresholds of 60%, 70%, 80%, and 90% pairwise sequence identity were approximated by a power-law function *N(s) ~ s*^*-τh*^ (**S9 Fig**). The slope τ_h_ represents the ratio of small to large clusters and thereby indicates the connectedness of the GH19 domain sequence space, with an extrapolated exponent of *τ* = 1.1 (**S10 Fig**).

### Conservation analysis of catalytic domains

**Fig 4:**
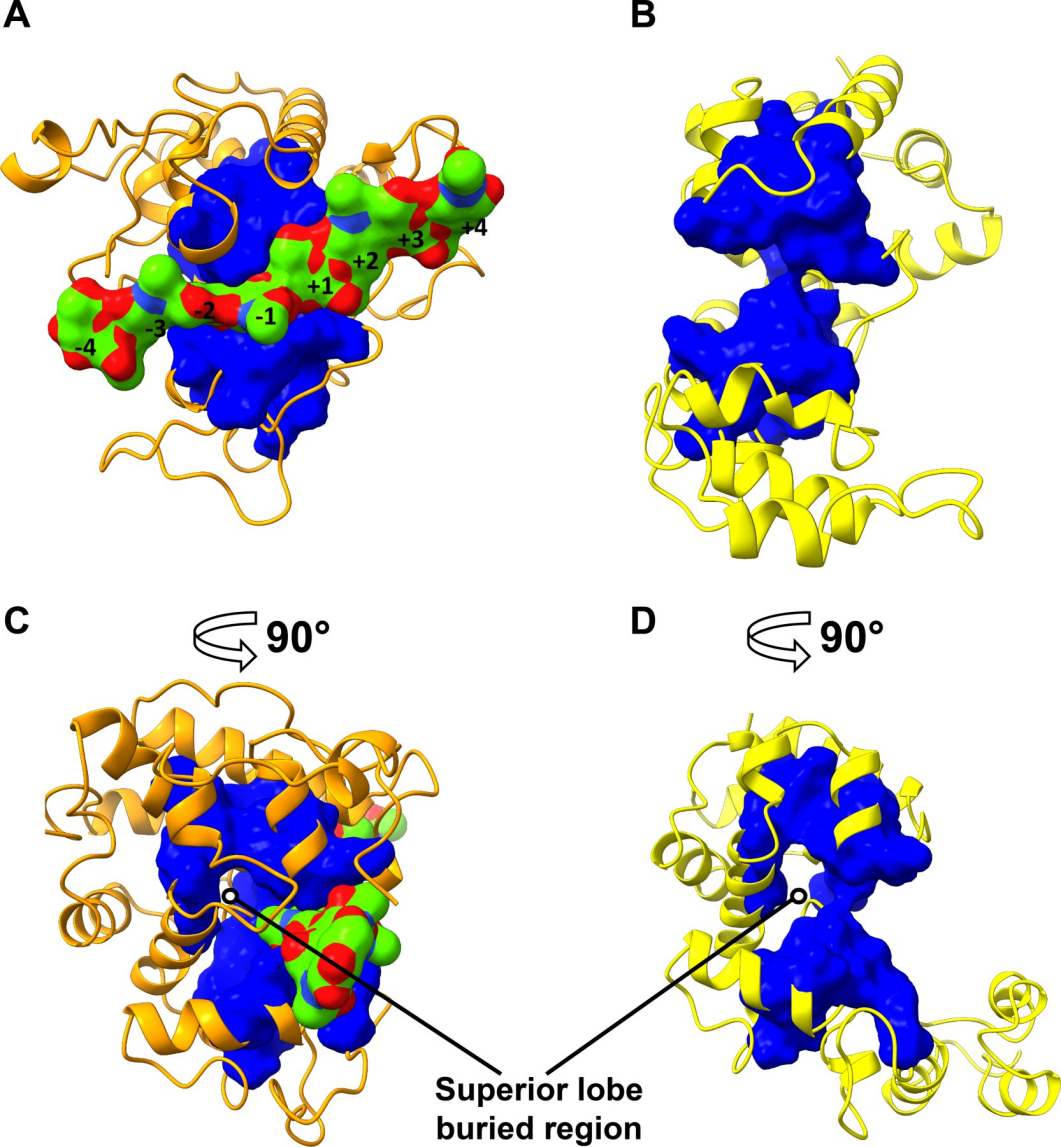
The most conserved and structurally aligned positions between CHITs and ELYSs (reported in Table 1). The solvent accessible surface of these positions is plotted onto the reference models of CHIT (**A**) and ELYS (**B**) subfamilies (PDB accessions 4j0l of “loopful” plant chitinase from rye seed *Secale cereale*, and 4ok7 of bacteriophage SPN1S endolysin from *Salmonella typhimurium*, respectively), represented in cartoon style. In (**C**) and (**D**), the same models are rotated by 90° around the vertical axis.

Seventy seven of the 242 positions in CHITs (**S5 Table**) and 51 of the 209 positions in ELYSs (**S6 Table**) had the highest conservation score of 5, as determined by Rate4Site (**S11 Fig)**. Most of these residues are in the substrate binding cleft (blue in **S12 Fig**), whereas the most variable positions are in the loops at the extremity of the catalytic cleft or at the surface of the two lobes (red in **S12 Fig**). The structural alignment of the highly conserved positions in CHITs (**Fig 4A**–**4C**) and ELYSs (**Fig 4B**–**4D**) highlights the presence of a conserved and shared GH19 core of 26 positions spanning the catalytic centre and the internal part of each lobe. In contrast, the most buried part of the GH19 domain, behind the layer that delimits the surface of the substrate binding cleft in the superior lobe, is conserved in each subfamily, but it is not shared among them. The conserved core comprises the catalytic and the key water coordinator residue (E69, E87, S120 and E49, E58, T130 for CHITs and ELYSs, respectively) and the substrate binding residues at subsites -2, -1, and +1 (**Table 1**). Another position predicted to bind the substrate at subsite +1 (standard positions E203 and N191 in CHITs and ELYSs, respectively) has the highest conservation, but it was not identified as part of the shared core since it contains a gap in more than 10% of aligned ELYS sequences (**S6 Table**). In contrast, the positions that bind the substrate at subsites -4, -3, +2, +3 and +4 were not conserved, neither in CHITs nor in ELYSs (**Table 1**). Two patterns of residues were identified, which are specific for the two subfamilies: six positions in CHITs and four positions in ELYSs (**Table 2**).

**Table 1:** Conserved core shared in CHIT and ELYS subfamilies. Structurally aligned positions are listed in each row, numbered according to each subfamily-specific standard numbering scheme (see *Methods* section). Information is provided about the percentage of conserved residues if higher than 5%.

CHIT standard position	CHIT Amino acid distribution			ELYS standard position	ELYS Amino acid distribution			Function^a^
58	A 80%	V 11%	I 4.2%	40	A 90%	S 2.1%	C 1.8%	
59	A 63%	T 32%		41	A 36%	M 34%	Y 9.3%	
60	F 61%	M 19%	A 16%	42	F 82%	W 4.5%	M 3.7%	
62	A 82%	G 12%	T 1.8%	44	A 91%	G 4.3%	S 3.4%	
63	H 52%	N 25%	Q 19%	45	Q 88%	T 9.3%		
66	H 54%	Q 34%	S 3.6%	48	H 93%	V 2.3%		Substrate binding (+1)
67	E 91%	K 4.7%		49	E 99%			Catalytic proton donor and substrate binding (-1)
68	T 91%	S 5.4%		50	S 86%	T 11%	C 1.4%	
89	E 94%			58	E 99%			Catalytic base and substrate binding (-1)
96	Y 90%	K 1.7%	M 1%	106	Y 95%	F 2.3%		Substrate binding (-1)
113	G 99%			123	G 99%			
114	R 98%	K 1.4%		124	R 95%	G 1.2%	A 1.2%	
115	G 99%			125	G 96%	T 2.5%		
118	Q 91%	P 4.8%	M 3.2%	128	Q 92%	M 5.5%	G 1%	Substrate binding (+1)
120	S 84%	T 9.6%	Y 4.3%	130	T 99%			Water coordination and substrate binding (-2)
124	N 99%			134	N 96%			Substrate binding (-2)
125	Y 99%			135	Y 97%	F 1.5%		
140	P 100%			150	P 95%	G 1.2%		
143	V 91%	I 4.2%	L 3.8%	153	L 74%	A 13%	V 7.8%	
154	A 85%	G 13%	S 1.3%	163	A 83%	S 4.2%	E 1.9%	
158	W 66%	F 30%	Y 2.3%	167	W 65%	F 14%	Y 10%	
195	I 63%	T 29%	M 4.3%	183	T 81%	R 9.6%	S 3.6%	
198	I 88%	L 8.2%	V 2.1%	186	I 85%	V 12%		Substrate binding without side chain (-2)
199	N 92%	Y 4.8%		187	N 97%			Substrate binding (-2)
200	G 94%	S 2.3%	A 1.8%	188	G 89%	L 3.3%	P 1.5%	
215	R 92%	I 4.3%		196	R 89%			Substrate binding (+1)

^**a**^Binding subsites (in parentheses) are numbered according to the standard nomenclature; cleavage occurs between the sugar units bound at subsites -1 and +1 [60].

**Table 2:** Frequency distributions of amino acids at standard positions used to define sequence patterns specific for CHIT and ELYS subfamilies. Information is provided about the percentage of conserved residues at each subfamily specific standard numbering scheme position if higher than 5%.

CHIT Standard position	CHIT Amino acid distribution			ELYS Standard position	ELYS Amino acid distribution		
97	C 93%			33	I 80%		
105	C 91%			109	R 80%	E 5.4%	
151	F 41%	L 32%	W 23%	118	G 97%		
190	G 95%			173	L 54%	Y 23%	
192	G 95%						
222	F 39%	Y 34%	L 15%				

### Loops in CHITs

The standard numbering scheme for CHITs was applied to annotate the start and the end of each of the six chitinase loops in all sequences of the GH19ED. The naming convention of the six loops is based on the comparison of “loopful” and “loopless” chitinases in a structural alignment (**S13A Fig**), which resembles the definition reported previously [50]. Loops 2, 3 and 5 are the longest and vary widely in length (**S14 Fig**). Analysis of the loop length distribution showed two groups with different length for loop 2 (from 10 to 16 residues and from 18 to 23 residues) and for loop 3 (from 12 to 20 residues and from 22 to 31 residues). The longer loops were found only in a *Proteobacteria* group (CHIT 6). Except for loops 3 and 4, the conservation score of the loops is low (**S7 Table**). The substrate binding sites located on loops are not conserved, except for standard position 96 on loop 3.

The pattern of presence or absence of loops was described by a binary loop code (**Table 3**). The loops with the higher sequence conservation are also the ones that are present in more groups, with loop 3 present in all except for CHIT 7. The first three loops could not be annotated for CHIT 7, because the N-terminal catalytic domain of CHIT 7 is not homologous to the N-terminus of the profile HMM used for the CHIT standard numbering scheme. The loop combinations of the other five loops vary between groups (**Table 3**). All six loops are present in the "loopful" plant CHIT 1, plant CLP with regulatory functions (CHIT 3), and a small bacterial group (CHIT 12). Other bacterial chitinases (CHIT from 6 to 11) lack at least loop 1. Loop 5 is absent in *Urtica dioica*-like CLP lectins (CHIT 4). Loops 1 and 3, but not loop 4, are present in most of the "loopless" plant CHITs (CHIT 2a-b), whereas loops 3 and 4 are present in the "loopless" bacterial CHIT 5 and, with some variations, in CHIT 13–14 from *Fungi*, CHIT 15 from *Metazoa*, and CHIT 16–17 from *Oomycota*.

**Table 3:** Frequency distributions of loop annotations among CHIT groups. Names are defined according to **Fig 3A** (occurrences not displayed if below 5%). h-fam = homologous family (group) name in the GH19ED; ID = group identifier. Binary loop code: ‘0’ = absent; ‘1’ = present; ‘-‘ = undefined.

CHIT h-fam (ID)	Loop 1	Loop 2	Loop 3	Loop 4	Loop 5	Loop C-terminal	Binary loop code
Plant “loopful” (1)	88.2%	93.1%	88.7%	97.8%	96.7%	91.8%	1 1 1 1 1 1
Plant “loopless” (2)	95.4%		99.8%	5.6%			1 0 1 0 0 0
Plant CLP with regulation function (3)	90.5%	95.5%	97.0%	100%	99.8%	94.5%	1 1 1 1 1 1
*Urtica dioica* like CLP lectins (4)	96.8%	96.8%	100%	100%%		71.0%	1 1 1 1 0 1
Bacteria“loopless” (5)			99.9%	99.8%			0 0 1 1 0 0
*Proteobacteria* (6)		51.7% ^**a**^47.4%	^**a**^99.1%	99.7%	99.6%	98.0%	0 1 1 1 1 1
*Proteobacteria* (7)				100%	100%	93.3%	0 0 0 1 1 1
Bacteria (8)		97.0%	98.5%	100%	98.5%	76.5%	0 1 1 1 1 1
Bacteria (9)		100%	100%	100%	100%	78.6%	0 1 1 1 1 1
Bacteria (10)		100%	100%	100%	100%	15.1%	0 1 1 1 1 0
Bacteria (11)	17.9%	53.6%	100%	78.6%	60.7%	42.9%	- - 1 1 - -
Bacteria (12)	93.0%	100%	100%	100%	100%	100%	1 1 1 1 1 1
*Fungi* (13)			100%	73.3%		66.7%	0 0 1 1 0 1
*Fungi* (14)		30%	60%	88.9%			0 0–1 0 0
*Metazoa* (15)			100%	22.2%			0 0 1 0 0 0
*Oomycota* (16)			95.6%	98.5%			0 0 1 1 0 0
*Oomycota* (17)	19.6%		100%	97.8%			0 0 1 1 0 0

^**a**^This fraction of sequences has a longer loop, based on length distribution reported in **S14 Fig**.

### Peptidoglycan binding module in ELYSs

The presence of a 3-helix bundle peptidoglycan binding module (PBM) was reported previously [59] as a binding motif that covers standard positions 59 to 106 in the catalytic domain of the characterized endolysin from bacteriophage SPN1S (**S13B Fig**). The PBM sequence conservation is 1.8, which is low, as the minimum is 1 and the maximum 5. The sequences harbouring a PBM are in ELYS 2 and 19. In addition, a PBM is present in a few sequences of ELYS 1.

### Phylogenetic analysis of the GH19 family

**Fig 5:**
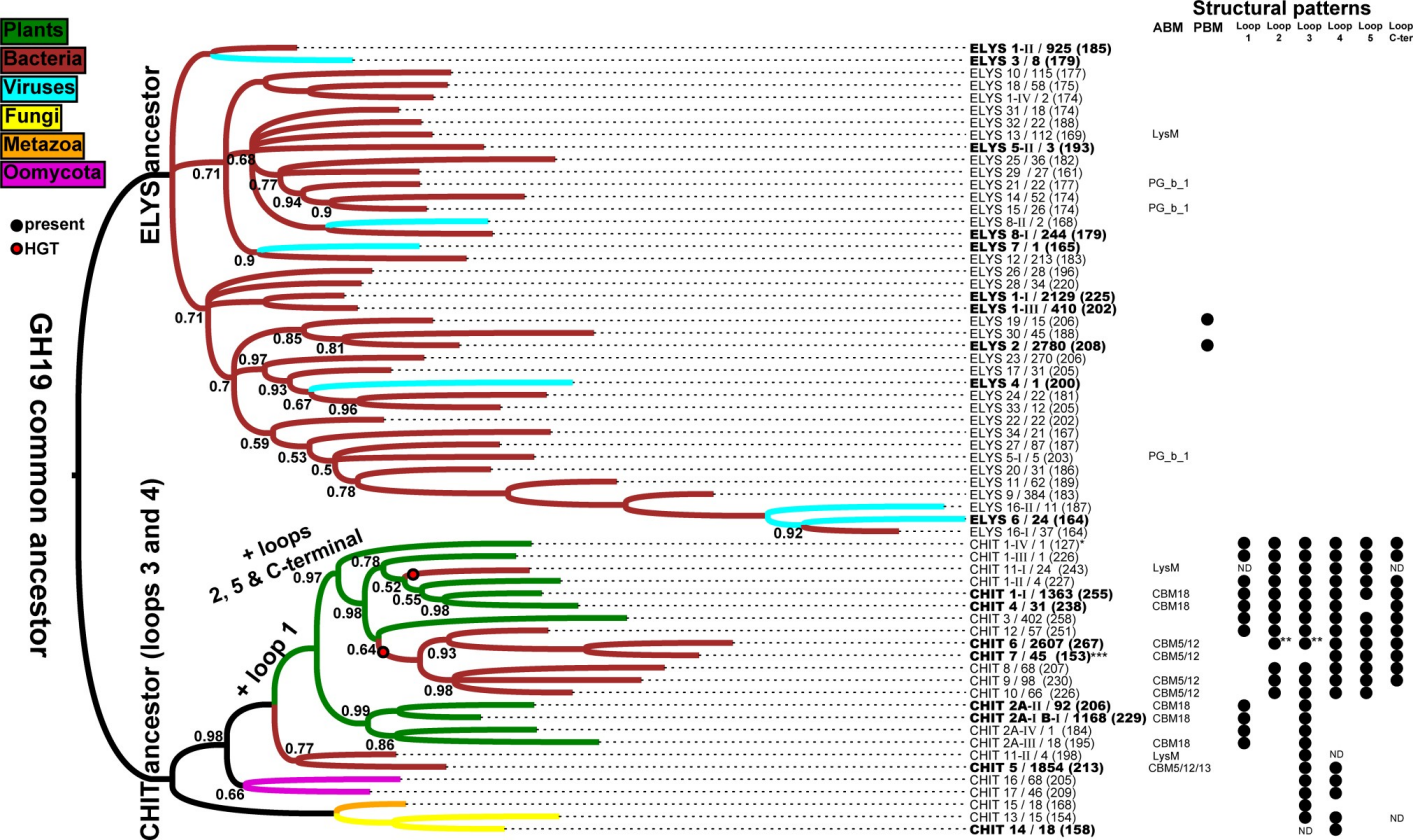
Phylogeny of centroids representative of GH19 sequence space, plotted on structural patterns analyzed in this study. Sequences are indicated with the respective subfamily name (ELYS or CHIT) followed by the group identifier (homologous family in GH19ED database) / number of represented sequences, followed by sequence length of the centroid in parentheses. Sub-clusters according to **S15** and **S16 Figs** are reported as Roman numerals. Sequences representing clusters that contain characterized seed sequences are depicted in bold. *This centroid sequence is a fragment. **A fraction of sequences from group CHIT 6 has longer loops (see **Table 3**). ***All sequences from group CHIT 7 have a different N-terminal portion in their catalytic domain. HGT = horizontal gene transfer; ABM = accessory binding module; PBM: 3-helix peptidoglycan binding bundle; PG_b_1 = PG_binding_1; CBM = carbohydrate binding module; ND = not defined because too variable within the group (homologous family). The numbers at internal nodes indicate posterior probabilities only if < 1; internal nodes are collapsed if posterior probability is less than 0.5. ELYS 1 = *Pseudomonas* prophage like; ELYS 2 = *Salmonella typhimurium* like; ELYS 3 = *Salmonella* phage PVP-SE1 like; ELYS 4 = *Ralstonia* phage like; ELYS 5 = *Pseudomonas* phage OBP like; ELYS 6 = *Acinetobacter* phage like; ELYS 7 = *Mycrocystis* phage like; ELYS 8 = *Mycobacterium* phage like; ELYS 9 to 34 = other putative endolysins from phages and prophages; CHIT 1 = plant “loopful”; 2a-b = plant “loopless”; CHIT 3 = plant CLP with regulatory function; CHIT 4 = *Urtica dioica* like CLP lectins; CHIT 5 = bacterial “loopless”; CHIT 6–7 = *Proteobacteria*; CHIT 8 to 12 = other putative bacterial chitinases; CHIT 13–14 = *Fungi*; CHIT 15 = *Metazoa*; CHIT 16–17 = *Oomycota*.

Clustering of the groups resulted in 64 representative GH19 catalytic domain centroids, with four ELYS (1, 5, 8 16) and three CHIT (1, 2, 11) groups divided in at least two sub-clusters and represented by more than one centroid (**S15 and S16 Figs**). A phylogenetic tree was built to study GH19 evolutionary relationships at a large scale with respect to biochemical properties and the annotated structural features (**Fig 5**). The result of this analysis confirms that the groups of the two GH19 subfamilies have two distinct common ancestors. In the ELYSs branch, 34 out of 40 sequences are of bacterial origins, probably in regions associated to phages or prophages. The ELYS 2 and 19, which contain the PBM in their catalytic domain, share a common ancestor with ELYS 30 that does not possess it. The lineages of eukaryotic CHIT groups (*Fungi* CHIT 13–14, *Metazoa* CHIT 15 and *Oomycota* CHIT 16–17) seem to have separated very early, before the diversification of the bigger groups of plant and bacterial chitinases, which share a more recent common ancestor. CHIT 5 (bacterial “loopless” chitinases) and CHIT 11-II separated before the evolution of plant chitinases. Plant CHITs seem to have the same common ancestor and separated along the “loopful” (CHIT 1) and “loopless” lineages (CHIT 2); other plant groups of CLPs (CHIT 3–4) diversified from the “loopful” lineage. The tree indicates that some bacterial lineages of CHITs (CHIT 6 to 12 and CHIT 11-I) were transferred from “loopful” plant lineages to bacteria through two independent horizontal gene transfers (HGTs). Even if the posterior probabilities for the nodes corresponding to these HGTs are low (0.5–0.6), a high probability supports the plant clade in which bacterial lineages are nested. This observation suggests that these bacterial CHITs diversified from plant ancestral CHITs. Interestingly, the two centroid sequences of CHIT 11, divided in two sub-clusters (CHIT 11-I and CHIT 11-II), show different phylogenetic histories. This and other issues related to GH19 evolution with respect to the annotated structural features will be exposed in the *Discussion* section.

## Discussion

### Extended classification of the GH19 sequence space

The catalytic domain of the biochemically characterized GH19 seed sequences of chitinases and endolysins (**S1 Table**) was separated into two subfamilies, CHITs and ELYSs, based on a sequence identity threshold of 40%. Most of the characterized GH19 (68 out of 80) are CHITs, mainly from plants (52), whereas the 12 characterized ELYSs are phage or prophage endolysins. The fact that most uncharacterized ELYSs are assigned to bacterial species in public databases might be caused by the internalization of phage sequences in the genome of the bacterial host.

Before the introduction of the CAZy classification system for GHs [2], GH19 chitinases have been assigned to five classes by employing different criteria [5,20–22]. Later, CHITs were divided in "loopful" and "loopless" chitinases [24], without considering the entire sequence space of CHITs. The extended classification proposed in this paper is based univocally on sequence identity of the GH19 domain, and the identity threshold used for clustering was adjusted to be in accordance with the previous assignment into five classes and into the two loop types (**S2 Table**). According to class classification, chitinases of class I—II ("loopful") are from plants and distinguished by the presence or absence of an N-terminal CBM: based on this study, it is suggested to assign them to the same group (CHIT 1), as proposed also in [61], but never followed in more recent works. Class IV ("loopless") is separated into CHIT 2a-b (plant source) and CHIT 5 (bacterial source). Some sequences from classes VI and VII were found in CHIT 4 and CHIT 3, respectively, and were characterized as lectins (CHIT 4) or as regulative (CHIT 3) CLPs (**S3 Table**). Therefore, the other sequences of the families CHIT 4 and CHIT 3 were predicted to be putative CLPs rather than chitinases. Most of the class VII sequences found in literature are members of group CHIT 3. Therefore, we predict that they are catalytically inactive or may possess at most low catalytic activity (as recently showed in [62]), due to the deletion of the catalytic glutamic acid or its exchange, mostly by lysine. Similarly, in all CHIT 4 sequences, the catalytic glutamic acid is replaced by alanine. Originally, the functional prediction of chitinase from class VI (lectins) was quite ambiguous [23], and more recently it was based on the similarity with the *Urtica dioica* GH19 lectin (GenBank AAA34219) characterized by two CBMs and a proline-rich hinge region [29]. However, only fifteen out of thirty-one sequences from CHIT 4 fit this definition. Moreover, a wheat chitinase (GenBank AAD28730) that belongs to CHIT 2a in our system (which corresponds to plant class IV according to [23]) was wrongly assigned to class VII, and more recently to class II [63], relying on the absence of the CBM as a diagnostic feature.

To further assess the connectedness of the GH19 sequence space, the catalytic domain of CHITs and ELYSs was investigated by sequence networks at thresholds between 60 and 90% identity. The scale-free degree distribution of the catalytic domains with *γ* = 1.1 (**S10 Fig**) is similar to previous findings for other protein families with different domain organization and sequence lengths [64]. Thus, there are approximately ten times fewer sequences with ten times more neighbors. As a consequence, a few highly connected sequences were identified (**S4 Table**). Because of their proposed high evolvability and robustness towards mutations, they might be promising starting points for directed evolution experiments [64]. The relation of cluster size distributions obtained at varying thresholds of sequence identity showed a smaller Fisher exponent than for other protein families [65] (**S8 and S9 Figs**), which means that bigger network clusters occur more frequently, and the overall connectedness of sequence space is higher. Interestingly, the cluster size distributions of ELYSs and CHITs differ at an identity threshold of 60% (**Figs 3** and **S9A**). However, due to the smaller sample size within the individual subfamilies, the Fisher exponents could not be compared between CHITs and ELYSs. We suggest that the observed discrepancy is due to a different sequence space coverage, caused either by a bias towards the study of bacteria related to human health or by ELYS sequence polymorphisms, because phages are the most abundant and diverse self-replicating entities on the planet [66].

Overall, clustering of catalytic domains by their sequence identity is in accordance with known biochemical properties of the GH19 proteins and is compatible with previous classification systems, with the advantage not to rely on other criteria such as the association with CBMs or with hinge regions, which may cause errors in classifying GH19 diversity, as also previously discussed for other GH families (1). The comprehensive collection of more than 20,000 sequences led to 51 groups, 46 of them are new and are not covered by the previous classification systems of plant chitinases, which compared in **S2 Table**. Despite the small number of 16 characterized seed sequences in only eleven of these new groups, the annotation and classification of novel GH19 genes in (meta)genomes by sequence identity with respect to our system will support the efficient selection of novel enzyme candidates that are not too close from characterized enzymes and cover new regions of the GH19 sequence space.

### Signatures of chitinase and lysozyme activity

In GH19 chitinases, the catalytic residues and the central substrate binding subsites -2, -1, +1,+2 have been reported to be important for substrate binding and therefore to be conserved [14,27,58,67,68]. We found that in total 26 residues are conserved in CHITs and in ELYSs, including the residues forming substrate binding subsites -2, -1, +1, whereas amino acids in other subsites are variable. This result can explain why several CHITs and a single ELYS (Uniprot ID: A0A7I3, see **S1 Table**) accept murein and chitin, respectively, despite the structural differences between the two substrates.

Previously, a GH19 endolysin has been shown to have structural similarity to enzymes from other lysozyme families [59]. Because the positions of the catalytic residues are similar to C-type lysozyme from GH22, it was concluded that it is an N-acetyl-β-D muramidase with a similar catalytic mechanism, despite the fact that GH22 enzymes are retaining [8]. Because of the similarity of GH19 CHITs and ELYSs, we suggest that the enzymatic mechanism is inverting in both GH19 subfamilies, despite ELYSs having a larger substrate binding cleft at subsites -4 to +3 to accommodate the bulkier murein substrate (**S17 Fig**).

**Fig 6:**
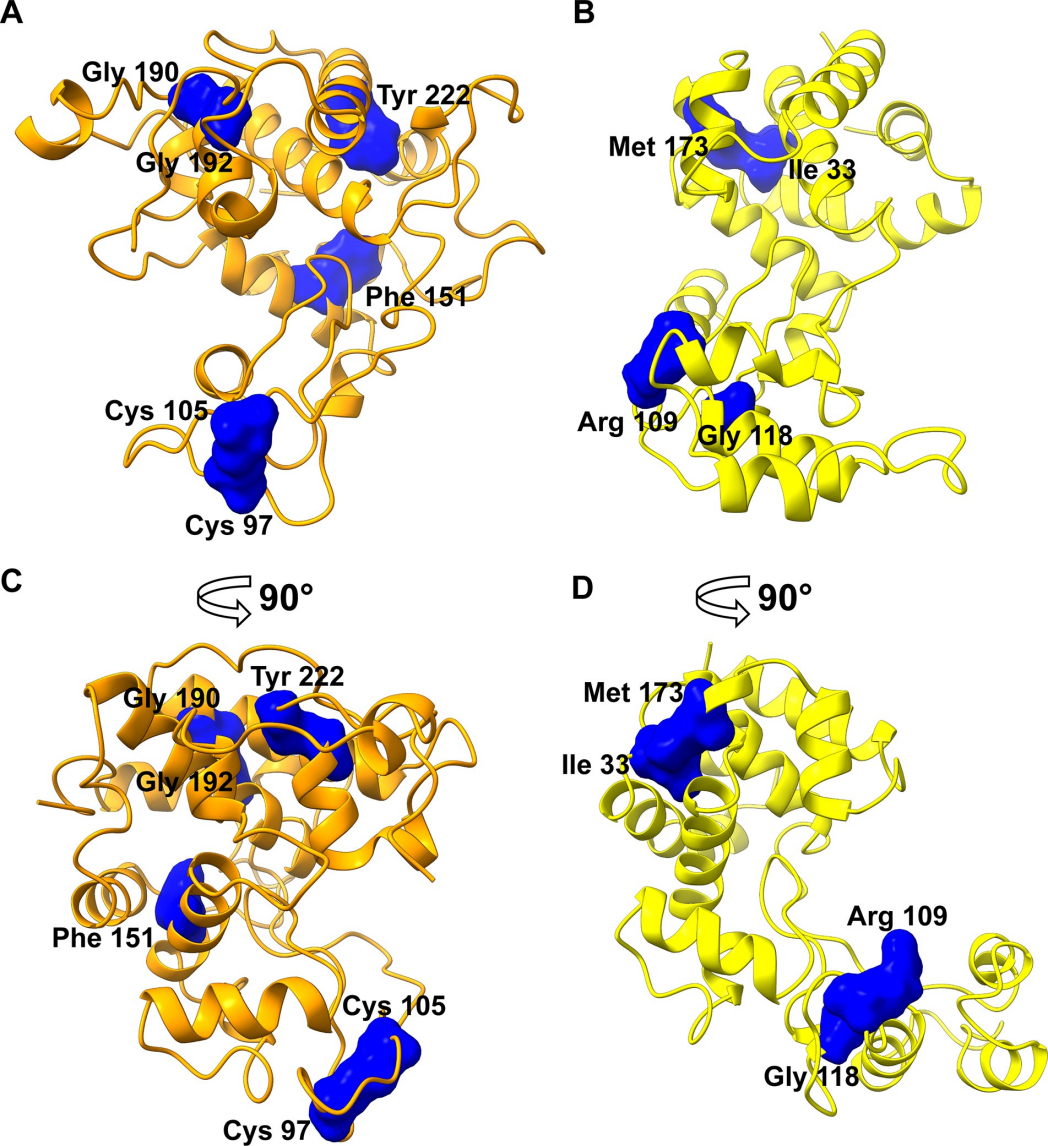
The positions of residues corresponding to subfamily-specific patterns. The residues of CHIT (**A**) and ELYS (**B**) subfamily-specific sequence patterns identified in this work are labelled and depicted as blue solvent accessible surfaces onto the reference models (PDB accessions 4j0l and 4ok7 for CHIT and ELYS subfamily, respectively), displayed in cartoon style. In (**C**) and (**D**), the same models are rotated by 90° around the vertical axis.

The residues that are conserved in CHITs or in ELYSs (**Table 2**) provide a basis for the identification of sequence patterns that might mediate substrate specificity. The sequence pattern coding for chitin hydrolysis comprised six positions (**Fig 6**), four of which are found in 62 of 63 characterized CHITs. The only outlier is a characterized fungal GH19 chitinase [69], which has low sequence similarity to the other characterized CHITs (**Fig 2**). Three positions of the four residues pattern specific for murein hydrolysis (**Fig 6**) were found in ten of the twelve characterized ELYSs. The two outliers are two endolysins from *Acinetobacter* phages, which have a low sequence similarity to the other characterized ELYSs (upper left portion of ELYS network in **Fig 2**). Moreover these two sequences, in contrast to other ELYSs, possess a unique C-terminal amphipathic helix, predicted to facilitate the permeabilization of the Gram-negative outer membrane [15,52].

The conserved CHIT sequence pattern plotted in the reference structure (Fig 6A–6C) include two cysteines forming a disulfide bridge controlling the rigidity of loop 3, a phenylalanine located next to the active site in the hinge between the two lobes, two structurally relevant glycine residues, and a tyrosine inside the superior lobe next to the catalytic cleft, with a possible role in flexibility control during the reaction. A minority of CHIT sequences especially in the homologous groups 1 and 14 lost loop 3 (**Table 3**). A previous comparative study demonstrated that an enzyme without loop 3 results in low affinity for (GlcNAc)_n_ substrates [70]. Therefore, it is probable that CHIT sequences lacking loop 3 have a lower specificity for chitin, confirming the role of the conserved cysteines. In ELYS, the conserved sequence pattern plotted on the reference ELYS structure (**Fig 6B–6D**) include a methionine and an isoleucine, which interact and stabilize the superior lobe, and an arginine and a glycine between the hinge and the PBM. Although Met173 in the sequence of the reference structure is present in only 1.9% of ELYS at standard position 173, it has similar physicochemical properties as leucine, observed in 54% of ELYSs at this standard position (**Table 2**).

The conserved glycines probably have a structural role, because they are located at the N-cap of an α-helix (Gly192 in CHITs), in a short α-helix at the core of the active site (G118 in ELYSs), or in a loop connecting two α-helices (G190 in CHITs) (**Fig 6**).

The observation that residues of the two specific sequence patterns are not in contact with the substrate suggests that they might contribute indirectly to substrate recognition by mediating conformational changes upon substrate binding, as it was observed in previous structural studies on GH19 [14,71] and other bilobal glycosidases [72]. Unfortunately, no variants at these positions have been studied yet.

It would be desirable to relate the sequences of CHITs to their substrate specificity and relative activity on various chitinous substrates by analyzing the 66 experimentally characterized CHITs (**S8 Table**). For 31 CHITs, at least two substrates with different solubility properties were tested and for 10 CHITs (underlined in **S8 Table**) the activity was determined for a broad range of substrates including insoluble and soluble chitin derivatives. However, as different protocols and different reaction conditions were used (solubility of the substrate, pH, or temperature), an association between sequence patterns and substrate specificity was not possible, yet. In general, specific activity on insoluble chitin and chitosan derivatives with a deacetylation of 70%-80% is around or more than 10-fold lower than on soluble substrates, and there is high variability in the relative efficiency of degradation of soluble high molecular weight polymers versus oligomers.

By analyzing 17 studies in which accessory CBMs were mutated, truncated, or compared with a paralogue chitinase without any CBM (bold in **S8 Table**), the role of accessory binding modules in antifungal activity was confirmed and an enhanced binding and hydrolysis of crystalline forms of polymeric chitin was supported. CBMs were also associated to allergenic properties in five sequences. Thus, the aggregated results of **S9 Table** and the extended GH19 classification are expected to support the identification of CBM-containing new candidate sequences with desired properties.

### GH19 family evolution: Early diversification, loop acquisition, and horizontal gene transfers

Representative sequences of GH19 groups defined in this study were selected for a large-scale phylogenetic study of the GH19 family (**Fig 5**). ELYSs remained confined in the genomes of phages and their bacterial hosts, where they functioned as lysozymes without detectable trend in sequence length. No structural patterns were detected to be conserved in any groups, except for a 3-helix bundle nested with the catalytic domain (corresponding to loop 3 in CHITs) which serves as a peptidoglycan binding module in the reference ELYS [59]. Because its sequence is not conserved, it might have recently evolved from an insertion under the selective pressure of co-evolutionary phage-bacteria interaction process, which is a key factor in increasing the rate of molecular evolution [73].

In contrast to ELYSs, CHITs sequences spread in both prokaryotic and eukaryotic taxa. The common ancestor probably possessed loop 3 and 4. During evolution, the CHITs increased in length by the insertion of four additional loops. Of the expected 2^6^ = 64 loop combinations, only eight were found (**Table 3**), suggesting that loop insertion or loss was not random, but followed specific evolutionary paths (**Fig 5**). This minimal "loopless"-type ancestor was maintained in the non-plant eukaryotic lineages, while at a certain point two lineages split, one of exclusively bacterial chitinases with no addition of loops, and another of plant chitinases in which loop 1 was added. The exclusively bacterial lineage further diversified in sequence and function, dividing between a lineage of *Cyanobacteria* sequences (CHIT 11-II) and the “loopless” chitinases from bacteria (CHIT 5). CHIT 5 sequences come from species processing soil organic matter, in which GH19 chitinases may have played an important role for the colonization of the ecological niche and competition with *Fungi*. Plant CHITs further diversified into a "loopless" lineage that lost loop 4, and into a "loopful" lineage by addition of loops 2, 5, and C-terminal. The two groups of catalytically inactive CHIT 3 and 4 differentiated from the "loopful" lineage and became plant stresses and growth mediators or coagulant factors in latex (**S2 Table**). Two recent HGT events involved a transfer from plants to different taxonomic groups of bacteria. In a first HGT event, the catalytic domain diversified into CHIT 6,7,8,9,10, and 12. In a second HGT event, the CHIT 11-I lineage was formed. Sequences from CHIT 11-II seem to have separated earlier with respect to other sequences from CHIT 11-I, as confirmed by the loop configurations, with CHIT 11-II sequences possessing loop 3 and some also loop 4, while most CHIT 11-I possess loops 2, 3, 4, and 5. Moreover, while CHIT 11-II sequences are present only in Cyanobacteria, mainly from *Nostocales* order and lichen forming species, the taxonomic background of CHIT 11-I is more diverse, including other *Cyanobacteria* orders, *Myxococcales* and *Ktedonobacterales*, which are bacteria that are responsible not only for the formation of lichens, but also for decomposition of biofilms on plant organic matter, thus providing a scenario in which GH19 genes may have exchanged from plants to bacteria. In all plant-derived bacterial lineages, there is a trend to lose loop 1.

In CHIT 6, loops 2 and 3 became longer: a β-N-acetylglucosaminidase activity was described for chitinases from *Vibrio proteolyticus* and *Pseudoalteromonas tunicata* [32,33], which produce (GlcNAc)_2_ from colloidal chitin hydrolysis and hydrolyze 4-nitrophenyl N-acetyl-β-D-glucosaminide, respectively. Thus, characterized CHIT 6 enzymes demonstrated to have exo-activity, whereas GH19 CHITs are typically endo-acting enzymes. Therefore, we predict that in GH19, a change of processivity might result from the acquisition of longer loops, as observed in other GH families [2].

Members of CHIT 7 have a modified N-terminal region. A biochemically characterized member of CHIT 7 has been described as an active chitinase hydrolyzing (GlcNAc)_6_ at the second bond from the non-reducing end [31], with a high free energy of binding at subsites +3 and +4 [74], whereas most of the other plant GH19 chitinases have higher affinity for binding at subsites from -3 to +3 [32]. Thus, we predict that the members of CHIT 7 preferably hydrolyze substrates at the non-reducing end. Interestingly, the same selectivity was found for some members of "loopful" chitinases, and loop 2 in the N-terminal region was suggested to mediate this function [75,76]. Therefore, we suggest that the modified N-terminal region of CHIT 7 serves a similar function. Because only three enzymes with these peculiar loop configurations have been characterized yet, their functional role is still speculative, but we predict that future experimental studies focusing on this aspect of the GH19 family, starting from sequences in CHIT 6 and 7, will provide additional insights.

A significant fraction of CHITs is linked to accessory domains. In plants, the presence of CBM18 in a fraction of groups in all main lineages can be explained by its presence in the common ancestor, while it was secondary lost or duplicated in some sequences. CBM5/12 was exchanged only between bacteria, before and after HGT of the catalytic domain from plants. A few members of the "loopless" bacterial CHIT group are linked to CBM13, which is associated with Actinobacteria xylanases and is frequently present in multi-domain enzymes [77]. Therefore, we hypothesize that CBM13 recently recombined with CHITs and putative chitinases with this domain could possess interesting and recently evolved properties. LysM, an ubiquitous non-catalytic motif repeat that was shown to bind both peptidoglycan and chitin, especially chitooligosaccharides of Nod factors in plant-bacteria symbiotic interactions [78], was found only in *Cyanobacteria* sequences from both CHIT 11-I and CHIT 11-II. Its association with phylogenetically distinct catalytic domains and its absence from any other plant GH19 chitinase suggest that this accessory domain, like CBM5/12, was horizontally transferred among bacteria. Considering that LysM is found in 75% of *Cyanobacteria* CHIT 11-II sequences and only in three CHIT11-I sequences from different *Cyanobacteria* taxa, but not from other bacterial taxa in the same sub-cluster, we hypothesize that LysM first associated to CHIT11-II catalytic domain and later recombined with a paralogue gene, belonging to CHIT11-I in the same *Cyanobacteria* organism. Considering the role of LysM motif, GH19 in these *Cyanobacteria* species could have an essential role in modulating symbiotic associations with fungal or other bacterial species.

### Conclusion and outlook

In this study, we applied a bioinformatics workflow to retrieve and analyze the sequence space and evolution of more than 20,000 sequences that contained a GH19 domain. These sequences were organized in sequence networks (https://doi.org/10.18419/darus-802) and subfamilies that correlate with the chitinolytic and lysozyme activities detected experimentally. Moreover, the two GH19 subfamilies (ELYS and CHIT) were further analyzed by sub-networks, providing new groups that extend the previous systems of classification. New profile HMMs (https://doi.org/10.18419/darus-803) were obtained to provide standard numbering schemes for annotating new (meta)genomic sequences and for distinguishing the features of CHITs from ELYSs. This permitted to identify specific sequence patterns coding for chitin and murein hydrolysis, thus providing a molecular hypothesis for the substrate specificity of GH19 enzymes promiscuity and guiding rational-based design of mutations. A binary loop code for a simplified description of GH19 chitinase loops was developed: combining it with the study of GH19 evolution at a large-scale allowed us to trace loop evolutionary paths.

Using centroid sequences for a phylogenetic analysis provided a comprehensive view on GH19 evolution, including association to different accessory domains. Previous GH19 evolutionary reconstructions overrepresented plant sequences [28,79], whereas our analysis permitted to focus also on bacterial GH19, which represent most of the GH19 sequences, as suggested in a recent review [6]. As a consequence, our work indicates that *Actinobacteria* GH19 chitinases might not have been derived from plant chitinases, as hypothesized previously [26,79], and that much more diverse bacterial taxa than previously thought might possess GH19 chitinases.

All sequences and structures are publicly available at https://gh19ed.biocatnet.de to support the search for novel biotechnologically interesting GH19 candidate enzymes in the (meta)genomes of neglected taxonomic groups or in regions of the sequence space (highlighted in this study) in which sequences with peculiar domain or loop compositions can be found, and few to no sequences have been described, yet.

## Methods

### GH19ED database setup

In order to select the sequences belonging to the GH19 family and create the GH19ED, BLAST [80] searches were performed using the GH19 domains of a list of seed sequences as queries against the NCBI non-redundant protein database (https://www.ncbi.nlm.nih.gov/refseq/about/nonredundantproteins/) [81] and the Protein Data Bank (PDB, https://www.rcsb.org/) [82] with a maximal E-value of 10^−10^. The seed sequences were obtained by downloading the GH19 sequences stored in CAZy (http://www.cazy.org/GH19.html, accessed on 01/09/2019) under “Structure” and “Characterized” tabs, if experimental literature to confirm their activity was found. A manual search was made on individual CAZy entries and by screening the results in Google Scholar search engine with the keywords “glycoside hydrolase 19”, “chitinase”, “lysozyme”, or “endolysin”. Sequences longer than 120 amino acids were retrieved and inserted together with their name and source organism into the GH19ED (https://gh19ed.biocatnet.de/) within the BioCatNet database system [56]. A global sequence identity threshold of 99% was applied to assign individual sequence entries to *protein* entries in the database. The GH19 profile HMM PF00182 from Pfam [83] was used for scanning the sequences contained in the GH19ED using the HMMER software suite (Version 3.1b2) [84], to annotate the GH19 catalytic domains. Sequences with no hit were removed from the database. The parameters used for annotations were a maximal E-value of 10^−5^, a minimum hit length of 120 amino acids, and a bias ratio (HMMER bias/HMMER profile-sequence alignment score) < 0.1. The latter criterion was chosen to reduce false positives due to regions of low complexity.

### Protein sequence networks

The sequences of all the annotated GH19 domains were clustered by 90% global identity using the heuristic clustering algorithm of USEARCH v11.0.667 [85]. The pairwise sequence identities between these representative domain-level sequences (centroids) were calculated by pairwise Needleman-Wunsch sequence alignments as implemented in the EMBOSS software suite version 6.6.0 [86], using the BLOSUM62 scoring matrix [87] with 10 and 0.5 as gap opening and gap extension penalties, respectively. In contrast to the approach using BLAST for sequence similarity networks [88], we use Needleman-Wunsch alignments and thus refer to “protein sequence networks” instead. The GNU parallel package [89] was used to reduce the computational time for pairwise alignments by multithreading. Sequence networks were generated to visualize the centroids (i.e. the representative sequences form clustering) as nodes that are connected with edges (i.e. links). A pair of nodes was connected by an edge, if its edge weight (i.e. the value of sequence identity) exceeded a given threshold. Networks were exported in GraphML format by NetworkX version 1.9 [90] and visualized with Cytoscape 3.7.2 [91] using the prefuse force-directed OpenCL layout algorithm with respect to the edge weights, thus sequence pairs with higher sequence identity were placed in closer proximity.

### Distributions derived from protein sequence networks

The distributions of the number of direct neighbours for each sequence (degree, *n*) and of the number of sequences forming connected networks (cluster size, *s*) were derived from protein sequence networks at different thresholds of pairwise sequence identity. The number of nodes *N(n)* having a degree of *n* was fitted by a power law *N(n) ~ n*^*-γ*^, and the scaling exponent *γ* was derived from a log-log plot [64]. The number of clusters *N(s)* with size *s* was fitted by a power law *N(s) ~ s*^*-τ*^, and the Fisher exponent τ was derived from a log-log plot, too [65]. Logarithmic histograms for the cluster sizes *s* were obtained for subsequent intervals (2 ≤ *s* ≤ 10, 11 ≤ *s* ≤ 100, 101 ≤ *s* ≤1000, and 1001 ≤ *s* ≤ 10,000). The slopes *τ*_*h*_ of these histograms were determined for sequence networks at different thresholds of sequence identity. The Fisher exponent *τ* was derived by fitting *τ*_*h*_ against model distributions as described previously [65].

The distributions of degrees and cluster sizes were analysed by linear fitting using the fitlm function from the Statistics and Machine Learning Toolbox (version 11.7) in MATLAB (version R2020a, The MathWorks, Natick, MA, USA).

### Subfamily assignment and standard numbering scheme

Networks formed at a sequence identity threshold of 40% and containing at least one characterized seed sequence were analyzed. The sequences of centroids and from the same cluster (see *Protein sequence networks* above) were assigned to one subfamily, labelled “superfamily” on the website of the GH19ED by BioCatNet’s default nomenclature. For each subfamily that contained at least one protein with crystal structure information, a standard numbering scheme was established [57]. A protein with PDB structure information was chosen as reference, and a profile HMM was derived from a multiple sequence alignment between all members of the subfamily. If the subfamily included more than one protein with PDB structure, these sequences were aligned by a structure-based alignment using the *mmaker* command implemented in ChimeraX 0.9 (RBVI, University of California, San Francisco, CA, USA, [92]. The other seed sequences in the same subfamily were then aligned to the fixed structural alignment using the “—add” flag option available in MAFFT 7.407 [93], described at https://mafft.cbrc.jp/alignment/server/add.html. If the subfamily contained only one protein with PDB structure, a sequence-based alignment with other seeds was created using the MAFFT “L-INS-i” strategy [94], improved by adding information of up to 600 close homologs obtained from a search in Uniprot non-redundant Uniref50 database (ftp://ftp.uniprot.org/pub/databases/uniprot/uniref/uniref50) and by using a restrictive E-value threshold of 10^−20^ (a procedure described in more detail at https://mafft.cbrc.jp/alignment/software/algorithms/algorithms.html#homologs). The obtained alignments of the seed sequences were manually cut with respect to the length of the GH19 domain of the reference structures and used to generate subfamily-specific profile HMMs. Each sequence was aligned to the subfamily-specific profile HMM and standard positions annotated by using the alignment with the reference sequence, i.e. PDB accession 4j0l of “loopful” plant chitinase from rye seed *Secale cereale* for the CHIT subfamily, and PDB accssion 4ok7 of bacteriophage SPN1S endolysin from *Salmonella typhimurium* for the ELYS subfamily. In this way, a subfamily-specific standard numbering scheme was created. Literature on experimentally characterized enzymes with known sequence was employed to annotate known functions at defined standard positions.

### Group assignment

The standard numbering schemes were used to re-annotate the GH19 domains in all sequences of each subfamily (or “superfamily”). The sequences of the GH19 domains were retrieved and aligned to calculate pairwise sequence identities and to construct networks of the centroid sequences. A 60% identity threshold was used to split each subfamily into clusters (sub-networks or subgraphs). Each cluster containing at least one seed sequence or formed by at least ten centroid sequences was called a group, labelled “homologous family” on the website of the GH19ED by BioCatNet’s default nomenclature.

### Conservation analysis

For each subfamily with a standard numbering scheme, a conservation analysis was performed. The sequences of the GH19 domains were clustered in descending length order with USEARCH, and a 65% identity threshold was applied to identify less than 300 representative centroids for generating a multiple sequence alignment with the E-ins-I algorithm of MAFFT [94]. The relative evolutionary rate at each site was evaluated by Rate4Site (Version 2.01) [95] by employing an LG substitution rate matrix [96] and an empirical Bayesian approach. Five evolutionary rate categories were defined: from the “fastest” (assigned to conservation score 1) to the “slowest” (assigned to conservation score 5). If less than half of the sequences in the alignment contained gaps at a specific site, rate 1 was assigned. Thus, each standard position was annotated by an evolutionary rate between 1 and 5. The reference sequences of each subfamily were structurally aligned. The most conserved positions (conservation score 5) without a gap in at least 90% of each subfamily sequences were identified and considered to be shared between the subfamilies, if structurally aligned in the references and there was at least an amino acid in common in more than 5% of the sequences. In contrast, the most conserved standard positions that could not be structurally aligned between the reference sequences of the subfamilies or were aligned but overlapped for less than 5% in the amino acid frequency distribution were considered specifically conserved within each subfamily. Thus, a pattern of residue distributions at specifically conserved positions was obtained. The 5% threshold was set to avoid the identification of patterns that are not subfamily-specific. All figures were prepared with ChimeraX 0.9.

### Annotations and phylogenetic analysis of GH19

Seven different accessory domains that are usually associated with chitinases and lysozymes were annotated in the GH19ED: CBM18, CBM5/12 and CBM13, LysM, PG_binding_1, PGRP and SH3_3. For each accessory domain, a profile HMM was built using HMMER from the multiple sequence alignments available in the SMART database [97] with accession codes SM00270 (CBM18), SM00495 (CBM5/12), SM00458 (CBM13), SM00257 (LysM) and SM00701 (PGRP). The profile HMMs available in Pfam with the accession codes PF01471 and PF08239 were used for PG_binding_1 and SH3_3, respectively. Each sequence in the GH19ED was scanned with the seven profile HMMs using as thresholds an E-value of 10^−5^, a minimum length of 20 residues, and a bias ratio of 1.

In order to annotate the 3-helix peptidoglycan binding bundle (PBM), which has been reported for the endolysin from bacteriophage SPN1S [59], a sequence-based alignment of 600 close homologs was performed by MAFFT “L-INS-i” strategy [94]. The homologs were obtained by a BLAST search in the Uniprot non-redundant Uniref50 database, using as query the reference endolysin and an E-value of 10^−20^. The alignment was manually cut with respect to the length of the reference PBM. A profile HMM was derived and used for annotation of the GH19ED sequences.

The standard positions corresponding to the six chitinase loops were annotated by comparisons to recent GH19 literature with respect to the corresponding motifs present in the reference “loopful” plant chitinase from rye seed (PDB accession 4j0l) and absent in the “loopless” plant chitinase from *Gemmabryum coronatum* (PDB accession 3wh1), as shown in **S13A Fig**. The minimum length allowed for a loop was four residues shorter with respect to the loop length of the reference.

A large-scale phylogeny was built from the GH19 domain centroids of all groups. The centroids were defined by using CD-HIT [98] at 40% identity threshold and word size 2. The centroids were aligned by using the E-ins-I algorithm of MAFFT. A Bio-Neighbour Joining [99] starting tree was generated from this alignment through phylogeny.fr web service (http://www.phylogeny.fr/). These results were refined in a Bayesian analysis by Bali-Phy 3.4.1 [100]. Six independent Monte Carlo Markov chain analyses were performed until convergence and good mixing were obtained (http://www.bali-phy.org/README.html#mixing_and_convergence). The first 50% of samples were discarded to eliminate the background noise at the beginning of the run. Each analysis was performed at default parameters priors with an LG empirical substitution rate matrix and an rs07 [101] insertion/deletion model. The resulting unrooted tree is the majority consensus from all the samples collected during the runs. The position of the root was obtained by considering the splitting between subfamily networks, if supported by posterior probability in the obtained phylogeny.

## Supporting information

S1 Fig The single displacement hydrolysis mechanism of GH19 [46]. One acidic, one basic glutamate and a serine (or threonine) for water placement are generally required in the active site and the hydrolysis product has inversion of the anomeric configuration from α to β.
(PDF)

S2 Fig Length distribution histogram of sequence entries in the GH19ED database, with a bin size of 20 residues. The two main peaks are around 200 and 580 residues. Only few sequences are longer than 1100 residues (up to 6000 residues).
(PDF)

S3 Fig Histograms of pairwise identities for the catalytic domains of chitinases (CHIT, upper panel) and endolysins (ELYS, lower panel) from the GH19ED. (PDF)

S4 Fig Protein sequence networks of all GH19 representative domains (5229 centroid sequences obtained from clustering at 90% identity) connected by edges with an identity cut-off of 40%. The two bigger clusters contain seed sequences of characterized endolysins (2738 sequence nodes on the left) and chitinases (2329 sequence nodes on the right). The prefuse force-directed OpenCL layout with respect to the edge weights was used. The domains were extracted from Pfam’s GH19 profile HMM (PF00182) by scanning the sequences collected through BLAST searches, in which the seed sequences reported in **S1 Table** were used as queries. Nodes are colored according to their annotated taxonomic source. In **Fig 2** only the two main clusters are visualized.
(PDF)

S5 Fig Length distribution histograms of ELYS and CHIT domains in the GH19ED database, with a bin size of 5 residues. The two main peaks are around 175 and 200 for ELYSs, 200 and 245 for CHITs.
(PDF)

S6 Fig Accessory binding modules plotted with different colors onto sequence networks for CHIT groups. The two black arrows indicate the centroids from bacteria and *Metazoa* possessing a CBM18 (typical of plants) and a CBM5/12 (typical of bacteria), respectively. It is likely that for these sequences both the CBM and the catalytic domain were transferred to these organisms from plants and bacteria. The group identifiers are the same as in **Fig 3A**.
(PDF)

S7 Fig Accessory binding modules plotted with different colors onto ELYS groups sequence networks. The group identifiers are the same as in **Fig 3B**.
(PDF)

S8 Fig The degree distribution *N(n)* for the catalytic domains from the GH19ED at a threshold of 95% sequence identity was approximated by a power-law for degrees ≤ 50 (red line) yielding a scaling exponent of *γ* = 1.1. (PDF)

S9 Fig Histograms of the cluster size distributions *N(s)* for the catalytic domains from the GH19ED at thresholds of 60% (**A**), 70% (**B**), 80% (**C**), and 90% (**D**) sequence identity. The distributions for all annotated catalytic domains (depicted in black) were approximated by a power law yielding exponents τh of 0.7, 0.7, 0.8 and 1.1, respectively (compare with **S10 Fig**). The histogram data for the catalytic domains in the individual CHIT and ELYS subfamilies are depicted as red and blue triangles, respectively.
(PDF)

S10 Fig Linear fitting of the slopes of the histograms (S9 Fig), used to linearly extrapolate the theoretical exponent τ for individual amino acid exchanges at 100% sequence identity. (PDF)

S11 Fig Rate4Site conservation scores (see *Methods* section of the main text) are visualized onto models of CHIT reference (**A-C**, PDB accession 4j0l) and ELYS reference structure (**B-D**, PDB accession 4ok7). (**A**) and (**B**) models are visualized as cartoon with α-helices shown as cylinders, substrate binding residues as sticks (except glycine), and catalytic residues as balls and sticks. (**C**) and (**D**) are the same models shown in **A** and **B**, represented as solvent accessible surface areas.
(PDF)

S12 Fig Rate4Site conservation score 1 (least conserved) and 5 (most conserved), as declared in the *Methods* section of the main text, are visualized with two different colors (red for score 1 and blue for score 5) plotted onto 3D models of rye seed CHIT reference (**A-C** for score 5 and **B-D** for score 1, PDB accession 4jol) and ELYS reference from bacteriophage SPN1S (**E-G** for score 5 and **F-H** for score 1, PDB accession 4ok7). (**A-B**) The CHIT reference model is visualized as cartoon, with substrate binding residues labelled in **S11A Fig** as sticks (except for glycine). (**C-D**) The ELYS reference model is visualized in cartoon with residues as sticks if corresponding to CHIT substrate binding residues reported in **Table 1**. (**E-F-G-H**) The same models presented above, shown as solvent accessible surface areas.
(PDF)

S13 Fig (**A**) The structures of GH19 “loopful” chitinase from rye seed *Secale cereale* (orange, PDB accession 4jol) and “loopless” chitinase from moss *Gemmabryum coronatum* (cyan, PDB accession 3wh1) superposed with the *mmaker* command implemented in ChimeraX 0.9, showing in red the five additional loops of “loopful” plant chitinases and the shared loop 3. The two tetra-chitooligosaccharides spanning the catalytic cleft in complex with the crystal structure of rye seed are shown; numbers under sugar moieties are in accordance with the standard nomenclature for GH. Cleavage occurs between units bound in subsites -1 and +1 [140]. (**B**) The structure of GH19 endolysin from bacteriophage SPN1S (PDB code 4ok7) of *Salmonella typhimurium* is shown for comparison.
(PDF)

S14 Fig Length distribution of CHIT loop motifs. The black arrow indicates the minimum length threshold used to define the presence of a loop, as specified in the *Methods* section. The red arrow indicates the threshold used to separate the two modes of length observed for loops 2 and 3.
(PDF)

S15 Fig Protein sequence networks of representative domains of CHITs in Fig 3A in which the centroids used for the phylogenetic analysis reported in Fig 5 are marked. Different colors are used to indicate different clusters obtained by the CD-HIT clustering analysis, if more than one cluster is present in each group. A legend is provided with a Roman numeral code corresponding to the group sub-cluster reported on sequence headers in **Fig 5**.
(PDF)

S16 Fig Protein sequence networks of representative domains of ELYSs in Fig 3B in which the centroids used for the phylogenetic analysis reported in Fig 5 are marked. Different colors are used to indicate different clusters obtained by the CD-HIT clustering analysis, if more than one cluster is present in each group. A legend is provided with a Roman numeral code corresponding to the group sub-cluster reported on sequence headers in **Fig 5**.
(PDF)

S17 Fig (**A**) The rye seed chitinase model (PDB accession 4j0l) is visualized in blue transparent solvent accessible surface area (loops 1, 2, 5 and C-terminal are colored in red), superposed to the endolysin from bacteriophage SPN1S model (PDB accession 4ok7), visualized in yellow solvent accessible surface area; two co-crystallized (GlcNAc)_4-6_ are in the catalytic cleft [25]. (**B**) The same object is rotated by 90° around the vertical axis. Black arrows highlight the regions in which the cleft of the chitinase model is tighter than the one of the endolysin.
(PDF)

S1 Table List of GH19 seed sequences, used for BLAST searches to initialize the GH19ED database. These sequences were manually screened from literature starting from the entries reported in the “Characterized” and “Structure” tabs of the GH19 CAZy page (http://www.cazy.org/GH19.html). Sequences retrieved only from literature are listed in bold. Superfamily assignments are based on **Fig 2**. Subfamily group assignments and numeral identifiers are based on **Fig 3A and 3B**. hfam ID = group identifier (homologous family in GH19ED database). CBM = Carbohydrate binding module.
(PDF)

S2 Table List of GH19 subfamilies and groups defined in this study (Figs 2, 3A and 3B), their respective number of sequences and proteins (99% identity clustering of sequences), and the average number of residues in the catalytic domain ± standard deviation. h-fam ID = group identifier (homologous family in GH19ED database) based on **Fig 3A and 3B**.
(PDF)

S3 Table List of catalytically inactive chitinase-like GH19 proteins (CLP) from CHIT groups 3 and 4, shown also in Fig 3. hfam ID = group identifier (homologous family in GH19ED database).
(PDF)

S4 Table Sequence entries from the GH19ED with degree greater than 300 (i.e. more than 300 neighboring sequences) in hub regions of the catalytic domains (at a threshold of 95% sequence identity) are listed with their corresponding annotation, taxonomic name of the source organism, and NCBI accession (compare with S7 Fig). hfam ID = group identifier (homologous family in the GH19ED) based on **Fig 3A and 3B**.
(PDF)

S5 Table Sites with conservation score 5 (see *Methods* section in the main text) in the CHIT subfamily. Standard position numbering is according to the chitinase from rye seed (PDB accession 4j0l). Information is provided about the frequency of amino acids (if higher than 1%, up to the forth residue in descending order of frequency) at each site, and the respective function, if known from [25]. Standard positions corresponding to conserved sites in the ELYS subfamily (**S6 Table**) are highlighted in bold. Standard positions of the sequence pattern specific for CHITs are marked in red.
(PDF)

S6 Table Sites with conservation score 5 (see *Methods* section in the main text) in the ELYS subfamily. Standard position numbering is according to the endolysin from bacteriophage SPN1S of *Salmonella typhimurium* (PDB accession 4ok7). Information is provided about the frequency of amino acids (if higher than 1%, up to the forth residue in descending order of frequency) at each site, and the respective function, if known from [118]. Standard positions corresponding to conserved sites in the CHIT subfamily (**S5 Table**) are highlighted in bold. Standard positions of the sequence pattern specific for ELYSs are marked in red.
(PDF)

S7 Table Loop conservation scores at CHIT standard positions. The “loopful” plant chitinase from rye seed (PDB accession 4j0l) is taken as reference. Conservation score ranges from 1 (least conserved) to 5 (most conserved). Substrate binding residues that are present in the reference chitinase are highlighted in bold.
(PDF)

S8 Table List of the substrates in which GH19 CHIT seed sequences are active with notes on their activity. Subfamily groups assignment and numeral identifiers are based on **Fig 3A and 3B**. The sequences in which the effect of CBMs on catalytic and antifungal activity was tested are highlighted in bold. The Uniprot accession of sequences in which the activity was tested on insoluble chitin, on soluble chitin polymers or oligomers (derivatives comprised), and on chitosan are underlined and the references for the protocols used are reported in the notes. hfam ID = group identifier (homologous family in GH19ED database).
(PDF)

S9 Table List of referenced CBM properties demonstrated by point mutation, by truncation variants, or by comparing two similar enzymes from the same organism, with and without the CBM. (PDF)
