# Supplementary material for: The GH19 Engineering Database: Sequence diversity, substrate scope, and evolution in glycoside hydrolase family 19
Source: PLoS One. 2021 Oct 26;16(10):e0256817. doi: 10.1371/journal.pone.0256817 (PMC8547705; doi:10.1371/journal.pone.0256817)
Supplement: S1 Fig — One acidic, one basic glutamate and a serine (or threonine) for water placement are generally required in the active site and the hydrolysis product has inversion of the anomeric configuration from α to β. (PDF) [file pone.0256817.s001.pdf]

Figures

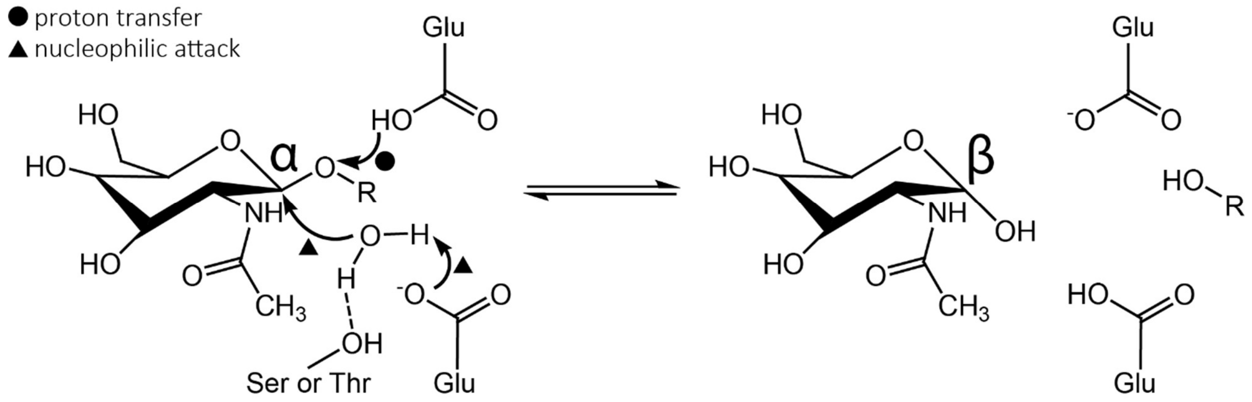

Figure S1. The single displacement hydrolysis mechanism of GH19 [46]. One acidic, one basic glutamate and a serine (or threonine) for water placement are generally required in the active site and the hydrolysis product has inversion of the anomeric configuration from α to β.
