# Supplementary material for: The GH19 Engineering Database: Sequence diversity, substrate scope, and evolution in glycoside hydrolase family 19
Source: PLoS One. 2021 Oct 26;16(10):e0256817. doi: 10.1371/journal.pone.0256817 (PMC8547705; doi:10.1371/journal.pone.0256817)
Supplement: S2 Fig — The two main peaks are around 200 and 580 residues. Only few sequences are longer than 1100 residues (up to 6000 residues). (PDF) [file pone.0256817.s002.pdf]

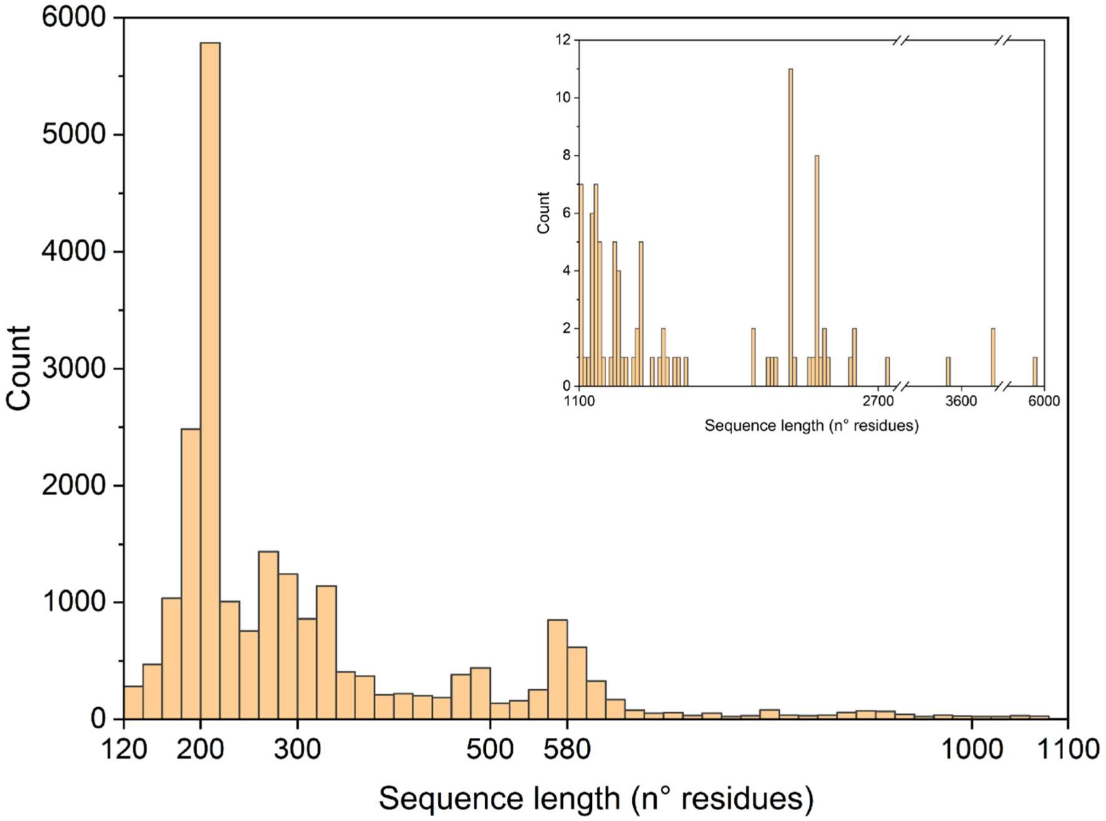

Figure S2. Length distribution histogram of sequence entries in the GH19ED database, with a bin size of 20 residues. The two main peaks are around 200 and 580 residues. Only few sequences are longer than 1100 residues (up to 6000 residues).
