# Supplementary material for: The GH19 Engineering Database: Sequence diversity, substrate scope, and evolution in glycoside hydrolase family 19
Source: PLoS One. 2021 Oct 26;16(10):e0256817. doi: 10.1371/journal.pone.0256817 (PMC8547705; doi:10.1371/journal.pone.0256817)
Supplement: S3 Fig — (PDF) [file pone.0256817.s003.pdf]

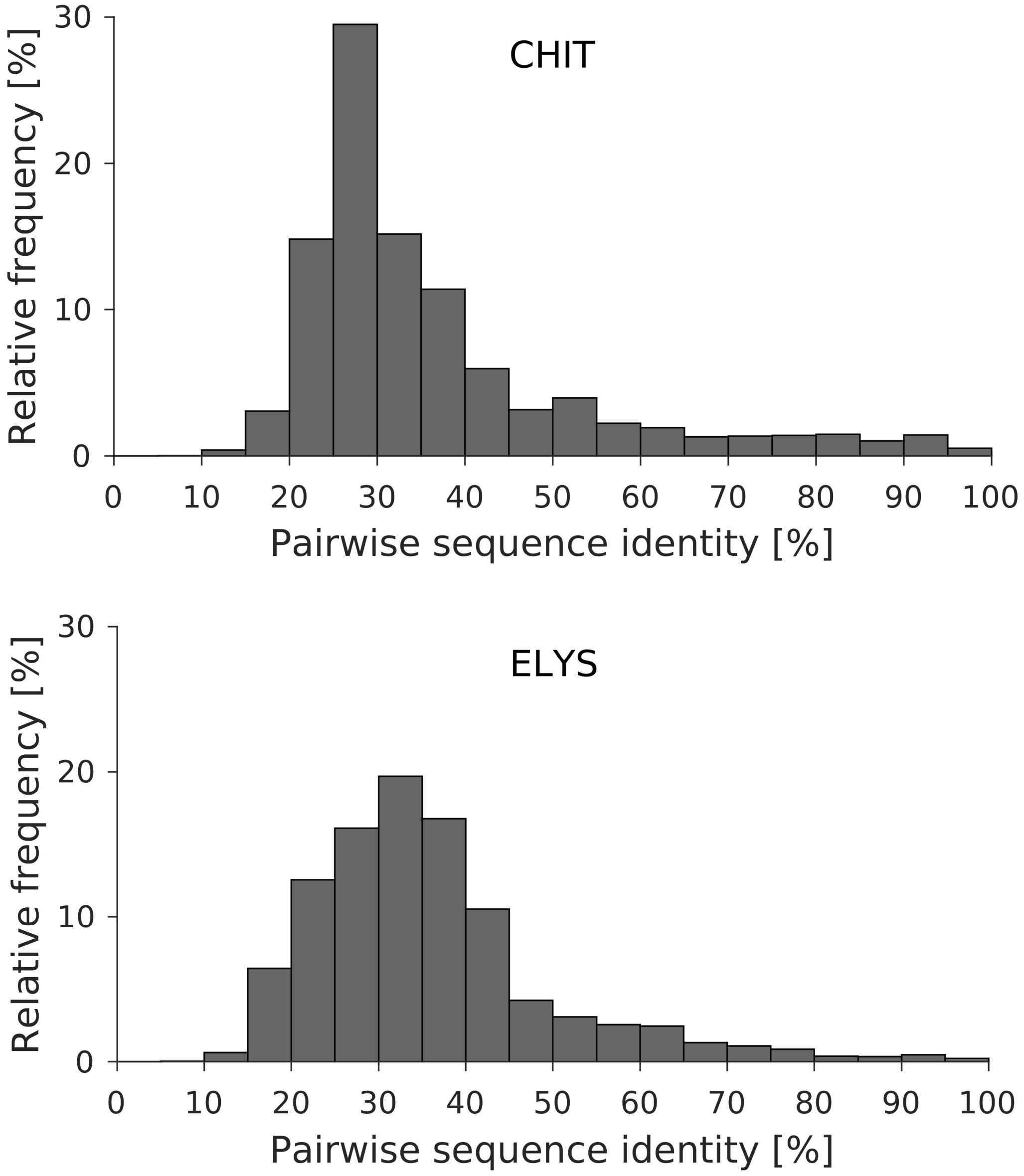

Figure S3. Histograms of pairwise identities for the catalytic domains of chitinases (CHIT, upper panel) and endolysins (ELYS, lower panel) from the GH19ED.
