# Supplementary material for: The GH19 Engineering Database: Sequence diversity, substrate scope, and evolution in glycoside hydrolase family 19
Source: PLoS One. 2021 Oct 26;16(10):e0256817. doi: 10.1371/journal.pone.0256817 (PMC8547705; doi:10.1371/journal.pone.0256817)
Supplement: S4 Fig — The two bigger clusters contain seed sequences of characterized endolysins (2738 sequence nodes on the left) and chitinases (2329 sequence nodes on the right). The prefuse force-directed OpenCL layout with respect to the edge weights was used. The domains were extracted from Pfam’s GH19 profile HMM (PF00182) by scanning the sequences collected through BLAST searches, in which the seed sequences reported in S1 Table were used as queries. Nodes are colored according to their annotated taxonomic source. In Fig 2 only the two main clusters are visualized. (PDF) [file pone.0256817.s004.pdf]

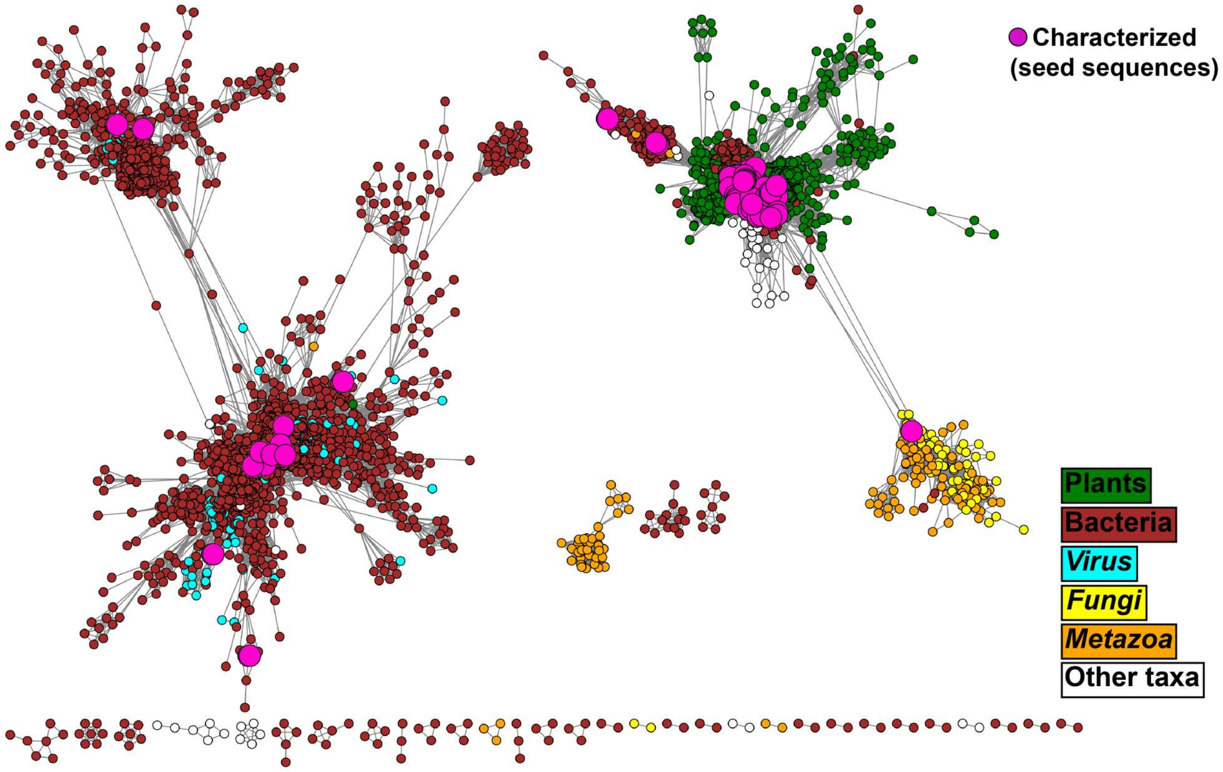

Figure S4. Protein sequence networks of all GH19 representative domains (5229 centroid sequences obtained from clustering at 90% identity) connected by edges with an identity cut-off of 40%. The two bigger clusters contain seed sequences of characterized endolysins (2738 sequence nodes on the left) and chitinases (2329 sequence nodes on the right). The prefuse force-directed OpenCL layout with respect to the edge weights was used. The domains were extracted from Pfam's GH19 profile HMM (PF00182) by scanning the sequences collected through BLAST searches, in which the seed sequences reported in **Tab. S1** were used as queries. Nodes are colored according to their annotated taxonomic source. In **Fig. 2** only the two main clusters are visualized.
