# Supplementary material for: The GH19 Engineering Database: Sequence diversity, substrate scope, and evolution in glycoside hydrolase family 19
Source: PLoS One. 2021 Oct 26;16(10):e0256817. doi: 10.1371/journal.pone.0256817 (PMC8547705; doi:10.1371/journal.pone.0256817)
Supplement: S5 Fig — The two main peaks are around 175 and 200 for ELYSs, 200 and 245 for CHITs. (PDF) [file pone.0256817.s005.pdf]

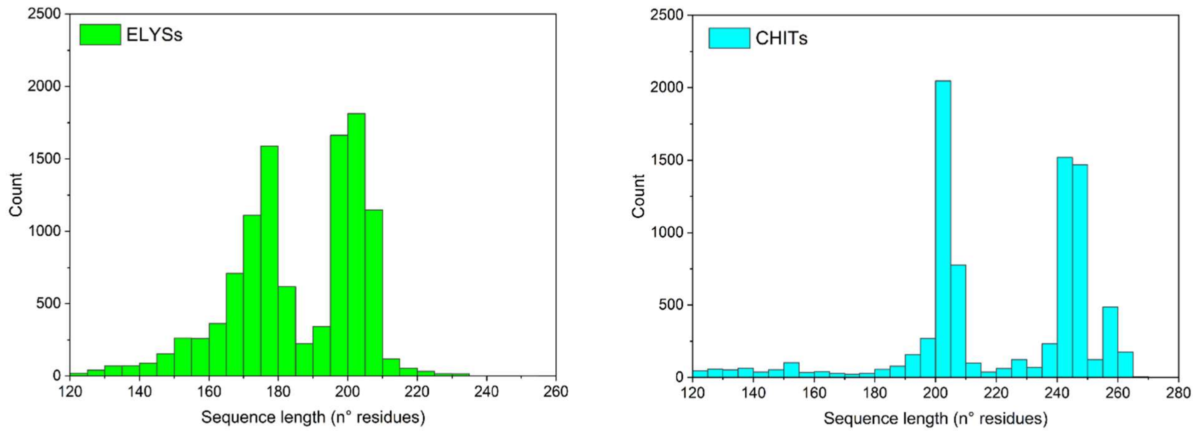

Figure S5. Length distribution histograms of ELYS and CHIT domains in the GH19ED database, with a bin size of 5 residues. The two main peaks are around 175 and 200 for ELYSs, 200 and 245 for CHITs.
