# Supplementary material for: The GH19 Engineering Database: Sequence diversity, substrate scope, and evolution in glycoside hydrolase family 19
Source: PLoS One. 2021 Oct 26;16(10):e0256817. doi: 10.1371/journal.pone.0256817 (PMC8547705; doi:10.1371/journal.pone.0256817)
Supplement: S6 Fig — The two black arrows indicate the centroids from bacteria and Metazoa possessing a CBM18 (typical of plants) and a CBM5/12 (typical of bacteria), respectively. It is likely that for these sequences both the CBM and the catalytic domain were transferred to these organisms from plants and bacteria. The group identifiers are the same as in Fig 3A. (PDF) [file pone.0256817.s006.pdf]

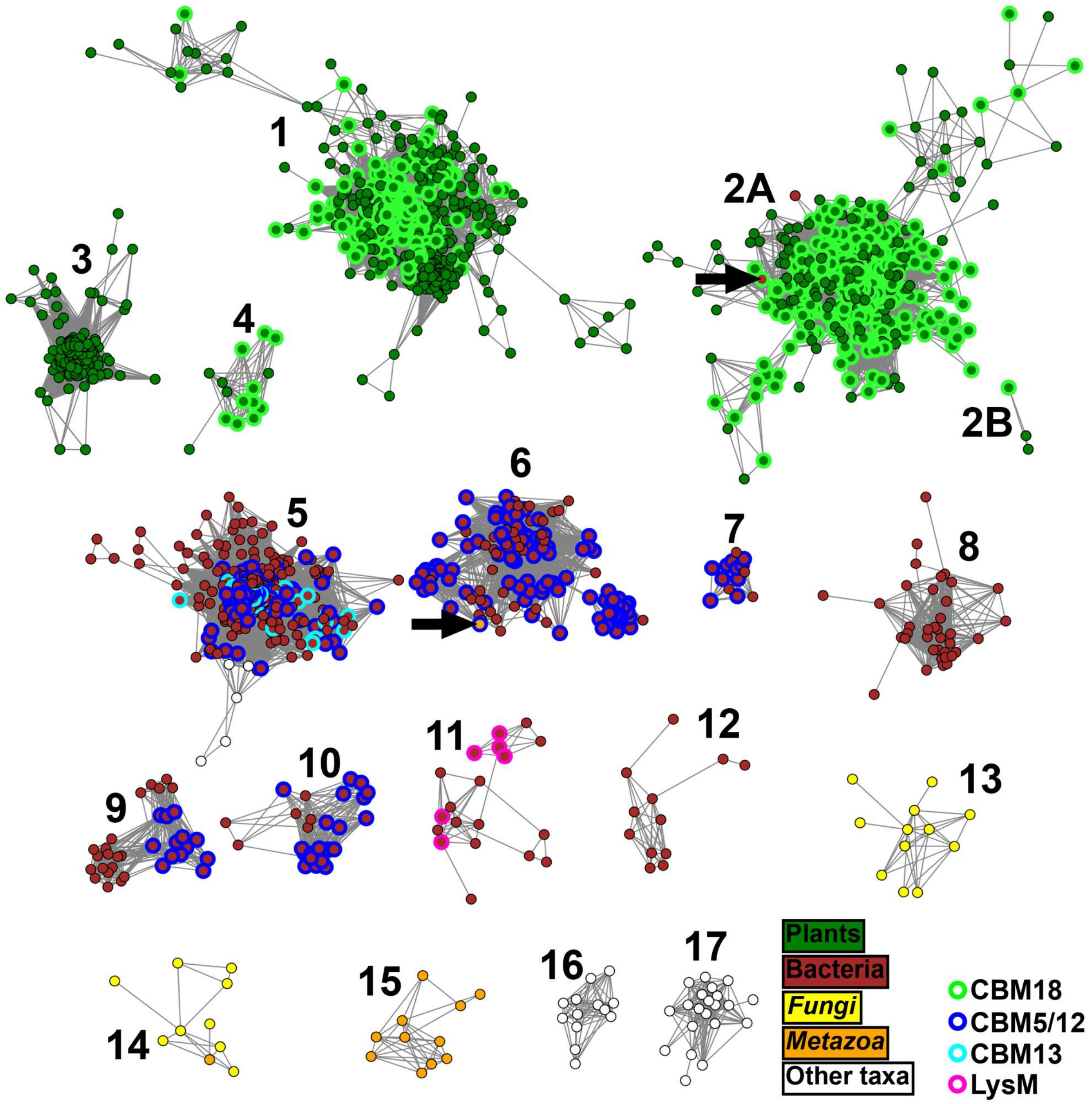

Figure S6. Accessory binding modules plotted with different colors onto sequence networks for CHIT groups. The two black arrows indicate the centroids from bacteria and *Metazoa* possessing a CBM18 (typical of plants) and a CBM5/12 (typical of bacteria), respectively. It is likely that for these sequences both the CBM and the catalytic domain were transferred to these organisms from plants and bacteria. The group identifiers are the same as in **Fig. 3A**.
