# Supplementary material for: The GH19 Engineering Database: Sequence diversity, substrate scope, and evolution in glycoside hydrolase family 19
Source: PLoS One. 2021 Oct 26;16(10):e0256817. doi: 10.1371/journal.pone.0256817 (PMC8547705; doi:10.1371/journal.pone.0256817)
Supplement: S7 Fig — The group identifiers are the same as in Fig 3B. (PDF) [file pone.0256817.s007.pdf]

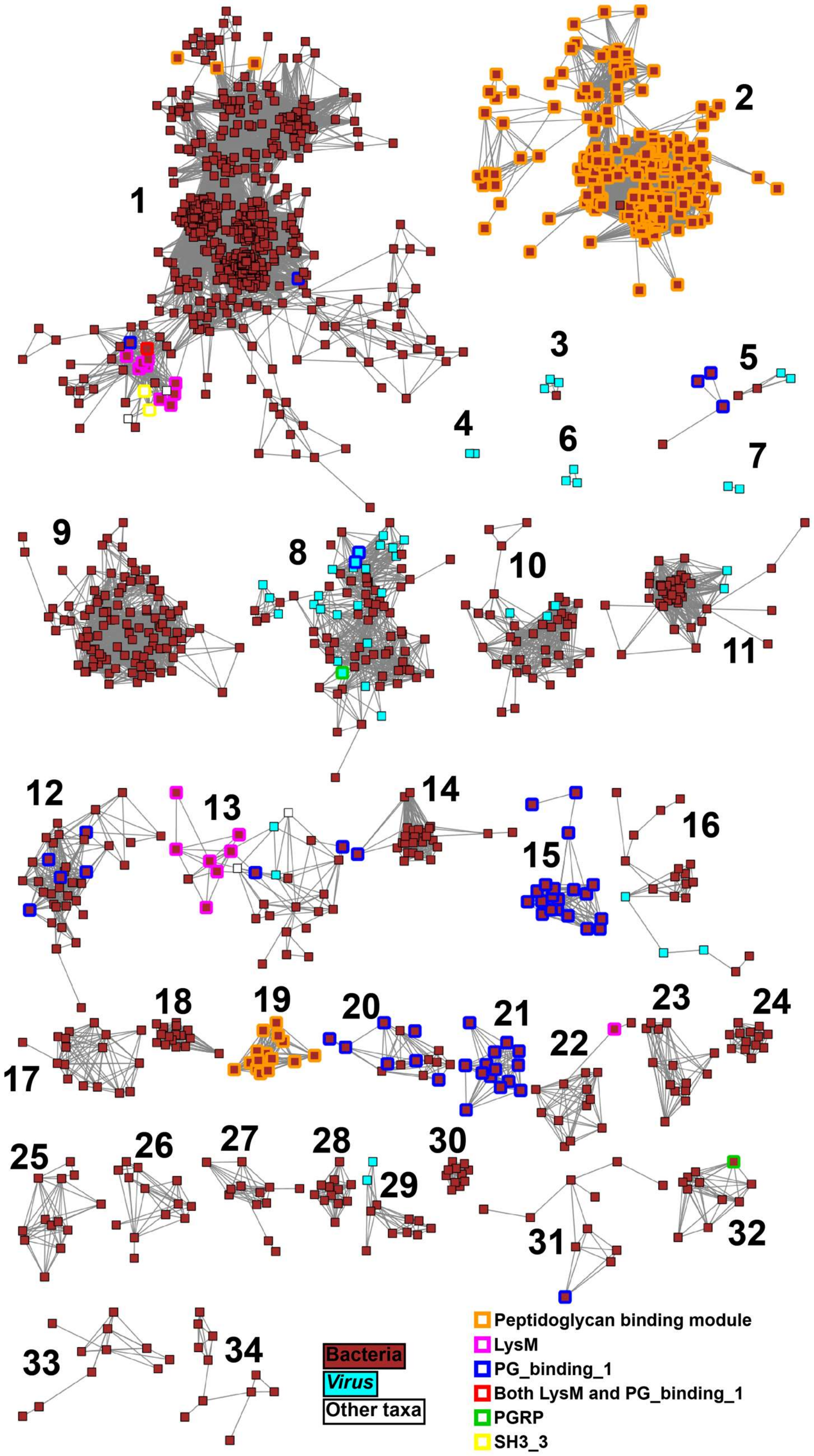

Figure S7. Accessory binding modules plotted with different colors onto ELYS groups sequence networks. The group identifiers are the same as in **Fig. 3B**.
