# Supplementary material for: The GH19 Engineering Database: Sequence diversity, substrate scope, and evolution in glycoside hydrolase family 19
Source: PLoS One. 2021 Oct 26;16(10):e0256817. doi: 10.1371/journal.pone.0256817 (PMC8547705; doi:10.1371/journal.pone.0256817)
Supplement: S8 Fig — (PDF) [file pone.0256817.s008.pdf]

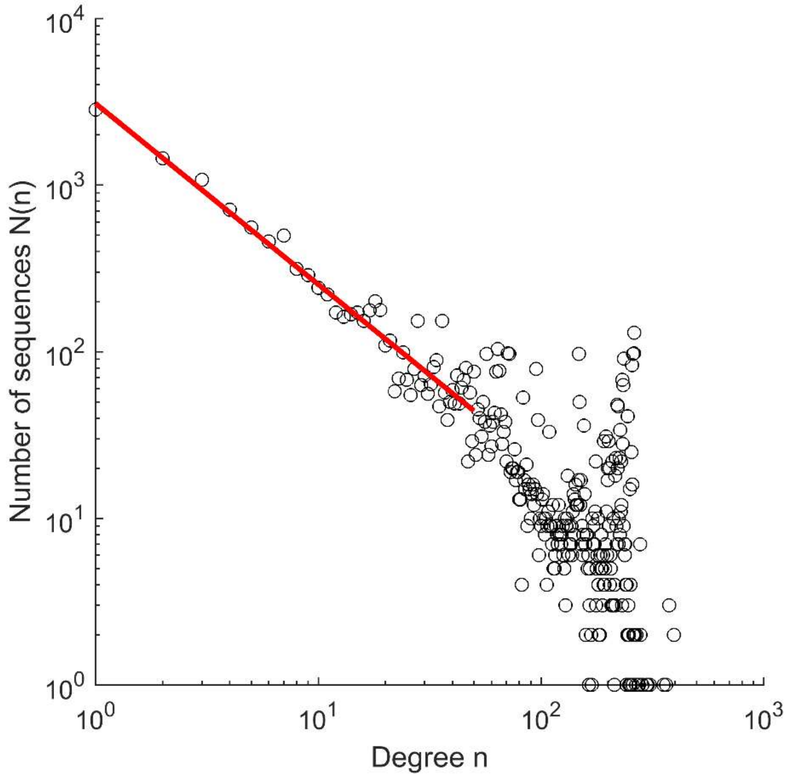

Figure S8. The degree distribution *N(n)* for the catalytic domains from the GH19ED at a threshold of 95% sequence identity was approximated by a power-law for degrees ≤ 50 (red line) yielding a scaling exponent of $\gamma = 1.1$.
