# Supplementary material for: The GH19 Engineering Database: Sequence diversity, substrate scope, and evolution in glycoside hydrolase family 19
Source: PLoS One. 2021 Oct 26;16(10):e0256817. doi: 10.1371/journal.pone.0256817 (PMC8547705; doi:10.1371/journal.pone.0256817)
Supplement: S9 Fig — Histograms of the cluster size distributions N(s) for the catalytic domains from the GH19ED at thresholds of 60% (A), 70% (B), 80% (C), and 90% (D) sequence identity. The distributions for all annotated catalytic domains (depicted in black) were approximated by a power law yielding exponents τh of 0.7, 0.7, 0.8 and 1.1, respectively (compare with S10 Fig). The histogram data for the catalytic domains in the individual CHIT and ELYS subfamilies are depicted as red and blue triangles, respectively. (PDF) [file pone.0256817.s009.pdf]

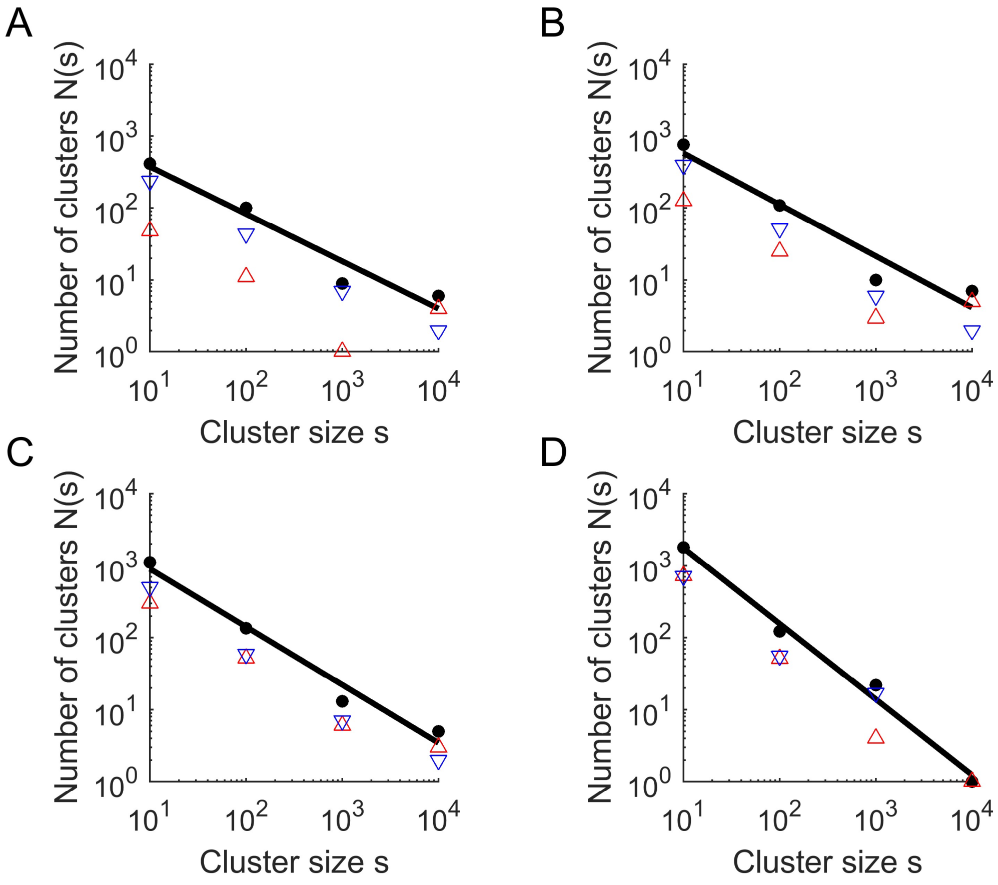

Figure S9. Histograms of the cluster size distributions *N(s)* for the catalytic domains from the GH19ED at thresholds of 60% (**A**), 70% (**B**), 80% (**C**), and 90% (**D**) sequence identity. The distributions for all annotated catalytic domains (depicted in black) were approximated by a power law yielding exponents τ_h of 0.7, 0.7, 0.8 and 1.1, respectively (compare with **Fig. S10**). The histogram data for the catalytic domains in the individual CHIT and ELYS subfamilies are depicted as red and blue triangles, respectively.
