# Supplementary material for: The GH19 Engineering Database: Sequence diversity, substrate scope, and evolution in glycoside hydrolase family 19
Source: PLoS One. 2021 Oct 26;16(10):e0256817. doi: 10.1371/journal.pone.0256817 (PMC8547705; doi:10.1371/journal.pone.0256817)
Supplement: S10 Fig — (PDF) [file pone.0256817.s010.pdf]

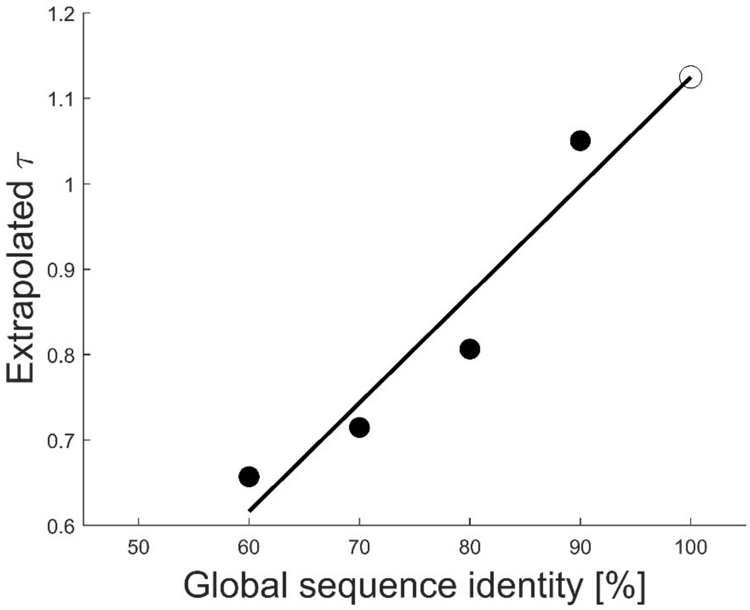

Figure S10. Linear fitting of the slopes of the histograms (**Fig. S9**), used to linearly extrapolate the theoretical exponent τ for individual amino acid exchanges at 100% sequence identity.
