# Supplementary material for: The GH19 Engineering Database: Sequence diversity, substrate scope, and evolution in glycoside hydrolase family 19
Source: PLoS One. 2021 Oct 26;16(10):e0256817. doi: 10.1371/journal.pone.0256817 (PMC8547705; doi:10.1371/journal.pone.0256817)
Supplement: S11 Fig — Rate4Site conservation scores (see Methods section of the main text) are visualized onto models of CHIT reference (A-C, PDB accession 4j0l) and ELYS reference structure (B-D, PDB accession 4ok7). (A) and (B) models are visualized as cartoon with α-helices shown as cylinders, substrate binding residues as sticks (except glycine), and catalytic residues as balls and sticks. (C) and (D) are the same models shown in A and B, represented as solvent accessible surface areas. (PDF) [file pone.0256817.s011.pdf]

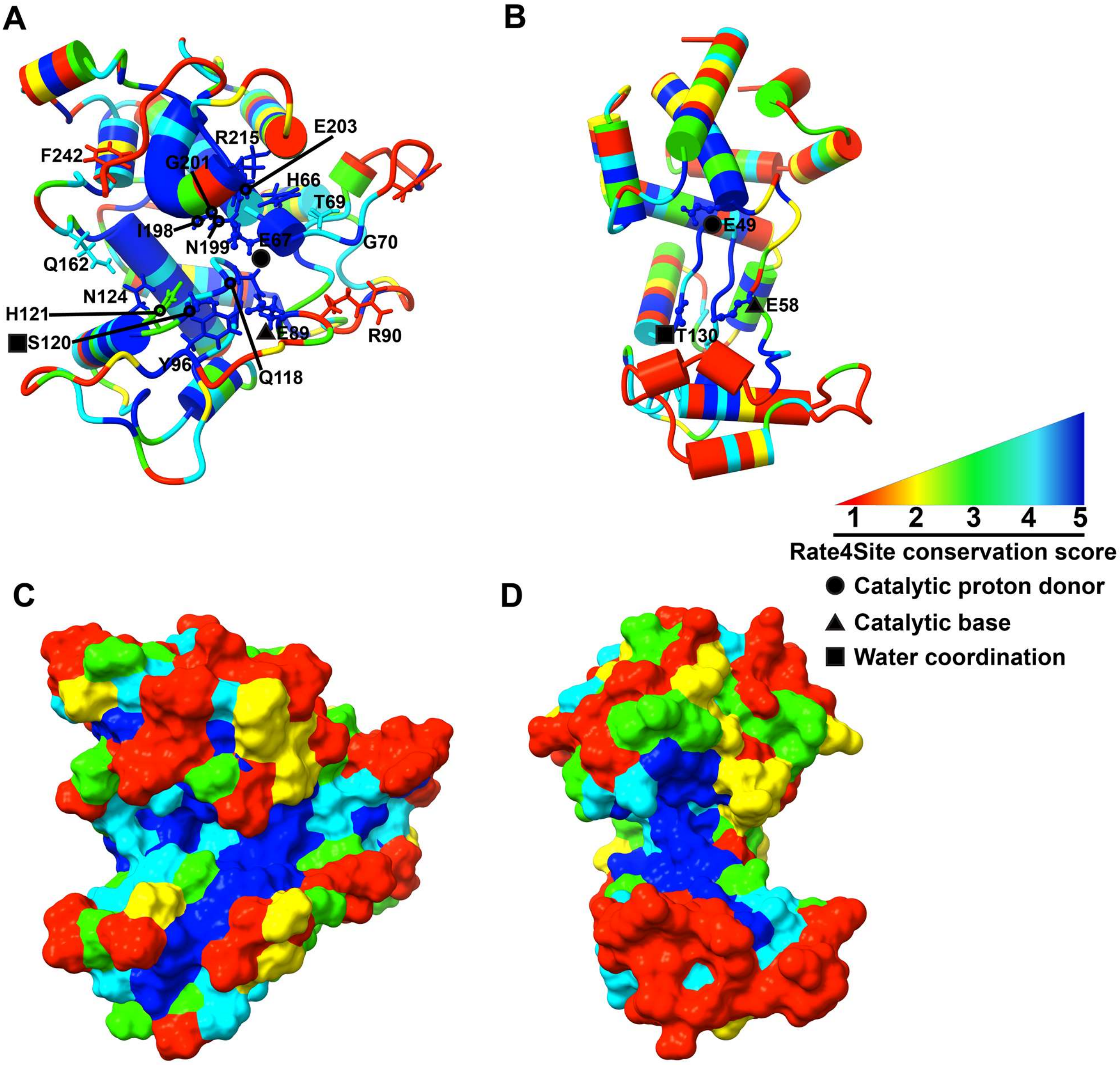

Figure S11. Rate4Site conservation scores (see *Methods* section of the main text) are visualized onto models of CHIT reference (**A**-**C**, PDB accession 4j0l) and ELYS reference structure (**B**-**D**, PDB accession 4ok7). (**A**) and (**B**) models are visualized as cartoon with α-helices shown as cylinders, substrate binding residues as sticks (except glycine), and catalytic residues as balls and sticks. (**C**) and (**D**) are the same models shown in **A** and **B**, represented as solvent accessible surface areas.
