# Supplementary material for: The GH19 Engineering Database: Sequence diversity, substrate scope, and evolution in glycoside hydrolase family 19
Source: PLoS One. 2021 Oct 26;16(10):e0256817. doi: 10.1371/journal.pone.0256817 (PMC8547705; doi:10.1371/journal.pone.0256817)
Supplement: S12 Fig — Rate4Site conservation score 1 (least conserved) and 5 (most conserved), as declared in the Methods section of the main text, are visualized with two different colors (red for score 1 and blue for score 5) plotted onto 3D models of rye seed CHIT reference (A-C for score 5 and B-D for score 1, PDB accession 4jol) and ELYS reference from bacteriophage SPN1S (E-G for score 5 and F-H for score 1, PDB accession 4ok7). (A-B) The CHIT reference model is visualized as cartoon, with substrate binding residues labelled in S11A Fig as sticks (except for glycine). (C-D) The ELYS reference model is visualized in cartoon with residues as sticks if corresponding to CHIT substrate binding residues reported in Table 1. (E-F-G-H) The same models presented above, shown as solvent accessible surface areas. (PDF) [file pone.0256817.s012.pdf]

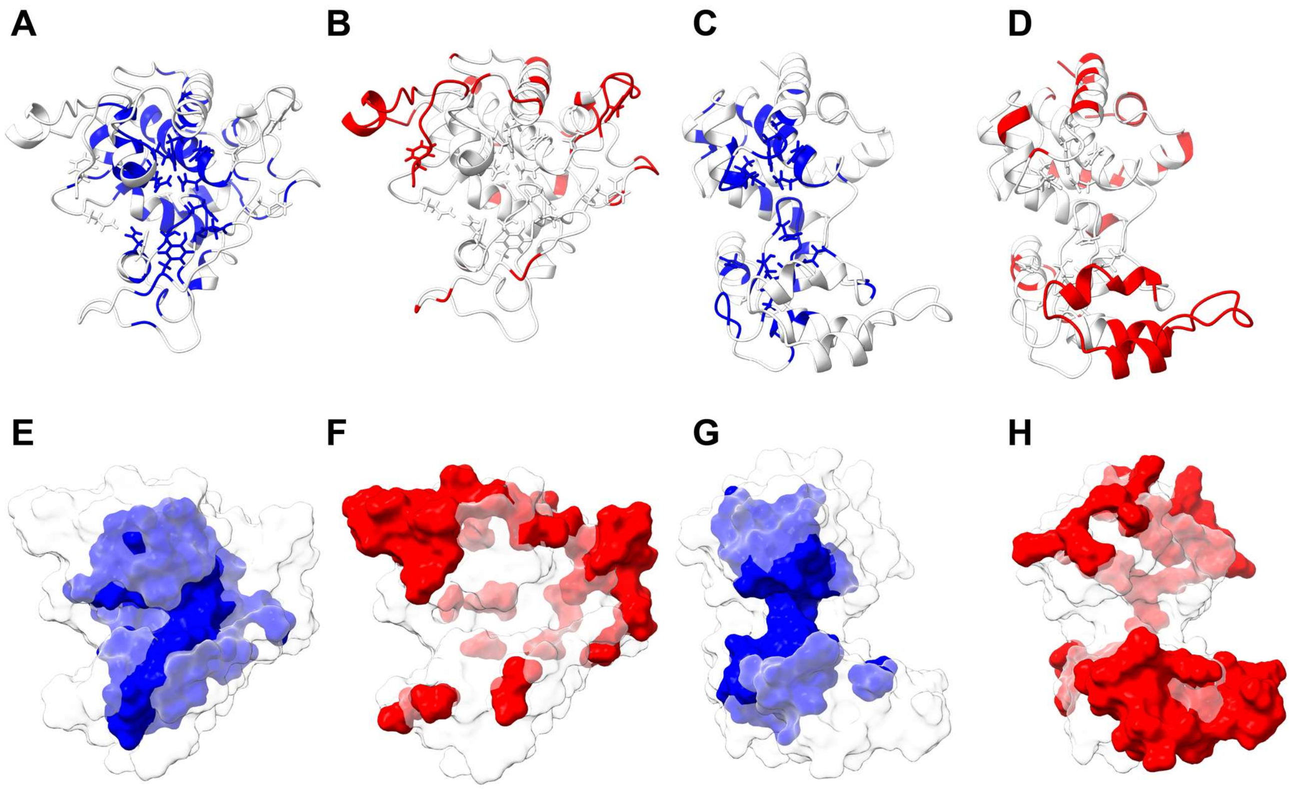

Figure S12. Rate4Site conservation score 1 (least conserved) and 5 (most conserved), as declared in the *Methods* section of the main text, are visualized with two different colors (red for score 1 and blue for score 5) plotted onto 3D models of rye seed CHIT reference (**A-C** for score 5 and **B-D** for score 1, PDB accession 4jol) and ELYS reference from bacteriophage SPN1S (**E-G** for score 5 and **F-H** for score 1, PDB accession 4ok7). (**A-B**) The CHIT reference model is visualized as cartoon, with substrate binding residues labelled in **Fig. S11A** as sticks (except for glycine). (**C-D**) The ELYS reference model is visualized in cartoon with residues as sticks if corresponding to CHIT substrate binding residues reported in **Tab. 1**. (**E-F-G-H**) The same models presented above, shown as solvent accessible surface areas.
