# Supplementary material for: The GH19 Engineering Database: Sequence diversity, substrate scope, and evolution in glycoside hydrolase family 19
Source: PLoS One. 2021 Oct 26;16(10):e0256817. doi: 10.1371/journal.pone.0256817 (PMC8547705; doi:10.1371/journal.pone.0256817)
Supplement: S13 Fig — (A) The structures of GH19 “loopful” chitinase from rye seed Secale cereale (orange, PDB accession 4jol) and “loopless” chitinase from moss Gemmabryum coronatum (cyan, PDB accession 3wh1) superposed with the mmaker command implemented in ChimeraX 0.9, showing in red the five additional loops of “loopful” plant chitinases and the shared loop 3. The two tetra-chitooligosaccharides spanning the catalytic cleft in complex with the crystal structure of rye seed are shown; numbers under sugar moieties are in accordance with the standard nomenclature for GH. Cleavage occurs between units bound in subsites -1 and +1 [140]. (B) The structure of GH19 endolysin from bacteriophage SPN1S (PDB code 4ok7) of Salmonella typhimurium is shown for comparison. (PDF) [file pone.0256817.s013.pdf]

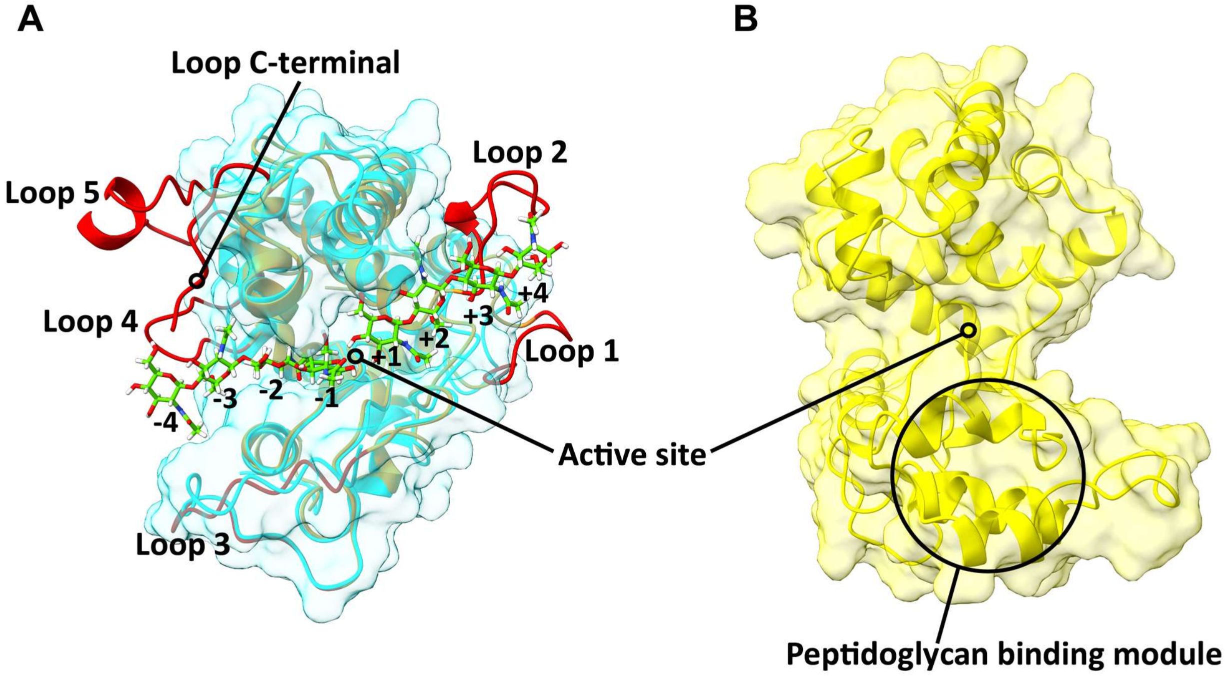

Figure S13. (**A**) The structures of GH19 "loopful" chitinase from rye seed *Secale cereale* (orange, PDB accession 4jol) and "loopless" chitinase from moss *Gemmabryum coronatum* (cyan, PDB accession 3wh1) superposed with the *mmaker* command implemented in ChimeraX 0.9, showing in red the five additional loops of "loopful" plant chitinases and the shared loop 3. The two tetra-chitooligosaccharides spanning the catalytic cleft in complex with the crystal structure of rye seed are shown; numbers under sugar moieties are in accordance with the standard nomenclature for GH. Cleavage occurs between units bound in subsites -1 and +1 [140]. (**B**) The structure of GH19 endolysin from bacteriophage SPN1S (PDB code 4ok7) of *Salmonella typhimurium* is shown for comparison.
