# Supplementary material for: The GH19 Engineering Database: Sequence diversity, substrate scope, and evolution in glycoside hydrolase family 19
Source: PLoS One. 2021 Oct 26;16(10):e0256817. doi: 10.1371/journal.pone.0256817 (PMC8547705; doi:10.1371/journal.pone.0256817)
Supplement: S14 Fig — The black arrow indicates the minimum length threshold used to define the presence of a loop, as specified in the Methods section. The red arrow indicates the threshold used to separate the two modes of length observed for loops 2 and 3. (PDF) [file pone.0256817.s014.pdf]

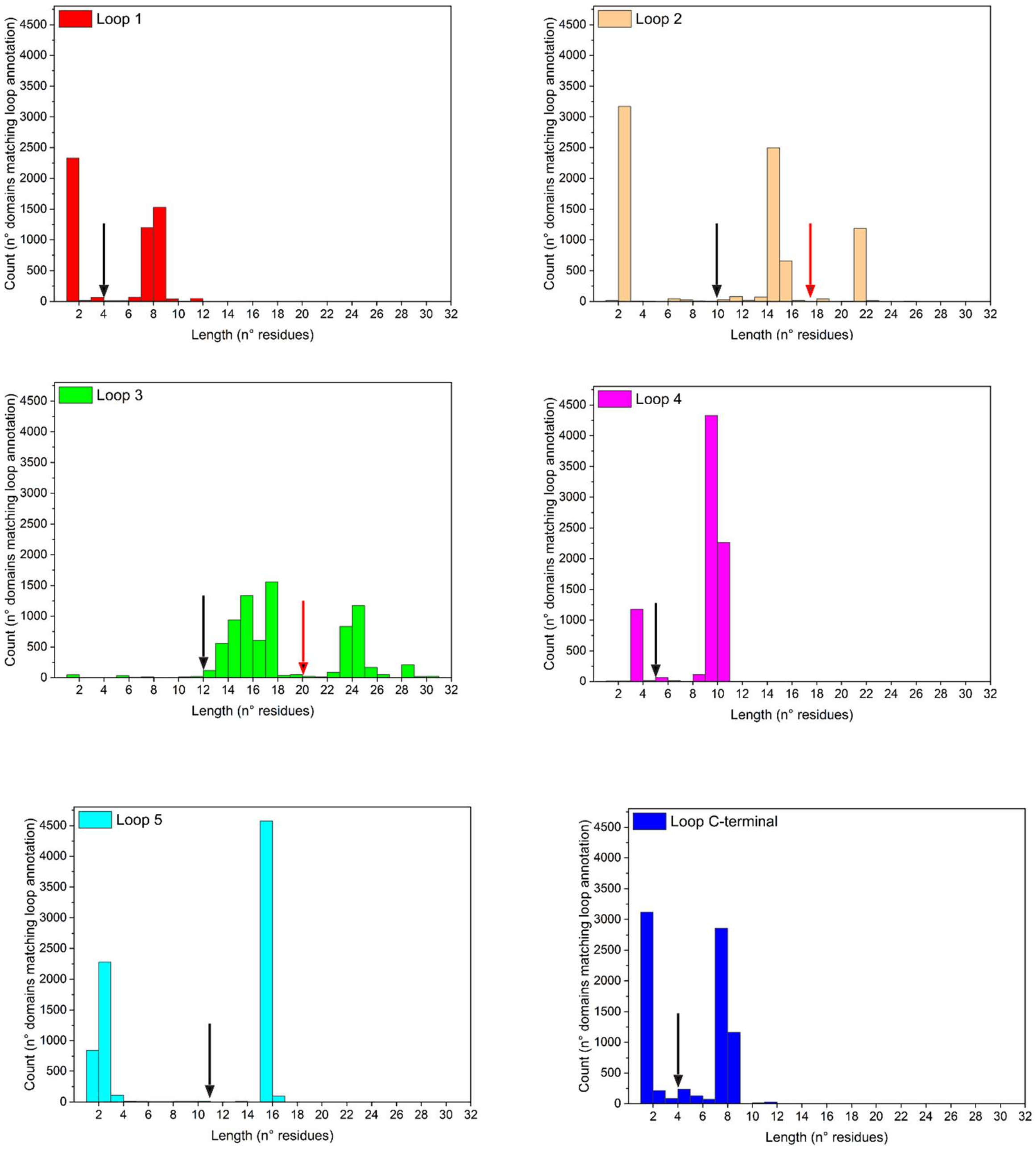

Figure S14. Length distribution of CHIT loop motifs. The black arrow indicates the minimum length threshold used to define the presence of a loop, as specified in the *Methods* section. The red arrow indicates the threshold used to separate the two modes of length observed for loops 2 and 3.
