# Supplementary material for: The GH19 Engineering Database: Sequence diversity, substrate scope, and evolution in glycoside hydrolase family 19
Source: PLoS One. 2021 Oct 26;16(10):e0256817. doi: 10.1371/journal.pone.0256817 (PMC8547705; doi:10.1371/journal.pone.0256817)
Supplement: S15 Fig — Different colors are used to indicate different clusters obtained by the CD-HIT clustering analysis, if more than one cluster is present in each group. A legend is provided with a Roman numeral code corresponding to the group sub-cluster reported on sequence headers in Fig 5. (PDF) [file pone.0256817.s015.pdf]

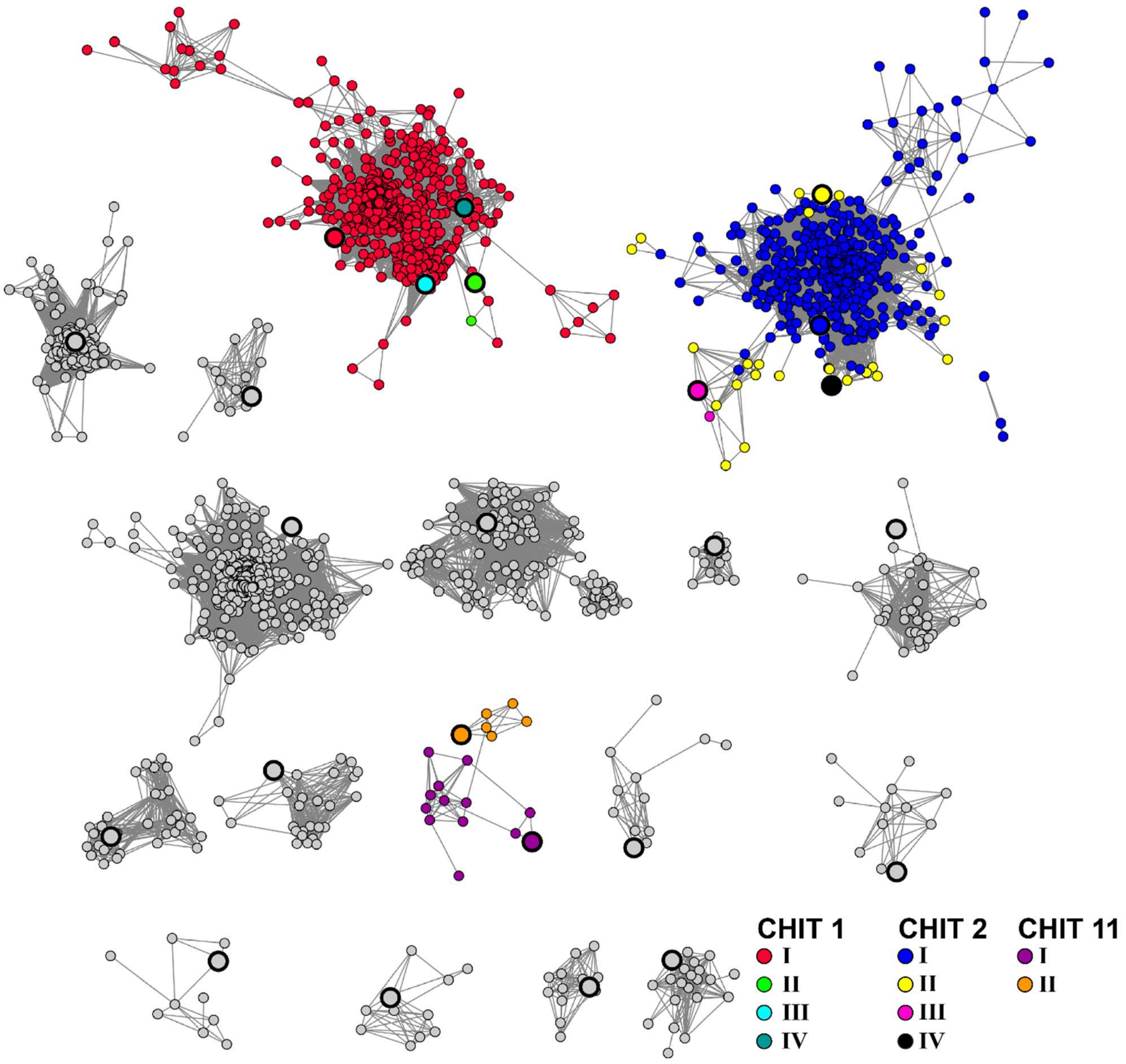

Figure S15 Protein sequence networks of representative domains of CHITs in **Fig. 3A** in which the centroids used for the phylogenetic analysis reported in **Fig. 5** are marked. Different colors are used to indicate different clusters obtained by the CD-HIT clustering analysis, if more than one cluster is present in each group. A legend is provided with a Roman numeral code corresponding to the group sub-cluster reported on sequence headers in **Fig. 5**.
