# Supplementary material for: The GH19 Engineering Database: Sequence diversity, substrate scope, and evolution in glycoside hydrolase family 19
Source: PLoS One. 2021 Oct 26;16(10):e0256817. doi: 10.1371/journal.pone.0256817 (PMC8547705; doi:10.1371/journal.pone.0256817)
Supplement: S17 Fig — (A) The rye seed chitinase model (PDB accession 4j0l) is visualized in blue transparent solvent accessible surface area (loops 1, 2, 5 and C-terminal are colored in red), superposed to the endolysin from bacteriophage SPN1S model (PDB accession 4ok7), visualized in yellow solvent accessible surface area; two co-crystallized (GlcNAc)4-6 are in the catalytic cleft [25]. (B) The same object is rotated by 90° around the vertical axis. Black arrows highlight the regions in which the cleft of the chitinase model is tighter than the one of the endolysin. (PDF) [file pone.0256817.s017.pdf]

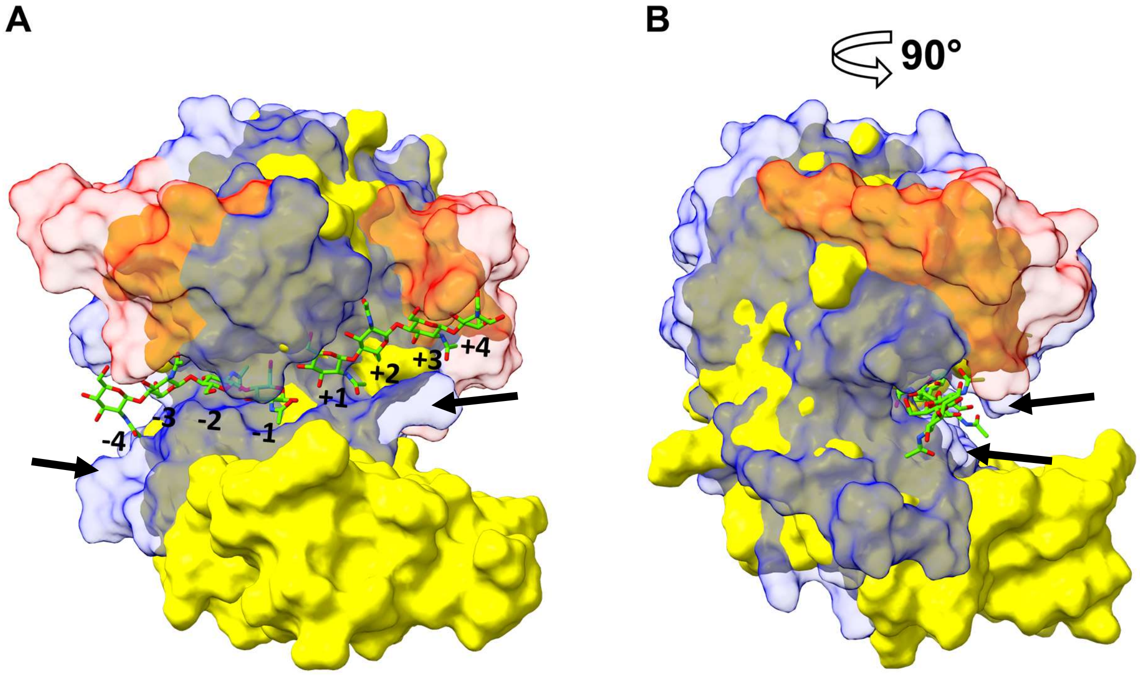

Figure S17. (**A**) The rye seed chitinase model (PDB accession 4j0l) is visualized in blue transparent solvent accessible surface area (loops 1, 2, 5 and C-terminal are colored in red), superposed to the endolysin from bacteriophage SPN1S model (PDB accession 4ok7), visualized in yellow solvent accessible surface area; two co-crystallized $(GlcNAc)_{4-6}$ are in the catalytic cleft [25]. (**B**) The same object is rotated by 90° around the vertical axis. Black arrows highlight the regions in which the cleft of the chitinase model is tighter than the one of the endolysin.

Supplementary references

1. Verburg JG, Smith C, Lisek C, Huynh QK. Identification of an essential tyrosine residue in the catalytic site of a chitinase isolated from Zea mays that is selectively modified during inactivation with 1-ethyl-3-(3-dimethylaminopropyl)-carbodiimide. Journal of Biological Chemistry. 1992;267(6):3886-93.

2. Chaudet MM, Naumann TA, Price NP, Rose DR. Crystallographic structure of ChitA, a glycoside hydrolase family 19, plant class IV chitinase from Zea mays. Protein Science. 2014;23(5):586-93.

3. Volpicella M, Leoni C, Fanizza I, Distaso M, Leoni G, Farioli L, et al. Characterization of maize chitinase-A, a tough allergenic molecule. Allergy. 2017;72(9):1423-9.

4. Pohane AA, Joshi H, Jain V. Molecular Dissection of Phage Endolysin an interdomain interaction confers host specificity in lysin a of mycobacterium phage D29. Journal of Biological Chemistry. 2014;289(17):12085-95.

5. Taira T, Mahoe Y, Kawamoto N, Onaga S, Iwasaki H, Ohnuma T, et al. Cloning and characterization of a small family 19 chitinase from moss (Bryum coronatum). Glycobiology. 2011;21(5):644-54.

6. Ohnuma T, Sørlie M, Fukuda T, Kawamoto N, Taira T, Fukamizo T. Chitin oligosaccharide binding to a family GH19 chitinase from the moss Bryum coronatum. The FEBS journal. 2011;278(21):3991-4001.

7. Ohnuma T, Umemoto N, Nagata T, Shinya S, Numata T, Taira T, et al. Crystal structure of a “loopless” GH19 chitinase in complex with chitin tetrasaccharide spanning the catalytic center. Biochimica et Biophysica Acta (BBA)-Proteins and Proteomics. 2014;1844(4):793-802.

8. Takenaka S, Ohnuma T, Fukamizo T. Insertion of a Loop Structure into the “Loopless” GH19 Chitinase from Bryum coronatum. Journal of Applied Glycoscience. 2017;64(2):39-42.

9. Ohnuma T, Tanaka T, Urasaki A, Dozen S, Fukamizo T. A novel method for chemo-enzymatic synthesis of chitin oligosaccharide catalyzed by the mutant of inverting family GH19 chitinase using 4, 6-dimethoxy-1, 3, 5-triazin-2-yl α-chitobioside as a glycosyl donor. The Journal of Biochemistry. 2018;165(6):497-503.

10. Ueda M, Kojima M, Yoshikawa T, Mitsuda N, Araki K, Kawaguchi T, et al. A novel type of family 19 chitinase from Aeromonas sp. No. 10S-24: Cloning, sequence, expression, and the enzymatic properties. European journal of biochemistry. 2003;270(11):2513-20.

11. Kojima M, Yoshikawa T, Ueda M, Nonomura T, Matsuda Y, Toyoda H, et al. Family 19 chitinase from Aeromonas sp. No. 10S-24: role of chitin-binding domain in the enzymatic activity. Journal of biochemistry. 2005;137(2):235-42.

12. Han B, Zhou K, Li Z, Sun B, Ni Q, Meng X, et al. Characterization of the first fungal Glycosyl Hydrolase family 19 chitinase (NbchiA) from Nosema bombycis (Nb). Journal of Eukaryotic Microbiology. 2016;63(1):37-45.

13. Ohno T, Armand S, Hata T, Nikaidou N, Henrissat B, Mitsutomi M, et al. A modular family 19 chitinase found in the prokaryotic organism Streptomyces griseus HUT 6037. Journal of bacteriology. 1996;178(17):5065-70.

14. Watanabe T, Kanai R, Kawase T, Tanabe T, Mitsutomi M, Sakuda S, et al. Family 19 chitinases of Streptomyces species: characterization and distribution. Microbiology. 1999;145(12):3353-63.

15. Itoh Y, Takahashi K, Takizawa H, Nikaidou N, Tanaka H, Nishihashi H, et al. Family 19 chitinase of Streptomyces griseus HUT6037 increases plant resistance to the fungal disease. Bioscience, biotechnology, and biochemistry. 2003;67(4):847-55.

16. Tang CM, Chye M-L, Ramalingam S, Ouyang S-W, Zhao K-J, Ubhayasekera W, et al. Functional analyses of the chitin-binding domains and the catalytic domain of Brassica juncea chitinase BjCHI1. Plant molecular biology. 2004;56(2):285-98.

17. Kezuka Y, Ohishi M, Itoh Y, Watanabe J, Mitsutomi M, Watanabe T, et al. Structural studies of a two-domain chitinase from Streptomyces griseus HUT6037. Journal of molecular biology. 2006;358(2):472-84.

18. Akagi K-i, Watanabe J, Hara M, Kezuka Y, Chikaishi E, Yamaguchi T, et al. Identification of the substrate interaction region of the chitin-binding domain of Streptomyces griseus chitinase C. Journal of biochemistry. 2006;139(3):483-93.

19. Ubhayasekera W, Tang CM, Ho SW, Berglund G, Bergfors T, Chye ML, et al. Crystal structures of a family 19 chitinase from Brassica juncea show flexibility of binding cleft loops. The FEBS journal. 2007;274(14):3695-703.

20. Yamagami T, Funatsu G. Purification and some properties of three chitinases from the seeds of rye (Secale cereale). Bioscience, biotechnology, and biochemistry. 1993;57(4):643-7.

21. Yamagami T, Funatsu G. Identification of the tryptophan residue located at the substrate-binding site of rye seed chitinase-c. Bioscience, biotechnology, and biochemistry. 1995;59(6):1076-81.

22. Ohnuma T, YAGi M, Yamagami T, Taira T, Aso Y, Ishiguro M. Molecular cloning, functional expression, and mutagenesis of cDNA encoding rye (Secale cereale) seed chitinase-c. Bioscience, biotechnology, and biochemistry. 2002;66(2):277-84.

23. Taira T, Ohnuma T, Yamagami T, Aso Y, Ishiguro M, Ishihara M. Antifungal activity of rye (Secale cereale) seed chitinases: the different binding manner of class I and class II chitinases to the fungal cell walls. Bioscience, biotechnology, and biochemistry. 2002;66(5):970-7.

24. Ohnuma T, Numata T, Osawa T, Inanaga H, Okazaki Y, Shinya S, et al. Crystal structure and chitin oligosaccharide-binding mode of a 'loopful'family GH19 chitinase from rye, Secale cereale, seeds. The FEBS journal. 2012;279(19):3639-51.

25. Ohnuma T, Umemoto N, Kondo K, Numata T, Fukamizo T. Complete subsite mapping of a "loopful" GH19 chitinase from rye seeds based on its crystal structure. FEBS letters. 2013;587(16):2691-7.

26. Hoster F, Schmitz JE, Daniel R. Enrichment of chitinolytic microorganisms: isolation and characterization of a chitinase exhibiting antifungal activity against phytopathogenic fungi from a novel Streptomyces strain. Applied Microbiology and Biotechnology. 2005;66(4):434-42.

27. Honda Y, Taniguchi H, Kitaoka M. A reducing-end-acting chitinase from Vibrio proteolyticus belonging to glycoside hydrolase family 19. Applied microbiology and biotechnology. 2008;78(4):627-34.

28. García-Fraga B, da Silva AF, López-Seijas J, Sieiro C. A novel family 19 chitinase from the marine-derived Pseudoalteromonas tunicata CCUG 44952T: Heterologous expression, characterization and antifungal activity. Biochemical engineering journal. 2015;93:84-93.

29. Osswald WF, Shapiro JP, Doostdar H, McDonald RE, Niedz RP, Nairn CJ, et al. Identification and characterization of acidic hydrolases with chitinase and chitosanase activities from sweet orange callus tissue. Plant and cell physiology. 1994;35(5):811-20.

30. Nielsen KK, Bojsen K, Roepstorff P, Mikkelsen JD. A hydroxyproline-containing class IV chitinase of sugar beet is glycosylated with xylose. Plant Molecular Biology. 1994;25(2):241-57.

31. Schultze M, Staehelin C, Brunner F, Genetet I, Legrand M, Fritig B, et al. Plant chitinase/lysozyme isoforms show distinct substrate specificity and cleavage site preference towards lipochitooligosaccharide Nod signals. The Plant Journal. 1998;16(5):571-80.

32. Yerzhebayeva R, Abekova A, Konysbekov K, Bastaubayeva S, Kabdrakhmanova A, Absattarova A, et al. Two sugar beet chitinase genes, BvSP2 and BvSE2, analysed with SNP Amplifluor-like markers, are highly expressed after Fusarium root rot inoculations and field susceptibility trial. PeerJ. 2018;6:e5127.

33. Kim J-K, Jang I-C, Wu R, Zuo W-N, Boston RS, Lee Y-H, et al. Co-expression of a modified maize ribosome-inactivating protein and a rice basic chitinase gene in transgenic rice plants confers enhanced resistance to sheath blight. Transgenic Research. 2003;12(4):475-84.

34. Takakura Y, Ito T, Saito H, Inoue T, Komari T, Kuwata S. Flower-predominant expression of a gene encoding a novel class I chitinase in rice (Oryza sativa L.). Plant molecular biology. 2000;42(6):883-97.

35. Legrand M, Kauffmann S, Geoffroy P, Fritig B. Biological function of pathogenesis-related proteins: four tobacco pathogenesis-related proteins are chitinases. Proceedings of the National Academy of Sciences. 1987;84(19):6750-4.

36. Shinshi H, Mohnen D, Meins F. Regulation of a plant pathogenesis-related enzyme: inhibition of chitinase and chitinase mRNA accumulation in cultured tobacco tissues by auxin and cytokinin. Proceedings of the National Academy of Sciences. 1987;84(1):89-93.

37. Brunner F, Stintzi A, Fritig B, Legrand M. Substrate specificities of tobacco chitinases. The Plant Journal. 1998;14(2):225-34.

38. Yeh S, Moffatt BA, Griffith M, Xiong F, Yang DS, Wiseman SB, et al. Chitinase genes responsive to cold encode antifreeze proteins in winter cereals. Plant Physiology. 2000;124(3):1251-64.

39. Huet J, Wyckmans J, Wintjens R, Boussard P, Raussens V, Vandenbussche G, et al. Structural characterization of two papaya chitinases, a family GH19 of glycosyl hydrolases. Cellular and Molecular Life Sciences CMLS. 2006;63(24):3042-54.

40. Huet J, Rucktooa P, Clantin B, Azarkan M, Looze Y, Villeret V, et al. X-ray structure of papaya chitinase reveals the substrate binding mode of glycosyl hydrolase family 19 chitinases. Biochemistry. 2008;47(32):8283-91.

41. Leah R, Tommerup H, Svendsen I, Mundy J. Biochemical and molecular characterization of three barley seed proteins with antifungal properties. Journal of Biological Chemistry. 1991;266(3):1564-73.

42. Song HK, Suh SW. Refined structure of the chitinase from barley seeds at 2.0 Å resolution. Acta Crystallographica Section D: Biological Crystallography. 1996;52(2):289-98.

43. ANDERSEN MD, JENSEN A, ROBERTUS JD, Robert L, SKRIVER K. Heterologous expression and characterization of wild-type and mutant forms of a 26 kDa endochitinase from barley (Hordeum vulgare L.). Biochemical journal. 1997;322(3):815-22.

44. Hollis T, Honda Y, Fukamizo T, Marcotte E, Day PJ, Robertus JD. Kinetic analysis of barley chitinase. Archives of biochemistry and biophysics. 1997;344(2):335-42.

45. Honda Y, Fukamizo T. Substrate binding subsites of chitinase from barley seeds and lysozyme from goose egg white. Biochimica et Biophysica Acta (BBA)-Protein Structure and Molecular Enzymology. 1998;1388(1):53-65.

46. Brameld KA, Goddard WA. The role of enzyme distortion in the single displacement mechanism of family 19 chitinases. Proceedings of the National Academy of Sciences. 1998;95(8):4276-81.

47. Ohnishi T, Juffer AH, Tamoi M, Skriver K, Fukamizo T. 26 kDa endochitinase from barley seeds: an interaction of the ionizable side chains essential for catalysis. Journal of biochemistry. 2005;138(5):553-62.

48. Fukamizo T, Miyake R, Tamura A, Ohnuma T, Skriver K, Pursiainen NV, et al. A flexible loop controlling the enzymatic activity and specificity in a glycosyl hydrolase family 19 endochitinase from barley seeds (Hordeum vulgare L.). Biochimica et Biophysica Acta (BBA)-Proteins and Proteomics. 2009;1794(8):1159-67.

49. Letzel T, Sahmel-Schneider E, Skriver K, Ohnuma T, Fukamizo T. Chitinase-catalyzed hydrolysis of 4-nitrophenyl penta-N-acetyl-β-chitopentaoside as determined by real-time ESIMS: The 4-nitrophenyl moiety of the substrate interacts with the enzyme binding site. Carbohydrate research. 2011;346(6):863-6.

50. Kirubakaran SI, Sakthivel N. Cloning and overexpression of antifungal barley chitinase gene in Escherichia coli. Protein expression and purification. 2007;52(1):159-66.

51. Datta K, Tu J, Oliva N, Ona I, Velazhahan R, Mew TW, et al. Enhanced resistance to sheath blight by constitutive expression of infection-related rice chitinase in transgenic elite indica rice cultivars. Plant Science. 2001;160(3):405-14.

52. Mizuno R, Fukamizo T, Sugiyama S, Nishizawa Y, Kezuka Y, Nonaka T, et al. Role of the loop structure of the catalytic domain in rice class I chitinase. Journal of biochemistry. 2008;143(4):487-95.

53. Mizuno R, Itoh Y, Nishizawa Y, Kezuka Y, Suzuki K, Nonaka T, et al. Purification and characterization of a rice class I chitinase, OsChia1b, produced in Esherichia coli. Bioscience, biotechnology, and biochemistry. 2008;72(3):893-5.

54. Kezuka Y, Kojima M, Mizuno R, Suzuki K, Watanabe T, Nonaka T. Structure of full-length class I chitinase from rice revealed by X-ray crystallography and small-angle X-ray scattering. Proteins: Structure, Function, and Bioinformatics. 2010;78(10):2295-305.

55. Kaomek M, Mizuno K, Fujimura T, Sriyotha P, Cairns JRK. Cloning, expression, and characterization of an antifungal chitinase from Leucaena leucocephala de Wit. Bioscience, biotechnology, and biochemistry. 2003;67(4):667-76.

56. Xu Y, Zhu Q, Panbangred W, Shirasu K, Lamb C. Regulation, expression and function of a new basic chitinase gene in rice (Oryza sativa L.). Plant molecular biology. 1996;30(3):387-401.

57. Nielsen K, Jørgensen P, Mikkelsen J. Antifungal activity of sugar beet chitinase against Cercospora beticola: an autoradiographic study on cell wall degradation. Plant Pathology. 1994;43(6):979-86.

58. Mavrodi DV, Loper JE, Paulsen IT, Thomashow LS. Mobile genetic elements in the genome of the beneficial rhizobacterium Pseudomonas fluorescens Pf-5. BMC microbiology. 2009;9(1):8.

59. Chen A, Yu L, Fan J, Feng D, Wang J. The expression, purification and activity analysis of the rice chitinase gene in Escherichia coli. Sheng wu gong cheng xue bao= Chinese journal of biotechnology. 2008;24(2):188-92.

60. Lerner DR, Raikhel NV. The gene for stinging nettle lectin (Urtica dioica agglutinin) encodes both a lectin and a chitinase. Journal of Biological Chemistry. 1992;267(16):11085-91.

61. Does MP, Houterman PM, Dekker HL, Cornelissen BJ. Processing, targeting, and antifungal activity of stinging nettle agglutinin in transgenic tobacco. Plant Physiology. 1999;120(2):421-32.

62. Saul FA, Rovira P, Boulot G, Van Damme EJ, Peumans WJ, Truffa-Bachi P, et al. Crystal structure of Urtica dioica agglutinin, a superantigen presented by MHC molecules of class I and class II. Structure. 2000;8(6):593-603.

63. Harata K, Schubert W-D, Muraki M. Structure of Urtica dioica agglutinin isolectin I: dimer formation mediated by two zinc ions bound at the sugar-binding site. Acta Crystallographica Section D: Biological Crystallography. 2001;57(11):1513-7.

64. Paszota P, Escalante-Perez M, Thomsen LR, Risør MW, Dembski A, Sanglas L, et al. Secreted major Venus flytrap chitinase enables digestion of arthropod prey. Biochimica et Biophysica Acta (BBA)-Proteins and Proteomics. 2014;1844(2):374-83.

65. Kragh KM, Hendriks T, de Jong AJ, Schiavo FL, Bucherna N, Højrup P, et al. Characterzation of chitinases able to rescue somatic embryos of the temperature-sensitive carrot variantts11. Plant molecular biology. 1996;31(3):631-45.

66. Kolosova N, Breuil C, Bohlmann J. Cloning and characterization of chitinases from interior spruce and lodgepole pine. Phytochemistry. 2014;101:32-9.

67. Tsujibo H, Okamoto T, Hatano N, Miyamoto K, Watanabe T, Mitsutomi M, et al. Family 19 chitinases from Streptomyces thermoviolaceus OPC-520: molecular cloning and characterization. Bioscience, biotechnology, and biochemistry. 2000;64(11):2445-53.

68. Walmagh M, Briers Y, Dos Santos SB, Azeredo J, Lavigne R. Characterization of modular bacteriophage endolysins from Myoviridae phages OBP, 201φ2-1 and PVP-SE1. PLoS One. 2012;7(5):e36991.

69. Yano S, Rattanakit N, Wakayama M, Tachiki T. A chitinase indispensable for formation of protoplast of Schizophyllum commune in basidiomycete-lytic enzyme preparation produced by Bacillus circulans KA-304. Bioscience, biotechnology, and biochemistry. 2004;68(6):1299-305.

70. Yano S, Rattanakit N, Wakayama M, TACHIKI T. Cloning and expression of a Bacillus circulans KA-304 gene encoding chitinase I, which participates in protoplast formation of Schizophyllum commune. Bioscience, biotechnology, and biochemistry. 2005;69(3):602-9.

71. Yano S, Suyotha W, Honda A, Takagi K, Rattanakit-Chandet N, Wakayama M, et al. N-terminal region of chitinase I of Bacillus circulans KA-304 contained new chitin-biding domain. Bioscience, biotechnology, and biochemistry. 2011;75(2):299-304.

72. Saito A, Miyashita K, Biuković G, Schrempf H. Characteristics of a Streptomyces coelicolor A3 (2) extracellular protein targeting chitin and chitosan. Appl Environ Microbiol. 2001;67(3):1268-73.

73. Hoell IA, Dalhus B, Heggset EB, Aspmo SI, Eijsink VG. Crystal structure and enzymatic properties of a bacterial family 19 chitinase reveal differences from plant enzymes. The FEBS journal. 2006;273(21):4889-900.

74. Heggset EB, Hoell IA, Kristoffersen M, Eijsink VG, Vårum KM. Degradation of chitosans with chitinase G from Streptomyces coelicolor A3 (2): production of chito-oligosaccharides and insight into subsite specificities. Biomacromolecules. 2009;10(4):892-9.

75. Nakamura T, Ishikawa M, Nakatani H, Oda A. Characterization of cold-responsive extracellular chitinase in bromegrass cell cultures and its relationship to antifreeze activity. Plant Physiology. 2008;147(1):391-401.

76. Landim PGC, Correia TO, Silva FD, Nepomuceno DR, Costa HP, Pereira HM, et al. Production in Pichia pastoris, antifungal activity and crystal structure of a class I chitinase from cowpea (Vigna unguiculata): Insights into sugar binding mode and hydrolytic action. Biochimie. 2017;135:89-103.

77. Truong N-H, Park S-M, Nishizawa Y, Watanabe T, Sasaki T, Itoh Y. Structure, heterologous expression, and properties of rice (Oryza sativa L.) family 19 chitinases. Bioscience, biotechnology, and biochemistry. 2003;67(5):1063-70.

78. Allona I, Collada C, Casado R, Paz-Ares J, Aragoncillo C. Bacterial expression of an active class Ib chitinase from Castanea sativa cotyledons. Plant molecular biology. 1996;32(6):1171-6.

79. Garcia-Casado G, Collada C, Allona I, Casado R, Pacios LF, Aragoncillo C, et al. Site-directed mutagenesis of active site residues in a class I endochitinase from chestnut seeds. Glycobiology. 1998;8(10):1021-8.

80. Schlesier B, Koch G, Horstmann C. Characterization of a class II chitinase from jack bean (Canavalia ensiformis) seeds. Food/Nahrung. 1998;42(03-04):170-.

81. Hahn M, Hennig M, Schlesier B, Höhne W. Structure of jack bean chitinase. Acta Crystallographica Section D: Biological Crystallography. 2000;56(9):1096-9.

82. Huynh QK, Hironaka CM, Levine EB, Smith C, Borgmeyer J, Shah D. Antifungal proteins from plants. Purification, molecular cloning, and antifungal properties of chitinases from maize seed. Journal of Biological Chemistry. 1992;267(10):6635-40.

83. Liu ZH, Wang YC, Qi XT, Yang CP. Cloning and characterization of a chitinase gene Lbchi31 from Limonium bicolor and identification of its biological activity. Molecular biology reports. 2010;37(5):2447-53.

84. Huang L, Garbulewska E, Sato K, Kato Y, Nogawa M, Taguchi G, et al. Isolation of genes coding for chitin-degrading enzymes in the novel chitinolytic bacterium, Chitiniphilus shinanonensis, and characterization of a gene coding for a family 19 chitinase. Journal of bioscience and bioengineering. 2012;113(3):293-9.

85. Robinson SP, Jacobs AK, Dry IB. A class IV chitinase is highly expressed in grape berries during ripening. Plant physiology. 1997;114(3):771-8.

86. Taira T, Yamagami T, Aso Y, Ishiguro M, Ishihara M. Localization, accumulation, and antifungal activity of chitinases in rye (Secale cereale) seed. Bioscience, biotechnology, and biochemistry. 2001;65(12):2710-8.

87. Ohnuma T, Taira T, Yamagami T, Aso Y, Ishiguro M. Molecular cloning, functional expression, and mutagenesis of cDNA encoding class I chitinase from rye (Secale cereale) seeds. Bioscience, biotechnology, and biochemistry. 2004;68(2):324-32.

88. Payne G, Ahl P, Moyer M, Harper A, Beck J, Meins F, et al. Isolation of complementary DNA clones encoding pathogenesis-related proteins P and Q, two acidic chitinases from tobacco. Proceedings of the National Academy of Sciences. 1990;87(1):98-102.

89. Chlan CA, Bourgeois RP. Class I chitinases in cotton (Gossypium hirsutum): characterization, expression and purification. Plant Science. 2001;161(1):143-54.

90. Nakamura S, Iwai T, Honkura R, UGAKI M, OHSHIMA M, OHASHI Y. Four chitinase cDNAs from Chenopodium amaranticolor. Plant Biotechnology. 1997;14(1):85-6.

91. Shimosaka M, Fukumori Y, Narita T, Zhang X-Y, Kodaira R, Nogawa M, et al. The bacterium Burkholderia gladioli strain CHB101 produces two different kinds of chitinases belonging to families 18 and 19 of the glycosyl hydrolases. Journal of bioscience and bioengineering. 2001;91(1):103-5.

92. Wiweger M, Farbos I, Ingouff M, Lagercrantz U, Von Arnold S. Expression of Chia4-Pa chitinase genes during somatic and zygotic embryo development in Norway spruce (Picea abies): similarities and differences between gymnosperm and angiosperm class IV chitinases. Journal of experimental botany. 2003;54(393):2691-9.

93. Ubhayasekera W, Rawat R, Ho SWT, Wiweger M, Von Arnold S, Chye M-L, et al. The first crystal structures of a family 19 class IV chitinase: the enzyme from Norway spruce. Plant molecular biology. 2009;71(3):277-89.

94. Verburg JG, Rangwala SH, Samac DA, Luckow VA, Huynh QK. Examination of the role of tyrosine-174 in the catalytic mechanism of the Arabidopsis thaliana-chitinase: comparison of variant chitinases generated by site-directed mutagenesis and expressed in insect cells using baculovirus vectors. Archives of biochemistry and biophysics. 1993;300(1):223-30.

95. Thomma BP, Eggermont K, Penninckx IA, Mauch-Mani B, Vogelsang R, Cammue BP, et al. Separate jasmonate-dependent and salicylate-dependent defense-response pathways in Arabidopsis are essential for resistance to distinct microbial pathogens. Proceedings of the National Academy of Sciences. 1998;95(25):15107-11.

96. Wemmer T, Kaufmann H, Kirch H-H, Schneider K, Lottspeich F, Thompson RD. The most abundant soluble basic protein of the stylar transmitting tract in potato (Solanum tuberosum L.) is an endochitinase. Planta. 1994;194(2):264-73.

97. O'Riordain G, Radauer C, Hoffmann-Sommergruber K, Adhami F, Peterbauer C, Blanco C, et al. Cloning and molecular characterization of the Hevea brasiliensis allergen Hev b 11, a class I chitinase. Clinical & Experimental Allergy. 2002;32(3):455-62.

98. Martínez-Caballero S, Cano-Sánchez P, Mares-Mejía I, Díaz-Sánchez AG, Macías-Rubalcava ML, Hermoso JA, et al. Comparative study of two GH 19 chitinase-like proteins from Hevea brasiliensis, one exhibiting a novel carbohydrate-binding domain. The FEBS journal. 2014;281(19):4535-54.

99. Yano S, Rattanakit N, Honda A, Noda Y, Wakayama M, Plikomol A, et al. Purification and characterization of chitinase A of Streptomyces cyaneus SP-27: an enzyme participates in protoplast formation from Schizophyllum commune mycelia. Bioscience, biotechnology, and biochemistry. 2008;72(1):54-61.

100. Yano S, Honda A, Rattanakit-Chandet N, Noda Y, Wakayama M, Plikomol A, et al. Role of chitin binding domain of chitinase A of streptomyces cyaneus SP-27 in protoplast formation from schizophyllum commune. Bioscience, biotechnology, and biochemistry. 2009;73(3):733-5.

101. Sasaki C, Itoh Y, Takehara H, Kuhara S, Fukamizo T. Family 19 chitinase from rice (Oryza sativa L.): substrate-binding subsites demonstrated by kinetic and molecular modeling studies. Plant molecular biology. 2003;52(1):43-52.

102. Sticher L, Hofsteenge J, Neuhaus J-M, Boller T, Meins Jr F. Posttranslational Processing of a New Class of Hydroxyproline-Containing Proteins (Prolyl Hydroxylation and C-Terminal Cleavage of Tobacco (Nicotiana tabacum) Vacuolar Chitinase). Plant physiology. 1993;101(4):1239-47.

103. Iseli B, Boller T, Neuhaus J-M. The N-terminal cysteine-rich domain of tobacco class I chitinase is essential for chitin binding but not for catalytic or antifungal activity. Plant Physiology. 1993;103(1):221-6.

104. Freydl E, Meins F, Boller T, Neuhaus J-M. Kinetics of prolyl hydroxylation, intracellular transport and C-terminal processing of the tobacco vacuolar chitinase. Planta. 1995;197(2):250-6.

105. Iseli-Gamboni B, Boller T, Neuhaus J-M. Mutation of either of two essential glutamates converts the catalytic domain of tobacco class I chitinase into a chitin-binding lectin. Plant science. 1998;134(1):45-51.

106. Suarez V, Staehelin C, Arango R, Holtorf H, Hofsteenge J, Meins F. Substrate specificity and antifungal activity of recombinant tobacco class I chitinases. Plant molecular biology. 2001;45(5):609-18.

107. Fan J, Wang H, Feng D, Liu B, Liu H, Wang J. Molecular characterization of plantain class I chitinase gene and its expression in response to infection by Gloeosporium musarum Cke and Massee and other abiotic stimuli. Journal of biochemistry. 2007;142(5):561-70.

108. Orlando M, Pucciarelli S, Lotti M. Endolysins from Antarctic Pseudomonas Display Lysozyme Activity at Low Temperature. Marine Drugs. 2020;18(11):579.

109. Harikrishna K, Jampates-Beale R, Milligan SB, Gasser CS. An endochitinase gene expressed at high levels in the stylar transmitting tissue of tomatoes. Plant molecular biology. 1996;30(5):899-911.

110. Tsujibo H, Kubota T, Yamamoto M, Miyamoto K, Inamori Y. Characterization of chitinase genes from an alkaliphilic actinomycete, Nocardiopsis prasina OPC-131. Appl Environ Microbiol. 2003;69(2):894-900.

111. Fujimura T, Shigeta S, Suwa T, Kawamoto S, Aki T, Masubuchi M, et al. Molecular cloning of a class IV chitinase allergen from Japanese cedar (Cryptomeria japonica) pollen and competitive inhibition of its immunoglobulin E-binding capacity by latex C-serum. Clinical & Experimental Allergy. 2005;35(2):234-43.

112. Takashima T, Ohnuma T, Fukamizo T. NMR assignments and ligand-binding studies on a two-domain family GH19 chitinase allergen from Japanese cedar (Cryptomeria japonica) pollen. Biomolecular NMR assignments. 2017;11(1):85-90.

113. Takashima T, Numata T, Taira T, Fukamizo T, Ohnuma T. Structure and Enzymatic Properties of a Two-Domain Family GH19 Chitinase from Japanese Cedar (Cryptomeria japonica) Pollen. Journal of agricultural and food chemistry. 2018;66(22):5699-706.

114. López RC, Gómez-Gómez L. Isolation of a new fungi and wound-induced chitinase class in corms of Crocus sativus. Plant Physiology and Biochemistry. 2009;47(5):426-34.

115. Xiao Y-H, Li X-B, Yang X-Y, Luo M, Hou L, Guo S-H, et al. Cloning and characterization of a balsam pear class I chitinase gene (Mcchit1) and its ectopic expression enhances fungal resistance in transgenic plants. Bioscience, biotechnology, and biochemistry. 2007;71(5):1211-9.

116. Nakayama K, Takashima K, Ishihara H, Shinomiya T, Kageyama M, Kanaya S, et al. The R-type pyocin of Pseudomonas aeruginosa is related to P2 phage, and the F-type is related to lambda phage. Molecular microbiology. 2000;38(2):213-31.

117. Lim J-A, Shin H, Kang D-H, Ryu S. Characterization of endolysin from a Salmonella Typhimurium-infecting bacteriophage SPN1S. Research in microbiology. 2012;163(3):233-41.

118. Park Y, Lim JA, Kong M, Ryu S, Rhee S. Structure of bacteriophage SPN 1 S endolysin reveals an unusual two-module fold for the peptidoglycan lytic and binding activity. Molecular microbiology. 2014;92(2):316-25.

119. Oliveira H, Vilas Boas D, Mesnage S, Kluskens LD, Lavigne R, Sillankorva S, et al. Structural and enzymatic characterization of ABgp46, a novel phage endolysin with broad anti-Gram-negative bacterial activity. Frontiers in microbiology. 2016;7:208.

120. Lai M-J, Lin N-T, Hu A, Soo P-C, Chen L-K, Chen L-H, et al. Antibacterial activity of Acinetobacter baumannii phage ϕAB2 endolysin (LysAB2) against both gram-positive and gram-negative bacteria. Applied microbiology and biotechnology. 2011;90(2):529-39.

121. Peng S-Y, You R-I, Lai M-J, Lin N-T, Chen L-K, Chang K-C. Highly potent antimicrobial modified peptides derived from the Acinetobacter baumannii phage endolysin LysAB2. Scientific reports. 2017;7(1):11477.

122. Yamada T, Satoh S, Ishikawa H, Fujiwara A, Kawasaki T, Fujie M, et al. A jumbo phage infecting the phytopathogen Ralstonia solanacearum defines a new lineage of the Myoviridae family. Virology. 2010;398(1):135-47.

123. Hosoda N, Kurokawa Y, Sako Y, Nagasaki K, Yoshida T, Hiroishi S. The functional effect of Gly209 and Ile213 substitutions on lysozyme activity of family 19 chitinase encoded by cyanophage Ma-LMM01. Fisheries Science. 2011;77(4):665-70.

124. Kawase T, Yokokawa S, Saito A, Fujii T, Nikaidou N, Miyashita K, et al. Comparison of enzymatic and antifungal properties between family 18 and 19 chitinases from S. coelicolor A3 (2). Bioscience, biotechnology, and biochemistry. 2006;70(4):988-98.

125. Singh A, Kirubakaran SI, Sakthivel N. Heterologous expression of new antifungal chitinase from wheat. Protein expression and purification. 2007;56(1):100-9.

126. Ishisaki K, Honda Y, Taniguchi H, Hatano N, Hamada T. Heterogonous expression and characterization of a plant class IV chitinase from the pitcher of the carnivorous plant Nepenthes alata. Glycobiology. 2012;22(3):345-51.

127. Lu X, Wang B, Cai X, Chen S, Chen Z, Xin Z. Feeding on tea GH19 chitinase enhances tea defense responses induced by regurgitant derived from Ectropis grisescens. Physiologia Plantarum. 2020.

128. Kashyap P, Deswal R. A novel class I Chitinase from Hippophae rhamnoides: Indications for participating in ICE-CBF cold stress signaling pathway. Plant Science. 2017;259:62-70.

129. Shinshi H, Neuhaus J-M, Ryals J, Meins F. Structure of a tobacco endochitinase gene: evidence that different chitinase genes can arise by transposition of sequences encoding a cysteine-rich domain. Plant molecular biology. 1990;14(3):357-68.

130. Collinge DB, Kragh KM, Mikkelsen JD, Nielsen KK, Rasmussen U, Vad1 K. Plant chitinases. The Plant Journal. 1993;3(1):31-40.

131. Neuhaus J-M, Fritig B, Linthorst H, Meins F, Mikkelsen J, Ryals J. A revised nomenclature for chitinase genes. Plant Molecular Biology Reporter. 1996;14(2):102-4.

132. Kawase T, Saito A, Sato T, Kanai R, Fujii T, Nikaidou N, et al. Distribution and phylogenetic analysis of family 19 chitinases in Actinobacteria. Applied and environmental microbiology. 2004;70(2):1135-44.

133. Su Y, Xu L, Wang S, Wang Z, Yang Y, Chen Y, et al. Identification, phylogeny, and transcript of chitinase family genes in sugarcane. Scientific reports. 2015;5:10708.

134. Wasano N, Konno K, Nakamura M, Hirayama C, Hattori M, Tateishi K. A unique latex protein, MLX56, defends mulberry trees from insects. Phytochemistry. 2009;70(7):880-8.

135. Hossain MA, Noh H-N, Kim K-I, Koh E-J, Wi S-G, Bae H-J, et al. Mutation of the chitinase-like protein-encoding AtCTL2 gene enhances lignin accumulation in dark-grown Arabidopsis seedlings. Journal of plant physiology. 2010;167(8):650-8.

136. Kwon Y, Kim SH, Jung MS, Kim MS, Oh JE, Ju HW, et al. Arabidopsis hot2 encodes an endochitinase-like protein that is essential for tolerance to heat, salt and drought stresses. The Plant Journal. 2007;49(2):184-93.

137. Hermans C, Porco S, Verbruggen N, Bush DR. Chitinase-like protein CTL1 plays a role in altering root system architecture in response to multiple environmental conditions. Plant Physiology. 2010;152(2):904-17.

138. Zhang D, Hrmova M, Wan C-H, Wu C, Balzen J, Cai W, et al. Members of a new group of chitinase-like genes are expressed preferentially in cotton cells with secondary walls. Plant molecular biology. 2004;54(3):353-72.

139. Li D-M, Staehelin C, Wang W-T, Peng S-L. Molecular cloning and characterization of a chitinase-homologous gene from Mikania micrantha infected by Cuscuta campestris. Plant molecular biology reporter. 2010;28(1):90.

140. Davies GJ, Wilson KS, Henrissat B. Nomenclature for sugar-binding subsites in glycosyl hydrolases. Biochemical Journal. 1997;321(Pt 2):557.

141. Imoto T, Yagishita K. A simple activity measurement of lysozyme. Agricultural and Biological Chemistry. 1971;35(7):1154-6.

142. Boller T, Mauch F. Colorimetric assay for chitinase. Methods in enzymology. 161: Elsevier; 1988. p. 430-5.

143. Hoell IA, Klemsdal SS, Vaaje-Kolstad G, Horn SJ, Eijsink VG. Overexpression and characterization of a novel chitinase from Trichoderma atroviride strain P1. Biochimica et Biophysica Acta (BBA)-Proteins and Proteomics. 2005;1748(2):180-90.

144. Boller T, Gehri A, Mauch F, Vögeli U. Chitinase in bean leaves: induction by ethylene, purification, properties, and possible function. Planta. 1983;157(1):22-31.

145. Staehelin C, Schultze M, Tokuyasu K, Poinsot V, Promé J-C, Kondorosi É, et al. N-deacetylation of Sinorhizobium meliloti Nod factors increases their stability in the Medicago sativa rhizosphere and decreases their biological activity. Molecular plant-microbe interactions. 2000;13(1):72-9.
