# Supplementary material for: The GH19 Engineering Database: Sequence diversity, substrate scope, and evolution in glycoside hydrolase family 19
Source: PLoS One. 2021 Oct 26;16(10):e0256817. doi: 10.1371/journal.pone.0256817 (PMC8547705; doi:10.1371/journal.pone.0256817)
Supplement: S1 Table — These sequences were manually screened from literature starting from the entries reported in the “Characterized” and “Structure” tabs of the GH19 CAZy page (http://www.cazy.org/GH19.html). Sequences retrieved only from literature are listed in bold. Superfamily assignments are based on Fig 2. Subfamily group assignments and numeral identifiers are based on Fig 3A and 3B. hfam ID = group identifier (homologous family in GH19ED database). CBM = Carbohydrate binding module. (PDF) [file pone.0256817.s018.pdf]

Tables

Table S1. List of GH19 seed sequences, used for BLAST searches to initialize the GH19ED database. These sequences were manually screened from literature starting from the entries reported in the "Characterized" and "Structure" tabs of the GH19 CAZy page (http://www.cazy.org/GH19.html). Sequences retrieved only from literature are listed in bold. Superfamily assignments are based on **Fig. 2**. Subfamily group assignments and numeral identifiers are based on **Fig. 3A-B**. hfam ID = group identifier (homologous family in GH19ED database). CBM = Carbohydrate binding module.

Uniprot Accession	PDB Accession	Source	N° (CBM)	Subfamily (hfam ID)	Activity	Property	References
P29022	4mck	*Zea mais* (Plants)	1 (18)	CHIT (2a)	Chitinase, allergen	Antifungal	[1-3]
**O64203**		***Mycobacterium* phage D29 (Virus)**	**a**	**ELYS (8)**	**Lysozyme**		**[4]**
A9ZSX9	3wh1	*Gemmabryum coronatum* (Plants)		CHIT (2b)	Chitinase		[5-9]
Q9WXI9		*Aeromonas sp.* 10S24 (*Gammaproteobacteria*)	2 (5/12)	CHIT (7)	Chitinase		[10, 11]
**R0MMH7**		***Nosema bombycis* (*Fungi*)**	**b**	**CHIT (14)**	**Chitinase**		**[12]**
O50152	1wvu	*Streptomyces griseus* HUT 6037 (*Actinobacteria*)	1 (5/12)	CHIT (5)	Chitinase	Antifungal	[13-18]
Q9SQF7	2z37	*Brassica juncea* (Plants)	2 (18)	CHIT (1)	Chitinase, allergen		[16, 19]
Q9FRV0	4j0l	*Secale cereale* (Plants)		CHIT (1)	Chitinase	Antifungal	[20-25]
Q5J1K1		*Streptomyces sp.* MG3 (*Actinobacteria*)	1 (5/12)	CHIT (5)	Chitinase	Antifungal	[26]
Q25BT4		*Vibrio proteolyticus* (*Gammaproteobacteria*)	2 (5/12)	CHIT (6)	Chitinase		[27]
A4C3H5		*Pseudoalteromonas tunicata* (*Gammaproteobacteria*)	1 (5/12)	CHIT (6)	Chitinase	Antifungal	[28]
Q43752		*Citrus sinensis* (Plants)		CHIT (1)	Chitinase		[29]
Q9XFW7		*Beta vulgaris* (Plants)	1 (18)	CHIT (2a)	Chitinase	Antifungal	[30-32]
P25765		*Oryza sativa* (Plants)	1 (18)	CHIT (1)	Chitinase	Antifungal	[33]
Q9SAY3		*Oryza sativa* (Plants)	1 (18)	CHIT (1)	Chitinase		[34]
Q9FEW1		*Nicotiana tabacum* (Plants)	1 (18)	CHIT (1)	Chitinase, lysozyme		[35-37]
Q9AXR8		*Secale cereale* (Plants)		CHIT (1)		Ice growth inhibition	[38]
P85084	3cql	*Carica papaya* (Plants)		CHIT (1)	Chitinase		[39, 40]
P23951 or P11955	2baa	*Hordeum vulgare* (Plants)		CHIT (1)	Chitinase	Antifungal	[41-50]
Q7DNA1	31vr	*Oryza sativa* (Plants)	1 (18)	CHIT (1)	Chitinase	Antifungal	[51-54]
Q8MD06		*Leucaena leucocephala* (Plants)	1 (18)	CHIT (1)	Chitinase	Antifungal	[55]
Q42995		*Oryza sativa* (Plants)	1 (18)	CHIT (1)	Chitinase	Antibacterial (expression strain)	[56]
Q9AXR9		*Secale cereale* (Plants)	1 (18)	CHIT (1)		Ice growth inhibition	[38]
P42820		*Beta vulgaris* (Plants)	1 (18)	CHIT (2a)	Chitinase		[31, 57]
**Q4KHC5**		***Pseudomonas fluorescens* Pf-5 (prophage in bacteria)**		**ELYS (1)**	**Lysozyme**		**[58]**
A7UC81		*Oryza sativa* (Plants)	1 (18)	CHIT (1)	Chitinase		[59]
P11218[c]		*Urtica dioica* (Plants)	2 (18)	CHIT (4)	Allergen	Antifungal, insecticidal	[60-63]

V5TEI0		*Dionea muscipula* (Plants)	1 (18)	CHIT (1)	Chitinase	Insect digestion	[64]
Q96408		*Daucus carota* (Plants)	1 (18)	CHIT (2a)	Chitinase		[65]
G9B4E2		*Picea engelmannii* x *Picea glauca* (Plants)	1 (18)	CHIT (1)	Chitinase		[66]
Q9RHU5		*Streptomyces thermoviolaceus* OPC-520 (*Actinobacteria*)	1 (13)	CHIT (5)	Chitinase	Antifungal	[67]
**G3BM11**		***Salmonella enterica* phage PVPSE1 (Phage)**	**d**	**ELYS (3)**	**Lysozyme**	**Antibacterial**	**[68]**
Q59I46		*Bacillus circulans* (*Firmicutes*)	2 (5/12)	CHIT (5)	Chitinase		[69-71]
Q9Z9M4	2cjl	*Streptomyces coelicolor* A3 (*Actinobacteria*)		CHIT (5)	Chitinase		[72-74]
G9B4E3		*Picea engelmannii* x *Picea glauca* (Plants)	1 (18)	CHIT (1)	Chitinase		[66]
Q9RHU4		*Streptomyces thermoviolaceus* OPC-520 (*Actinobacteria*)		CHIT (5)	Chitinase	Antifungal	[67]
B1B6T0		*Bromus inermis* (Plants)	1 (18)	CHIT (1)	Chitinase	Plant cold-response	[75]
Q9FUH3	4tx7	*Vigna unguiculata* (Plants)	1 (18)	CHIT (1)	Chitinase	Antifungal	[76]
O04138		*Oryza sativa* (Plants)	1 (18)	CHIT (2a)	Chitinase	Antifungal	[77]
Q42428		*Castanea sativa* (Plants)	1 (18)	CHIT (1)	Chitinase	Antifungal	[78, 79]
O81934	1dxj	*Canavalia ensiformis* (Plants)		CHIT (1)	Chitinase		[80, 81]
P29023		*Zea mais* (Plants)	1 (18)	CHIT (2a)	Chitinase	Antifungal	[82]
**G9I9L2**		***Pseudomonas* phage OBP (Phage)**	**d**	**ELYS (5)**	**Lysozyme**	**Antibacterial**	**[68]**
B3VFX0		*Limonium bicolor* (Plants)	1 (18)	CHIT (1)	Chitinase	Antifungal	[83]
F8WSX8		*Chitiniphilus shinanonensis* (*Betaproteobacteria*)	2 (5/12)	CHIT (5)	Chitinase	Antifungal	[84]
O24530		*Vitis vinifera* (Plants)	1 (18)	CHIT (2a)	Chitinase		[85]
Q9FRV1		*Secale cereale* (Plants)	1 (18)	CHIT (1)	Chitinase	Antifungal	[20, 23, 86, 87]
P17513		*Nicotiana tabacum* (Plants)		CHIT (1)	Chitinase		[35, 37, 88]
Q9ZTT8		*Gossypium hirsutum* (Plants)	1 (18)	CHIT (1)	Chitinase		[89]
O23804		*Chenopodium amaranticolor* (Plants)	1 (18)	CHIT (2a)	Chitinase	Antibacterial (expression strain)	[90]
Q9LBM0		*Burkholderia gladioli* (*Betaproteobacteria*)	1 (5/12)	CHIT (5)	Chitinase		[91]
Q6WSR8	3hbe	*Picea abies* (Plants)	1 (18)	CHIT (2a)			[92, 93]
P19171		*Arabidopsis thaliana* (Plants)	1 (18)	CHIT (1)		Pathogen resistance	[94, 95]
Q43184		*Solanum tuberosum* (Plants)		CHIT (1)	Chitinase		[96]
O24531		*Vitis vinifera* (Plants)	1 (18)	CHIT (2a)	Chitinase		[85]
Q949H3	4mst	*Hevea brasiliensis* (Plants)	1 (18)	CHIT (1)	Allergen	Antifungal	[97, 98]
B3XZQ2		*Streptomyces cyaneus* SP-27 (*Actinobacteria*)	1 (5/12)	CHIT (5)	Chitinase	Antifungal (protoplast formation)	[99, 100]
P24626		*Oryza sativa* (Plants)	1 (18)	CHIT (1)	Chitinase	Antifungal	[77, 101]
P08252		*Nicotiana sylvestris* (Plants)	1 (18)	CHIT (1)	Chitinase, lysozyme	Antifungal	[36, 37, 102-106]
P17514		*Nicotiana tabacum* (Plants)		CHIT (1)	Chitinase, lysozyme		[35, 37, 88]
Q2HJJ5		*Musa paradisica* (Plants)	1 (18)	CHIT (1)	Chitinase	Antifungal	[107]
**A0A516Z9V1**		***Pseudomonas sp.* Ef1**		**ELYS**	**Lysozyme**		[108]

		**(prophage in bacteria)**		**(1)**			
Q42878		*Solanum lycopersicum* (Plants)		CHIT (1)	Chitinase		[109]
G9B4E8		*Pinus contorta* (Plants)	1 (18)	CHIT (1)	Chitinase		[66]
Q8GI53		*Nocardiopsis prasina* (*Actinobacteria*)	1 (5/12)	CHIT (5)	Chitinase	Antifungal	[110]
**A0A516Z9W0**		***Pseudomonas sp.* Ef1 (prophage in bacteria)**		**ELYS (1)**	**Lysozyme**	**Antibacterial**	[108]
Q5NTA4	5h7t	*Cryptomeria japonica* (Plants)	1 (18)	CHIT (2a)	Allergen, Chitinase	Antifungal	[111-113]
B5L6N2		*Crocus sativus* (Plants)	1 (18)	CHIT (1)	Chitinase	Antifungal	[114]
Q207U1		*Momordica charantia* (Plants)	1 (18)	CHIT (1)		Antifungal (transgenic)	[115]
**Q9S566**		***Pseudomonas aeruginosa* PAO1 (prophage in bateria)**		**ELYS (1)**	**Lysozyme**		**[116]**
H2D0G4	4ok7	*Salmonella phage* SPN1S (Phage)		ELYS (2)	Lysozyme	Antibacterial	[117, 118]
**A0A0M4F9K9**		***Acinetobacter* phage vB_AbaP_CEB1 (Phage)**		**ELYS (6)**	**Lysozyme**	**Antibacterial**	**[119]**
**F1BCP4**		***Acinetobacter* phage ΦAB2 (Phage)**		**ELYS (6)**	**Lysozyme**	**Antibacterial**	**[120, 121]**
B2ZY61		*Ralstonia* phage ΦRSL1 (Phage)		ELYS (4)		Cell shape modification (expression strain)	[122]
A0A7I3		*Microcystis aeruginosa* phage (Phage)		ELYS (7)	Chitinase, lysozyme		[123]
Q9Z9M6		*Streptomyces coelicolor* A3(2) (*Actinobacteria*)	1 (5/12)	CHIT (5)	Chitinase	Antifungal	[124]
Q9XEN3		*Triticum aestivum* (Plants)	1 (18)	CHIT (2a)	Chitinase	Antifungal	[125]
A9ZMK1		*Nepenthes alata* (Plants)	1 (18)	CHIT (2a)	Chitinase		[126]
**XP_028076454[e]**		***Camellia sinensis (Plants)***	**1 (18)**	**CHIT (1)**	**Chitinase**	**Defense against herbivore insects**	**[127]**
**H9CDX2**		***Hippophae rhamnoides (Plants)***	**1 (18)**	**CHIT (1)**	**Chitinase**	**Cold tolerance in the expression strain**	**[128]**

[a]This sequence contains an N-terminal amidase (no GH) domain and a C-terminal unclassified domain for host specific membrane binding.

[b]This sequence contains an N-terminal domain with unknown function, potentially involved in chitin binding.

[c]This sequence is also a chitinase-like protein listed in **Tab. S3**.

[d]These sequences possess an N-terminal putative peptidoglycan binding module, not yet described in terms of function and structure.

[e]NCBI GenBank identifier.
