# Supplementary material for: The GH19 Engineering Database: Sequence diversity, substrate scope, and evolution in glycoside hydrolase family 19
Source: PLoS One. 2021 Oct 26;16(10):e0256817. doi: 10.1371/journal.pone.0256817 (PMC8547705; doi:10.1371/journal.pone.0256817)
Supplement: S2 Table — h-fam ID = group identifier (homologous family in GH19ED database) based on Fig 3A and 3B. (PDF) [file pone.0256817.s019.pdf]

Table S2. List of GH19 subfamilies and groups defined in this study (**Figs. 2** and **3A-B**), their respective number of sequences and proteins (99% identity clustering of sequences), and the average number of residues in the catalytic domain ± standard deviation. h-fam ID = group identifier (homologous family in GH19ED database) based on **Fig. 3A-B**.

Superfamily	h-fam ID	Class-based classification[b]	Loop-based classification[b]	Cluster-based classification	N° sequences	Av. n° residues (±SD)
Chitinases (CHIT, 8554 sequences)	1	class I and II	plant "loopful"	Cluster I	1356	227 ± 30
	2a-b	class IV	plant "loopless"	Cluster II	1253	195 ± 17
	3	classes II-IV-VII			402	236 ± 24
	4	classes I-II-VI			31	226 ± 14
	5	class IV	bacteria "loopless"	Cluster II	1854	202 ± 9
	6 - 7			Cluster IV	2652	243 ± 19
	8 to 12				317	212 ± 21
	13 -14				33	146 ± 7
	15				18	161 ± 4
	16 -17				114	187 ± 18
	Unclassified[a]				524	
Endolysins (ELYS, 10967 sequences)	1			cluster III	3466	181 ± 17
	2			cluster III	2780	196 ± 11
	3			cluster III	10	179 ± 1
	4			cluster III	1	200
	5			cluster III	8	192 ± 14
	6			cluster III	24	163 ± 3
	7			cluster III	1	165
	8			cluster III	246	170 ± 4
	9 to 34			cluster III	1813	178 ± 17
	Unclassified[a]				2620	

[a]Sequences contained in smaller groups were not classified but were inserted in the database.

[b]The classification system from literature is based on [129-133].
