# Supplementary material for: The GH19 Engineering Database: Sequence diversity, substrate scope, and evolution in glycoside hydrolase family 19
Source: PLoS One. 2021 Oct 26;16(10):e0256817. doi: 10.1371/journal.pone.0256817 (PMC8547705; doi:10.1371/journal.pone.0256817)
Supplement: S3 Table — hfam ID = group identifier (homologous family in GH19ED database). (PDF) [file pone.0256817.s020.pdf]

Table S3. List of catalytically inactive chitinase-like GH19 proteins (CLP) from CHIT groups 3 and 4, shown also in **Fig. 3**. hfam ID = group identifier (homologous family in GH19ED database).

Uniprot Accession	PDB Accession	Source	N° (CBM)	hfam ID	Property	References
P11218		*Urtica dioica* (Plants)	2 (18)	4	Allergenic, antifungal, insecticidal	[60-63]
A0A059U3G8		*Morus alba* (Plants)	2 (18)	4	Latex protein toxic to herbivores	[134]
Q9LSP9		*Arabidopsis thaliana* (Plants)		3	Enhances lignin accumulation in etiolated seedlings	[135]
Q9MA41		*Arabidopsis thaliana* (Plants)		3	Essential for normal plant growth and development	[136, 137]
Q6JX03		*Gossypium hirsutum* (Plants)		3	Secondary wall deposition	[138]
ACO25187[a]		*Mikania micrantha* (Plants)		3	Accumulates in response to mechanical wounding and application of abscisic acid, salicylic acid, or $ZnSO_4$	[139]

[a]NCBI GenBank identifier.
