# Supplementary material for: The GH19 Engineering Database: Sequence diversity, substrate scope, and evolution in glycoside hydrolase family 19
Source: PLoS One. 2021 Oct 26;16(10):e0256817. doi: 10.1371/journal.pone.0256817 (PMC8547705; doi:10.1371/journal.pone.0256817)
Supplement: S4 Table — hfam ID = group identifier (homologous family in the GH19ED) based on Fig 3A and 3B. (PDF) [file pone.0256817.s021.pdf]

Table S4. Sequence entries from the GH19ED with degree greater than 300 (i.e. more than 300 neighboring sequences) in hub regions of the catalytic domains (at a threshold of 95% sequence identity) are listed with their corresponding annotation, taxonomic name of the source organism, and NCBI accession (compare with **Fig. S7**). hfam ID = group identifier (homologous family in the GH19ED) based on **Fig. 3A-B**.

**Degree**	**Annotation**	**Source organism**	**NCBI accession**	**Subfamily (hfam ID)**
301	glycoside hydrolase family 19 protein	*Pseudomonas aeruginosa*	WP_116825151.1	ELYS (1)
308	glycoside hydrolase family 19 protein	*Pseudomonas aeruginosa*	WP_123789282.1	ELYS (1)
355	carbohydrate-binding protein	*Vibrio atlanticus*	WP_065678462.1	CHIT (6)
364	carbohydrate-binding protein	*Vibrio owensii*	WP_122068128.1	CHIT (6)
376	carbohydrate-binding protein	*Vibrio splendidus*	WP_108214678.1	CHIT (6)
376	carbohydrate-binding protein	*Vibrio splendidus*	WP_102462326.1	CHIT (6)
376	carbohydrate-binding protein	*Vibrio splendidus*	WP_108123460.1	CHIT (6)
395	carbohydrate-binding protein	*Vibrio chagasii*	WP_128161859.1	CHIT (6)
395	carbohydrate-binding protein	*Vibrio splendidus*	WP_116870071.1	CHIT (6)
