# Supplementary material for: The GH19 Engineering Database: Sequence diversity, substrate scope, and evolution in glycoside hydrolase family 19
Source: PLoS One. 2021 Oct 26;16(10):e0256817. doi: 10.1371/journal.pone.0256817 (PMC8547705; doi:10.1371/journal.pone.0256817)
Supplement: S5 Table — Standard position numbering is according to the chitinase from rye seed (PDB accession 4j0l). Information is provided about the frequency of amino acids (if higher than 1%, up to the forth residue in descending order of frequency) at each site, and the respective function, if known from [25]. Standard positions corresponding to conserved sites in the ELYS subfamily (S6 Table) are highlighted in bold. Standard positions of the sequence pattern specific for CHITs are marked in red. (PDF) [file pone.0256817.s022.pdf]

Table S5. Sites with conservation score 5 (see *Methods* section in the main text) in the CHIT subfamily. Standard position numbering is according to the chitinase from rye seed (PDB accession 4j0l). Information is provided about the frequency of amino acids (if higher than 1%, up to the forth residue in descending order of frequency) at each site, and the respective function, if known from [25]. Standard positions corresponding to conserved sites in the ELYS subfamily (**Tab. S6**) are highlighted in bold. Standard positions of the sequence pattern specific for CHITs are marked in red.

**Standard position**	**> 90% non-gapped sequences[a]**	**Conserved residues**				**Function[b]**
6		V 38%	I 15%	L 4.4%		
11		F 58%	W 3.6%	Y 1.9%		
14		M 31%	I 18%	L 13%	F 1.2%	
18		R 45%	A 13%	K 2.4%	V 1.2%	
23		C 30%	A 4.4%			
28		F 58%	E 7.1%	A 2.9%	L 1.3%	
29		Y 86%	W 4.4%			
30		T 73%	S 11%	D 4.5%	N 1.5%	
31		Y 77%	R 13%	F 1.1%		
34		F 67%	L 25%			
37		A 90%				
44	X	F 80%	V 8.1%	L 5.6%	Y 1.3%	
54	X	K 63%	R 25%	M 3.8%	A 1.5%	
55	X	R 42%	K 35%	Q 15%	H 1.7%	
56	X	E 68%	S 23%	T 5.2%		
**58**	**X**	**A 80%**	**V 11%**	**I 4.2%**		
**59**	**X**	**A 63%**	**T 32%**			
**60**	**X**	**F 61%**	**M 19%**	**A 16%**		
61	X	F 53%	L 43%			
**62**	**X**	**A 82%**	**G 12%**	**T 1.8%**		
**63**	**X**	**H 52%**	**N 25%**	**Q 19%**		
64	X	F 35%	V 34%	T 15%	I 8.1%	
**66**	**X**	**H 54%**	**Q 34%**	**S 3.6%**	**F 1.6%**	
**67**	**X**	**E 91%**	**K 4.7%**			
**68**	**X**	**T 91%**	**S 5.4%**			
84	X	L 69%	Y 14%	F 12%	M 1.4%	
**89**	**X**	**E 94%**				**Catalytic base and substrate binding (-1)**
**96**	**X**	**Y 90%**	**K 1.7%**	**M 1%**		**Substrate binding (-1)**
**97**	**X**	**C 93%**	**V 1.5%**			
**105**	**X**	**C 91%**	**P 3.8%**	**G 1.2%**		
111	X	Y 99%				
**113**	**X**	**G 99%**				
**114**	**X**	**R 98%**	**K 1.4%**			
**115**	**X**	**G 99%**				
116	X	P 59%	A 38%			
**118**	**X**	**Q 91%**	**P 4.8%**	**M 3.2%**		**Substrate binding (+1)**
**120**	**X**	**S 84%**	**T 9.6%**	**Y 4.3%**		**Water coordination and substrate binding (-2)**
122	X	N 82%	H 15%			
**124**	**X**	**N 99%**				**Substrate binding (-2)**

**125**	**X**	**Y 99%**				
128	X	A 54%	F 36%	C 5.6%	I 1.8%	
129	X	G 62%	S 37%			
136	X	L 83%	G 15%			
137	X	L 89%	I 5.6%			
**140**	**X**	**P 100%**				
**143**	**X**	**V 91%**	**I 4.2%**	**L 3.8%**		
150	X	A 38%	N 33%	S 26%	G 1.5%	
**151**	**X**	**F 41%**	**L 32%**	**W 23%**	**M 1.8%**	
153	X	T 52	S 37%	A 8.3%	V 1.4%	
**154**	**X**	**A 85%**	**G 13%**	**S 1.3%**		
156	X	W 79%	F 18%	L 1.4%	Y 1.1%	
**158**	**X**	**W 66%**	**F 30%**	**Y 2.3%**		
159	X	M 42%	N 19%	L 15%	V 13%	
165		K 53%	T 16%	S 4.6%	Q 4.3%	
167		S 45%	T 22%	A 14%	N 10%	
169	X	H 60%	L 32%	R 4.2%	Q 1.5%	
171	X	V 66%	A 27%	I 2.5%	L 1.5%	
**190**	**X**	**G 95%**	**N 1.8%**			
191	X	F 84%	Y 13%			
**192**	**X**	**G 95%**	**A 3.5%**			
194	X	T 81%	I 10%	V 4.4%	S 3.1%	
**195**	X	**I 63%**	**T 29%**	**M 4.3%**	**V 2.5%**	
196	X	N 40%	R 35%	Q 16%	K 2.4%	
197	X	I 45%	S 19%	A 16%	V 13%	
**198**	**X**	**I 88%**	**L 8.2%**	**V 2.1%**		**Substrate binding without side chain (-2)**
**199**	**X**	**N 92%**	**Y 4.8%**			**Substrate binding (-2)**
**200**	**X**	**G 94%**	**S 2.3%**	**A 1.8%**		
203	X	E 92%	V 2.9%			Substrate binding (+1)
204	X	C 97%				
**215**	**X**	**R 92%**	**I 4.3%**			**Substrate binding (+1)**
216	X	I 66%	V 29%	Y 1.1%	A 1.0%	
219	X	Y 75%	W 15%	F 7.7%		
**222**	**X**	**F 39%**	**Y 34%**	**L 15%**	**I 4.6%**	
223	X	A 35	C 31%	T 17%	L 6.4%	
228	X	V 69%	I 15%	T 8.9%	W 1.8%	
231		G 60%	D 15%	P 7.5%	N 7.3%	
236		C 85%				

[a]All CHIT sequences classified in the GH19ED database were considered.

[b]Binding subsites (in parenthesis) are numbered according to the standard nomenclature; cleavage occurs between the sugar units bound at subsites -1 and +1 [140].
