# Supplementary material for: The GH19 Engineering Database: Sequence diversity, substrate scope, and evolution in glycoside hydrolase family 19
Source: PLoS One. 2021 Oct 26;16(10):e0256817. doi: 10.1371/journal.pone.0256817 (PMC8547705; doi:10.1371/journal.pone.0256817)
Supplement: S6 Table — Standard position numbering is according to the endolysin from bacteriophage SPN1S of Salmonella typhimurium (PDB accession 4ok7). Information is provided about the frequency of amino acids (if higher than 1%, up to the forth residue in descending order of frequency) at each site, and the respective function, if known from [118]. Standard positions corresponding to conserved sites in the CHIT subfamily (S5 Table) are highlighted in bold. Standard positions of the sequence pattern specific for ELYSs are marked in red. (PDF) [file pone.0256817.s023.pdf]

Table S6. Sites with conservation score 5 (see *Methods* section in the main text) in the ELYS subfamily. Standard position numbering is according to the endolysin from bacteriophage SPN1S of *Salmonella typhimurium* (PDB accession 4ok7). Information is provided about the frequency of amino acids (if higher than 1%, up to the forth residue in descending order of frequency) at each site, and the respective function, if known from [118]. Standard positions corresponding to conserved sites in the CHIT subfamily (**Tab. S5**) are highlighted in bold. Standard positions of the sequence pattern specific for ELYSs are marked in red.

Standard position	> 90% non gapped sequences[a]	Conserved residues				Function
**33**	**X**	**I 80**	**L 3.3%**	**C 3.2%**	**V 2.3%**	
38	X	R 60%	D 22%	W 4.5%	Q 3.5%	
**40**	**X**	**A 90%**	**S 2.1%**	**C 1.8%**		
**41**	**X**	**A 36%**	**M 34%**	**Y 9.3%**	**Q 1.8%**	
**42**	**X**	**F 82%**	**W 4.5%**	**M 3.7%**	**I 2.4%**	
**44**	**X**	**A 91%**	**G 4.3%**	**S 3.4%**		
**45**	**X**	**Q 88%**	**T 9.3%**			
**48**	**X**	**H 93%**	**V 2.3%**			
**49**	**X**	**E 99%**				**Catalytic proton donor**
**50**	**X**	**S 86%**	**T 11%**	**C 1.4%**		
53	X	L 44%	F 43%	M 7.3%	G 1.6%	
**58**	**X**	**E 99%**				**Catalytic base**
61		N 53%	G 15%	S 8.9%	A 4.4%	
63		S 47%	T 17%	A 16%	Q 7.5%	
102	X	A 62%	Q 3.6%	L 3.5%	S 2.5%	
**106**	**X**	**Y 95%**	**F 2.3%**			
**109**	**X**	**R 80%**	**E 5.4%**	**A 4.2%**	**K 3.9%**	
110	X	L 51%	M 25%	N 16%	Y 2.3%	
111	X	G 94%	V 1.6%			
112		N 83%	D 10%			
116	X	G 83%	T 5.8%	D 3%	A 2%	
117	X	D 93%	E 2.6%			
**118**	**X**	**G 97%**	**Y 1.4%**			
122	X	R 79%	K 9.3%	L 3.8%	Y 1.9%	
**123**	**X**	**G 99%**				
**124**	**X**	**R 95%**	**G 1.2%**	**A 1.2%**		
**125**	**X**	**G 96%**	**T 2.5%**			
126	X	L 75%	P 7.7%	Y 4.3%		
127	X	I 63%	L 14%	V 8.9%	K 7.3%	
**128**	**X**	**Q 92%**	**M 5.5%**	**G 1%**		
**130**	**X**	**T 99%**				**Water coordination**
131	X	G 85%	F 5.3%	W 4.3%	H 4.2	
**134**	**X**	**N 96%**				
**135**	**X**	**Y 97%**	**F 1.5%**			
**150**	**X**	**P 95%**	**G 1.2%**			
**153**	**X**	**L 74%**	**A 13%**	**V 7.8%**	**I 2.2%**	
159	X	A 82%	S 6.2%	G 5.5%	E 2.2%	
**163**	**X**	**A 83%**	**S 4.2%**	**E 1.9%**	**G 1.5%**	
**167**	**X**	**W 65%**	**F 14%**	**Y 10%**	**T 8%**	
172	X	L 60%	C 29%	I 4.5%		
**173**	**X**	**L 54%**	**Y 23%**	**F 4.5%**	**W 2.5%**	
176		R 19%	K 14%	A 11%	S 11%	

**183**	**X**	**T 81%**	**R 9.6%**	**S 3.6%**	**G 3%**
**186**	**X**	**I 85%**	**V 12%**		
**187**	**X**	**N 97%**			
**188**	**X**	**G 89%**	**L 3.3%**	**P 1.5%**	**S 1.1%**
189		G 83%	A 2.1%	R 1.1%	T 1.1%
191		N 75%	T 3.4%	E 2.7%	H 1.6%
192		G 85%			
**196**	**X**	**R 89%**			
203		A 74%	I 3.6%	C 2.1%	V 1%

[a]All ELYS sequences classified in the GH19ED database were considered.
