# Supplementary material for: The GH19 Engineering Database: Sequence diversity, substrate scope, and evolution in glycoside hydrolase family 19
Source: PLoS One. 2021 Oct 26;16(10):e0256817. doi: 10.1371/journal.pone.0256817 (PMC8547705; doi:10.1371/journal.pone.0256817)
Supplement: S7 Table — The “loopful” plant chitinase from rye seed (PDB accession 4j0l) is taken as reference. Conservation score ranges from 1 (least conserved) to 5 (most conserved). Substrate binding residues that are present in the reference chitinase are highlighted in bold. (PDF) [file pone.0256817.s024.pdf]

Table S7. **Loop conservation scores at CHIT standard positions**. The "loopful" plant chitinase from rye seed (PDB accession 4j0l) is taken as reference. Conservation score ranges from 1 (least conserved) to 5 (most conserved). Substrate binding residues that are present in the reference chitinase are highlighted in bold.

Loop	Site	Conservation score	Average conservation score
1	20	1	2.1
	21	1	
	22	1	
	23	5	
	24	1	
	25	3	
	26	2	
	27	1	
2	**70**	**3**	1.2
	71	1	
	**72**	**1**	
	73	1	
	74	1	
	75	1	
	76	1	
	77	1	
	78	1	
	79	2	
	80	1	
	81	1	
	82	1	
	83	1	
3	94	1	2.7
	95	1	
	**96**	**5**	
	97	5	
	98	2	
	99	1	
	100	2	
	101	1	
	102	2	
	103	4	
	104	4	
	105	5	
	106	3	
	107	2	
4	160	4	3.9
	161	4	
	**162**	**4**	
	163	2	
	164	3	
	165	5	
	166	4	
	167	5	
	168	4	
5	174	3	1.1
	175	1	
	176	1	
	177	1	

	178	1	
	179	1	
	180	1	
	181	1	
	182	1	
	183	1	
	184	1	
	185	1	
	186	1	
	187	1	
	188	1	
C terminal	236	5	1.5
	237	1	
	238	1	
	239	1	
	240	1	
	241	1	
	**242**	**1**	
	243	1	
