# Supplementary material for: The GH19 Engineering Database: Sequence diversity, substrate scope, and evolution in glycoside hydrolase family 19
Source: PLoS One. 2021 Oct 26;16(10):e0256817. doi: 10.1371/journal.pone.0256817 (PMC8547705; doi:10.1371/journal.pone.0256817)
Supplement: S8 Table — Subfamily groups assignment and numeral identifiers are based on Fig 3A and 3B. The sequences in which the effect of CBMs on catalytic and antifungal activity was tested are highlighted in bold. The Uniprot accession of sequences in which the activity was tested on insoluble chitin, on soluble chitin polymers or oligomers (derivatives comprised), and on chitosan are underlined and the references for the protocols used are reported in the notes. hfam ID = group identifier (homologous family in GH19ED database). (PDF) [file pone.0256817.s025.pdf]

Table S8. List of the substrates in which GH19 CHIT seed sequences are active with notes on their activity. Subfamily groups assignment and numeral identifiers are based on **Fig. 3A-B**. The sequences in which the effect of CBMs on catalytic and antifungal activity was tested are highlighted in bold. The Uniprot accession of sequences in which the activity was tested on insoluble chitin, on soluble chitin polymers or oligomers (derivatives comprised), and on chitosan are underlined and the references for the protocols used are reported in the notes. hfam ID = group identifier (homologous family in GH19ED database).

Uniprot Accession	N° (CBM)	hfam ID	Substrates	Relevant notes[a]
**P29022**	**1 (18)**	**2a**	**Chitin from crab shells, glycol chitin; (GlcNAc)$_5$, (GlcNAc)$_4$**	**The CBM-truncated variant binds crab shell chitin and chitin agarose with an affinity that resembles the wild-type**
A9ZSX9		2b	Glycol-chitin; (GlcNAc)$_6$, (GlcNAc)$_5$, (GlcNAc)$_4$ and (GlcNAc)$_3$	No antifungal activity. At least 10 times more active on oligomers than Q9FRV0, and 3 times more active on glycol-chitin
**Q9WXI9**	**2 (5/12)**	**7**	**Colloidal chitin, crystalline chitin, ethylene glycol chitin; glycol chitin; (GlcNAc)$_6$**	**(GlcNAc)$_6$ hydrolysed mainly at the 4th residue from the reducing end. Hydrolytic activities toward both insoluble and soluble substrates were significantly reduced by the truncation of the two CBMs, with the stronger effects on more crystalline substrates and on the soluble (GlcNAc)$_6$. In addition, also antifungal activity decreased by the same truncation**
R0MMH7	NA	14	Ethylene glycol chitin; soluble chitosan (80 deacetylated); chitin beads; insoluble colloidal chitin; (GlcNAc)$_{3-5}$	(GlcNAc)$_{3-5}$ hydrolysis measured by HPLC; activity on other substrates was measured by the amount of reducing sugars produced with a modified Schale's procedure [141]. Very low activity toward crystalline substrates
**O50152**	**1 (5/12)**	**5**	**Colloidal chitin, chitosan 7B (70% deacetylated chitin), chitin EX, squid chitin, powdered chitin from crab shells, microcrystalline β-chitin, WS-chitin (water soluble); glycol chitin; colloidal chitin; (GlcNAc)$_{4-5}$**	**Activity on soluble chitin polymers is higher than on (GlcNAc)$_5$, 10-fold higher than on colloidal chitin and even more than on chitosan. Activity on crystalline polymers is much lower. (GlcNAc)$_4$ hydrolysis measured by HPLC. Activity on polymeric and oligomeric substrates was measured by the amount of reducing sugars produced with a modified Schale's procedure [141]. The CBM binds, in order of decreasing affinity, to insoluble chitin, soluble chitin, cellulose, and (GlcNAc)$_6$. Its deletion reduces the antifungal activity 10-fold and catalytic activity toward soluble and insoluble chitin substrates up to more than twice, except chitosan and (GlcNAc)$_5$. The CBM binds strongly to insoluble chitin, while a relatively lower affinity was measured for cellulose. The interaction with chitin depends on the degree of acetylation. The CBM has no antifungal activity by its own**
**Q9SQF7**	**2 (18)**	**1**	**Carboxymethylchitin-Remazol Brilliant Violet 5R (chitin azure)**	**Agglutination of rabbit erythrocytes in presence of both CBMs. This property is abolished if CBMs are cut out**
Q9FRV0		1	Glycol chitin, colloidal chitin, (GlcNAc)$_{4-6}$	Antifungal activity due to binding of fungal hyphal tips
Q5J1K1	1 (5/12)	5	Colloidal chitin, powdered crab chitin	
Q25BT4	2 (5/12)	6	Ethylene glycol chitin, colloidal chitin, β-chitin; (GlcNAc)$_{4-6}$	Lower activity on colloidal chitin with respect to other polymeric substrates, even if insoluble. Exo-mode of action

				preferentially from reducing end of free chains, hydrolysing mainly the second linkage from the reducing end
A4C3H5	1<br>(5/12)	6	Colloidal and crystalline chitin; 4-Nitrophenyl N-acetyl-β-D-glucosaminide (pNP- GlcNAc)	Optimum of activity at 2M NaCl (halophilic source organism)
Q43752		1	colloidal [3H] chitin	
Q9XFW7	1<br>(18)	2a	Colloidal chitin; lipochitooligosaccharides with $(GlcNAc)_{5-4}$ (LCO-$(GlcNAc)_n$, also known as NOD factors)	Hydrolysis of exposed chitin in the fungal hyphal apex to release $(GlcNAc)_{2-4}$. More active on not sulfated reducing end of LCO-$(GlcNAc)_4$
P25765	1<br>(18)	1	Glycol chitin	
Q9SAY3	1<br>(18)	1	Colloidal chitin	
Q9FEW1	1<br>(18)	1	Colloidal chitin	
Q9AXR8		1		Ice-binding
P85084		1	chitosan (75 ÷ 85% deacetylated); $(GlcNAc)_4$	
P23951 or P11955		1	4-methylumbelliferyl-N,N^{I},N^{II}-triacetyl-β-D-chitotrioside (MU-$(GlcNAc)_3$); pNP-$(GlcNAc)_5$; $(GlcNAc)_{6-4}$	MU-$(GlcNAc)_3$ mostly hydrolysed in $(GlcNAc)_2$ and MU-GlcNAc; pNP-$(GlcNAc)_5$ predominantly hydrolysed in $(GlcNAc)_2$ and pNP-$(GlcNAc)_3$
Q7DNA1	1<br>(18)	1	Glycol chitin, colloidal chitin, β-chitin, water soluble soluble chitin (38,8% deacetylated); $(GlcNAc)_{6-5}$	Activity on soluble chitin is higher than on chitooligomers or glycol chitin or colloidal chitin. Antifungal activity is slightly weaker than for O50152
Q8MD06	1<br>(18)	1	Chitin, swollen chitin, glycol chitin, colloidal chitin, chitosan; pNP-$(GlcNAc)_3$	Activity was determined by the protocol reported in [142]. This enzyme has a broad spectrum of antifungal potential
Q42995	1<br>(18)	1	[3H]-chitin	
Q9AXR9	1<br>(18)	1		Ice-binding
P42820	1<br>(18)	2a	Colloidal chitin; LCO-$(GlcNAc)_{5-4}$	Hydrolysis of exposed chitin in the fungal hyphal apex to release $(GlcNAc)_{2-4}$<br>More active on not sulfated reducing end of LCO-$(GlcNAc)_5$
A7UC81	1<br>(18)	1	Chitin	
**P11218**	**2<br>(18)**	**4**		**A precursor from which GH19 catalytic domain is removed to form an agglutinin with antifungal, insecticidal activity, and superantigenic properties in a mouse model, inducing the exclusive proliferation of Vbeta8.3(+) T lymphocytes.**
V5TEI0	1<br>(18)	1	α-chitin from shrimp shells; MU-(GlcNAc$)_3$	Slow hydrolysis of crystalline substrate. Proline residues and glycosylation predicted to be important in the capacity to resist proteolytic activity of cysteine protease in the digestive fluid of the source organism (fly trap)
Q96408	1<br>(18)	2a	Colloidal [3H]chitin	
G9B4E2	1<br>(18)	1	Chitin azure	Inactive on 4-MU labelled chitooligosaccharides (by measuring fluorescence) and colloidal chitin by using a reducing sugar detection assay
**Q9RHU5**	**1<br>(13)**	**5**	**Colloidal chitin, ethylene glycol chitin**	**Binding avicel, chitin and xylan with more affinity than Q9RHU4, which does not have any CBM, and has also a superior antifungal activity**
Q59I46	2<br>(5/12)	5	Ethylene glycol chitin	
Q9Z9M4		5	α-chitin, β-chitin, chitin azure, chitosan (64, 50, 32, and 13% acetylated); $(GlcNAc)_{6-3}$	Activity on chitin and $(GlcNAc)_{6-3}$ measured by HPLC. Activity on chitin azure measured as indicated in [143]. Chitosan activity was determined according to [74]. Identification of chitosan products was determined by size

				exclusion chromatography and NMR spectroscopy. Degradation of chitosan with varying degrees of acetylation to maximum degree of scission produced a wide variety of oligomer mixtures, differing in chain length and composition of acetylated/deacetylated units. Inactive on 4-MU labelled chitooligosaccharides
G9B4E3	1 (18)	1	Chitin azure	Inactive on 4-MU labelled chitooligosaccharides and colloidal chitin by using a reducing sugar detection assay
Q9RHU4		5	Colloidal chitin, ethylene glycol chitin	
B1B6T0	1 (18)	1	Ethylene glycol chitin	
Q9FUH3	1 (18)	1	colloidal chitin	Inactive on 4-NP labelled chitooligosaccharides
O04138	1 (18)	2a	Glycol chitin, colloidal chitin	More active on chitooligosaccarides than P24626, but lower fungal growth inhibition. This difference is not correlated to the presence of the CBM.
**Q42428**	**1 (18)**	**1**	**chitin azure**	**The inactivated mutant causes morphological alteration of hyphal tips. The chitin-binding domain itself can disturb the balance between chitin synthesis and hydrolysis at the apical growth zones**
O81934		1	chitin azure	
P29023	1 (18)	2a	colloidal chitin	
<u>B3VFX0</u>	1 (18)	1	NxO-carb-chitin, NxO-carb-chitosan, colloidal chitin, chitosan, chitin from cell wall extracted from various fungal species	Activity on chitin and colloidal chitosan is similar. Activity measured by the amount of reducing sugars produced with a modified Schale's procedure [141]
**<u>F8WSX8</u>**	**2 (5/12)**	**5**	**Soluble chitosan (80% deacetylation), colloidal chitin, insoluble powder chitin; pNP-$(GlcNAc)_3$, $(GlcNAc)_6$**	**pNP-$(GlcNAc)_3$ hydrolysed in $(GlcNAc)_2$ and pNP-GlcNAc; higher activity on soluble substrates. Much higher activity on soluble chitosan than on other insoluble substrates; CBMs improve antifungal activity and increased more than 4 times the hydrolytic activity toward colloidal and powder polymeric chitin, but not on soluble chitosan. Activity on polymeric substrates measured by the amount of reducing sugars produced with a modified Schales' procedure [141]. pNP-$(GlcNAc)_3$, $(GlcNAc)_6$ hydrolysis products separated by thin layer chromatography and analysed by UPLC**
O24530	1 (18)	2a	[3H]-chitin	
**Q9FRV1**	**1 (18)**	**1**	**Glycol chitin, colloidal chitin, chitin beads, cell walls of *Trichoderma* sp.; $(GlcNAc)_{6-5}$, slow hydrolysis of $(GlcNAc)_{4-2}$**	**Binds to lateral walls and septa, not just at hyphal tips. If the tryptophan residue inside the CBM (important for the chitin binding capacity of this domain) is mutated, activity on soluble and insoluble polymeric substrates and chitin-binding ability (at increasing salt concentration) decrease by 40-50%, with similar values of the paralogue Q9FRV0, which has half the activity of this enzyme on colloidal chitin. By removing or mutating the CBM, the antifungal activity is lower than Q9FRV0, but the fungal cell-wall degradation remains similar. The CBM alone does not possess antifungal activity on its own**

<u>P17513</u>		1	Chitin, chitosan (13.5% acetylated); $(GlcNAc)_{6-4}$	Activity on chitin and soluble chitooligomers is lower than for the paralogue P08252, and on chitosan even less. Activity on chitin is more than 3-fold higher than on chitosan. Activity was determined by the protocol reported in [142]. Not active on 4-MU-$(GlcNAc)_3$ and on *Micrococcus lisodeikticus* cells
Q9ZTT8	1 (18)	1	ethylene glycol chitin	
O23804	1 (18)	2a		Hydrolytic activity of bacterial cell walls of the host used for its heterologous expression (*E. coli*)
Q9LBM0	1 (5/12)	5	chitosan with moderate degree of acetylation (30%)	
**Q6WSR8**	**1 (18)**	**2a**	**Chitin azure**	**In presence of the CBM, the inhibition of fungal growth is more efficient. The hydrolytic activity of the truncated variant on chitin azure is the same**
P19171	1 (18)	1		It participates in the resistance to jasmonate-inducing pathogens, such as *Alternaria brassicicola*
Q43184		1	Chitin azure	
O24531	1 (18)	2a	[3H]-chitin	
**Q949H3**	**1 (18)**	**1**	**Chitin azure (after re-activating mutation)**	**Antifungal activity is present also in absence of the activity restoring mutation. Probably binds the nascent chitin of the hyphal apex along fungal cell walls and accumulates on new septa at hyphal tips. This binding produces changes in morphology including abnormal branching, shorter and swelling hyphae. Possesses strong binding affinity to chitin-beads and chitotriose**
**B3XZQ2**	**1 (5/12)**	**5**	**Colloidal chitin, powdered chitin, ethylene glycol chitin; $(GlcNAc)_{6-4}$**	**The CBM enhances the hydrolysis of insoluble chitin and the protoplast-forming activity. In the truncated variant, most of the binding affinity on colloidal chitin and powder chitin is also lost**
**P24626**	**1 (18)**	**1**	**Glycol chitin, colloidal chitin; $(GlcNAc)_{6-4}$**	**The inhibition of fungal growth is stronger in the wild-type enzyme than in the CBM defective mutant. The CBM does not significantly participate in oligosaccharide hydrolysis, but the activity on colloidal chitin is lower. The degradation of glycol chitin and $(GlcNAc)_{6-4}$ is less efficient than for O04138, a "loopless" paralogue**
**<u>P08252</u>**	**1 (18)**	**1**	**[3H]-chitin, chitin, chitosan (13.5% acetylated), radioactive colloidal chitin, LCO-$(GlcNAc)_5$ (C16:2, S) from *Rhizobium meliloti*, *Mycrococcus lysodeikticus* cells; $(GlcNAc)_{4-6}$; MU-(GlcNAc)3**	**The truncated enzyme has 50% lower activity on [3H]-chitin and is 3 times less effective in inhibiting fungal growth. Anyway, it has similar activity on radioactive colloidal chitin. Increasing the length of the spacer reduces dramatically hydrolysis of chitin and NOD factors, but not the lysozyme activity, which is 10-fold lower than the hen egg white lysozyme. The catalytic domain can be turned into a chitin-binding lectin by the artificial mutation of essential catalytic residues; chitosan hydrolysed at a similar rate of chitin; the hydrolytic activity requires at least a partial N-acetylation of glucosamine moieties. Activity on [3H]-chitin was measured according to [144]. $(GlcNAc)_{4-6}$ hydrolysis analysed by HPLC, LCO-**

				**(GlcNAc)$_5$ hydrolysis measured by the technique reported in the protocol from [145]**
P17514		1	Chitin, chitosan (13.5% acetylated), (GlcNAc)$_{4-6}$	Activity on chitin and chitooligomers is lower than for P08252. Activity on chitin is more than 3-fold higher than on chitosan. Activity was determined by the protocol reported in [142]. Not active on 4-MU-(GlcNAc)$_3$ and *Micrococcus lisodeikticus* cells
Q2HJJ5	1 (18)	1	Chitin azure	Antifungal
Q42878		1	Colloidal chitin	
G9B4E8	1 (18)	1	Chitin azure	Inactive on 4-MU-chitooligosaccharides and colloidal chitin by using a reducing sugar detection assay
**Q8GI53**	**1 (5/12)**	**5**	**Colloidal chitin and glycol chitin**	**The CBM permits to increase the antifungal activity and up to 3.9-fold the hydrolysis efficiency on soluble and insoluble polymeric chitin. Only the wild-type enzyme has strong binding affinity to α-chitin and β-chitin, but weak affinity for Avicel**
**Q5NTA4**	**1 (18)**	**2a**	**Glycol chitin, β-chitin nanofiber; (GlcNAc)$_{4-6}$**	**Human IgE binding capacity; the CBM domain is demonstrated to recognize insoluble chitin. Truncation of the CBM does not significantly affect the mode of (GlcNAc)$_{4-6}$ hydrolysis. However, this significantly enhances activity toward the soluble substrate, but suppresses activity toward the insoluble substrate. The catalytic activity appears essential for antifungal activity, and the presence of the CBM significantly improves it**
**B5L6N2**	**1 (18)**	**1**	**Chitin azure**	**The catalytic glutamate is substituted by a tyrosine, although it seems to be non-essential for the catalysis of this enzyme. By artificially substituting it with a glutamate, activity slightly increases. The catalytic domain alone retains the antifungal activity, but it is lower than for the complete enzyme**
Q207U1	1 (18)	1		Increased resistance to fungal phytopathogens if expressed in transgenic plants
Q9Z9M6	1 (5/12)	5	Glycol chitin, colloidal chitin, soluble chitin, powdered prawn shell chitin (chitin EX), highly crystalline β-chitin microfibrils, chitosan 7B (70% deacetylated)	High activity on soluble substrates; activity on colloidal chitin and chitosan is similar and one order of magnitude higher than on crystalline chitin substrates, which are hydrolysed with lower activity with respect to other chitinases coded from paralogue genes from the same organism. Activity on all substrates was measured by the amount of reducing sugars produced with a modified Schale's procedure [141]
Q9XEN3	1 (18)	2a	Colloidal chitin	100 μg treatment with this enzyme showed a broad-spectrum antifungal activity.
A9ZMK1	1 (18)	2a	Glycol chitin, colloidal chitin, ethylene glycol chitin; (GlcNAc)$_{4-6}$	Weak activity toward polymeric substrates
XP_028076454[b]	1 (18)	1	Colloidal chitin	Participates in a singular defense mechanism against plant pathogens that involves the regurgitant of the herbivore insect
H9CDX2	1 (18)	1	Colloidal chitin	

[a]References are reported in Tab. 1

[b]NCBI GenBank identifier.
