# Supplementary material for: The GH19 Engineering Database: Sequence diversity, substrate scope, and evolution in glycoside hydrolase family 19
Source: PLoS One. 2021 Oct 26;16(10):e0256817. doi: 10.1371/journal.pone.0256817 (PMC8547705; doi:10.1371/journal.pone.0256817)
Supplement: S9 Table — (PDF) [file pone.0256817.s026.pdf]

Table S9. List of referenced CBM properties demonstrated by point mutation, by truncation variants, or by comparing two similar enzymes from the same organism, with and without the CBM.

Property	Sub-property	N° of sequences	Uniprot accession	References
Increased antifungal activity on tested strains	In presence of the catalytic domain	12	Q9WXI9	[11]
			O50152	[15]
			Q9RHU5	[67]
			F8WSX8	[84]
			Q9FRV1	[23, 86, 87]
			Q6WSR8	[93]
			B3XZQ2	[99]
			P24626	[77]
			P08252	[106]
			Q8GI53	[110]
			Q5NTA4	[113]
			B5L6N2	[114]
	In absence of the catalytic activity	3	P11218	[61]
			Q42428	[79]
			Q949H3	[98]
Increased activity on insoluble chitinolytic substrates		5	P24626	[77]
			F8WSX8	[84]
			B3XZQ2	[100]
			P08252	[106]
			Q5NTA4	[113]
	Activity increased also on soluble polymers	3	O50152	[15]
			Q9FRV1	[20, 87]
			Q8GI53	[110]
	Activity increased also on soluble oligomers	1	Q9WXI9	[11]
Increased binding affinity for different forms of crystalline chitin		7	O50152	[15]
			Q9RHU5	[67]
			Q9FRV1	[87]
			Q949H3	[98]

			B3XZQ2	[100]
			Q8GI53	[110]
			Q5NTA4	[113]
No significant effect on insoluble chitin binding		1	P29022	[2]
Allergenic properties	Binding of human IgE in allergic patients	3	P29022	[3]
			Q949H3	[97]
			Q5NTA4	[111]
	Agglutination of rabbit erythrocytes	1	Q9SQF7	[16]
	Inducing the proliferation of Vbeta8.3(+) T lymphocytes	1	P11218	[62, 63]
